# New Quinoline–Urea–Benzothiazole Hybrids as Promising Antitubercular Agents: Synthesis, In Vitro Antitubercular Activity, Cytotoxicity Studies, and In Silico ADME Profiling

**DOI:** 10.3390/ph15050576

**Published:** 2022-05-05

**Authors:** Rashmika Moodley, Chakes Mashaba, Goitsemodimo H. Rakodi, Nomagugu B. Ncube, Mabuatsela V. Maphoru, Mohammed O. Balogun, Audrey Jordan, Digby F. Warner, Rene Khan, Matshawandile Tukulula

**Affiliations:** 1 School of Chemistry and Physics, University of KwaZulu Natal, Westville Campus, Durban 4000, South Africa; rashmika.moodley@za.ab-inbev.com (R.M.); machakes@gmail.com (C.M.); 217075576@stu.ukzn.ac.za (N.B.N.); 2 Department of Chemistry, Tshwane University Technology, Pretoria 0001, South Africa; rakodigh@tut.ac.za (G.H.R.); maphorum@tut.ac.za (M.V.M.); 3 Bio-Polymer Modification and Therapeutics Laboratory, Council for Scientific and Industrial Research (CSIR), Pretoria 0001, South Africa; mbalogun@csir.co.za; 4 SAMRC/NHLS/UCT Molecular Mycobacteriology Research Unit, Department of Pathology, University of Cape Town, Medical School Campus, Observatory 7925, South Africa; audrey.jordaan@uct.ac.za (A.J.); digby.warner@uct.ac.za (D.F.W.); 5 Institute of Infectious Disease and Molecular Medicine, University of Cape Town, Medical School Campus, Observatory 7925, South Africa; 6 Wellcome Centre for Infectious Diseases Research in Africa (CIDRI-Africa), University of Cape Town, Medical School Campus, Observatory 7925, South Africa; 7 Department of Medical Biochemistry, School of Laboratory Medicine and Medical Science, College of Health Sciences, University of KwaZulu Natal, Howard Campus, Durban 4001, South Africa; myburgr@ukzn.ac.za

**Keywords:** quinoline–urea–benzothiazole hybrids, antitubercular activity, minimum inhibitory concentration, HepG2 cell line, cytotoxicity, in silico ADME properties

## Abstract

A series of 25 new benzothiazole–urea–quinoline hybrid compounds were synthesized successfully via a three-step synthetic sequence involving an amidation coupling reaction as a critical step. The structures of the synthesized compounds were confirmed by routine spectroscopic tools (^1^H and ^13^C NMR and IR) and by mass spectrometry (HRMS). In vitro evaluation of these hybrid compounds for their antitubercular inhibitory activity against the *Mycobacterium tuberculosis* H_37_Rv pMSp12::GPF bioreporter strain was undertaken. Of the 25 tested compounds, 17 exhibited promising anti-TB activities of less than 62.5 µM (MIC_90_). Specifically, 13 compounds (**6b**, **6g**, **6i**–**j**, **6l**, **6o**–**p**, **6r**–**t**, and **6x**–**y**) showed promising activity with MIC_90_ values in the range of 1–10 µM, while compound **6u**, being the most active, exhibited sub-micromolar activity (0.968 µM) in the CAS assay. In addition, minimal cytotoxicity against the HepG2 cell line (cell viability above 75%) in 11 of the 17 compounds, at their respective MIC_90_ concentrations, was observed, with **6u** exhibiting 100% cell viability. The hybridization of the quinoline, urea, and benzothiazole scaffolds demonstrated a synergistic relationship because the activities of resultant hybrids were vastly improved compared to the individual entities. In silico ADME predictions showed that the majority of these compounds have drug-like properties and are less likely to potentially cause cardiotoxicity (QPlogHERG > −5). The results obtained in this study indicate that the majority of the synthesized compounds could serve as valuable starting points for future optimizations as new antimycobacterial agents.

## 1. Introduction

Tuberculosis (TB) is an infectious disease caused by *Mycobacterium tuberculosis* (*Mtb*) and it contributes to the high mortality rate, driven by infectious diseases, in most sub-Saharan African countries [1]. In 2019, the World Health Organization (WHO) reported over 10 million new TB cases and 1.2 million deaths. [2]. Furthermore, nearly one-third of the global population is estimated to be latently infected with TB. Although latent TB is not transmittable, at least 5–10% of people with latent TB will see it manifest into active TB at some point in their lifetime [2]. TB treatment with what are commonly referred to as first-line drugs usually lasts six months, but this may, in some cases, extend up to nine. Failure to complete this treatment course increases the risk of developing drug-resistant TB. Studies have shown that the administration of substandard drugs and/or sub-optimal drug doses also contributes to the manifestation and spread of drug-resistant TB [3]. There are three reported types of drug-resistant TB, namely, multidrug-resistant (MDR), extensively drug-resistant (XDR), and totally drug-resistant (TDR) TB [4]. MDR-TB is caused by a strain that is resistant to two at least of the first-line anti-TB drugs (i.e., isoniazid and rifampicin). In contrast, XDR-TB is resistant to several anti-TB drugs, any fluoroquinolone, and at least one injectable drug, while TDR-TB is a generic term for strains virtually resistant to all TB drugs [4,5]. Drug resistance is governed mainly by several mechanisms associated with (i) the thick, waxy, and hydrophobic cell wall envelope [6,7], (ii) the presence of drug modifying enzymes [8,9], and (iii) chromosomal or gene mutations [10,11], among others.

**Figure 1:**
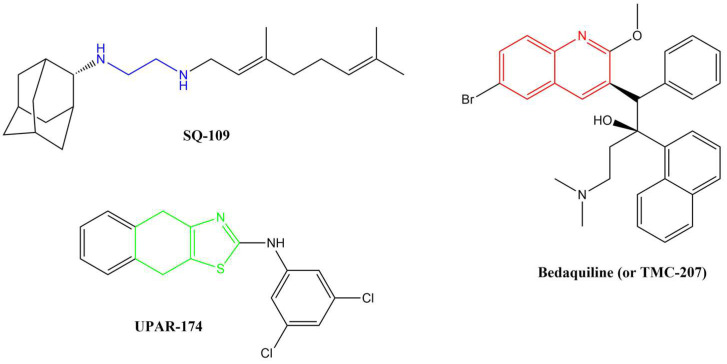
Breakthrough anti-TB compounds in various stages of development, approval, and use.

Several anti-TB drugs are at various phases of development, approval, and use, and three of the most recent breakthroughs in anti-TB research resulted in the discovery of SQ-109, UPAR-174, and bedaquiline (or TMC-207) (Figure 1).

SQ-109, a member of the 1,2-ethylenediamine-based anti-TB drugs, is currently undergoing phase II clinical trials and is active against MDR pulmonary TB [12,13,14]. SQ-109 is made up of a triage of moieties, namely, adamantane head, geranyl tail and 1,2-ethylenediamine linker, which are critically important for the drug’s anti-TB activity [14]. This drug functions by inhibiting the mycobacterial membrane protein large 3 (MmpL3), from the cytoplasm, which is involved in cell wall synthesis [15,16]. Other studies have reported that this drug also inhibits *Mtb’s* respiration by blocking menaquinone biosynthesis [17] and also acts by collapsing the pH gradient and cell wall membrane potential [18]. The major disadvantage of this compound, in terms of medicinal chemistry parameters, is its high lipophilicity (clog P = 6.82) [19]. Highly lipophilic compounds tend to have rapid metabolic turnover, low solubility and poor absorption [20]. In addition, these compounds tend to bind to off-target cells/tissues, leading to an increased risk of toxicity [21].

UPAR-174, a tricyclic 2-aminothiazole compound still in early development, possesses good anti-TB activity [22]. This compound is believed to exert its activity via potent efflux inhibitory properties similar to verapamil [22]. It also reduces the rate of drug efflux in both sensitive and drug-resistant *Mtb* [22]. This compound is highly lipophilic (clogP = 6.60) and has been reported to penetrate macrophages to facilitate the killing of *Mtb* when administered with isoniazid and rifampicin in small doses [23].

Bedaquiline is a diarylquinoline-based compound whose approval was accelerated by WHO and the US Food and Drug Administration (US-FDA). It is used with isoniazid, rifampicin, and pyrazinamide in a highly effective combination therapy used to treat MDR-TB [24]. It targets the *atpE*-encoded subunit c of the membrane-bound mycobacterial ATP synthase enzyme [25]. ATP synthase is responsible for transporting ions across the cell membrane and generating power for ATP synthesis. The quinoline core in this drug is critical for its activity. As effective as bedaquiline is, it also has several shortcomings. Firstly, its very high lipophilicity (clogP = 7.25) has been shown to result in its deposition in fatty tissues in the body [26]. This lipophilicity has also been directly linked to the inhibition of the cardiac potassium heERG channels, resulting in QTc interval prolongation, and hepatic toxicity [27]. Secondly, the recent reported emerging resistance toward bedaquiline among MDR-TB and XDR-TB clinical isolates is worrisome [28]. Drug resistance is by far the most significant challenge in TB chemotherapy. The mechanism of resistance to bedaquiline is thought to involve a direct mutation within the *atpE* gene.

**Figure 2:**
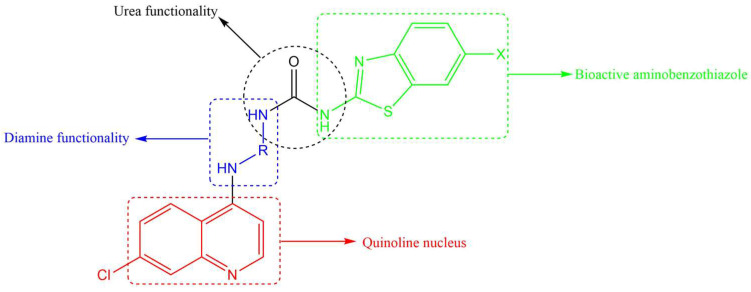
Components making up the desired quinoline–urea–benzothiazole hybrid compounds.

All three anti-TB drugs discussed above are highly lipophilic and various studies have shown that lipophilic compounds tend to exhibit high anti-TB activity due to their abilities to penetrate the hydrophobic cell wall. However, newer compounds of similar activity but with lower lipophilicity and improved solubility are desirable. Previously, we reported several 4-amino-7-chloroquinoline-based hybrid compounds that showed promising anti-TB activities, low lipophilicity, and improved solubility profiles [29,30]. In a continuation of our efforts to discover anti-infective agents, we report here on the synthesis, in vitro anti-TB activity, cytotoxicity studies, and in silico ADME profiling of new hybrid compounds. These compounds contain the chain length varying diamine functionality of SQ-109, the quinoline nucleus found in bedaquiline/TMC 207, and the aminothiazole (in our case, benzothiazole) found in UPAR-174 (Figure 2). The diamine and benzothiazole are linked via the urea moiety. Benzothiazoles and their derivatives are known to possess many important biological properties, such as antifungal [31], antibacterial [32], anticancer [33,34], and antituberculosis activities [35,36,37,38], among others.

In terms of anti-TB activity, benzothiazoles have been found to be effective in combatting MDR-TB [39]. They are thought to act by inhibiting the production of an enzyme used in the synthesis of biotin, an essential vitamin for the survival of *Mtb* [39,40]. Furthermore, this scaffold has also been shown to target another enzyme known as decaprenylphosphoryl-β-D-ribose 2′-epimerase, which is responsible for the synthesis of decaprenylphosphoryl arabinose [41,42,43]. Decaprenylphosphoryl arabinose plays a significant role in forming cell-wall arabinoses and inhibiting it leads to mycobacterial cell lysis and ultimately cell death. Other studies on benzothiazole-containing compounds, especially the 6-substituted benzothiazoles, resulted in efficacious compounds in vitro and in vivo [40]. A benzothiazole moiety is generally used in tandem with urea, and this combination has shown promising anti-TB activity [44]. Urea was selected as an active linker to covalently link both the benzothiazole and 4-aminoquinoline moieties and further enhance the anti-TB activity of the designed hybrid compounds. It is also anticipated that most of these designed hybrid compounds will be less lipophilic than SQ-109, UPAR-174, and bedaquiline, and will possess multimodal activity against *Mtb*.

## 2. Results and Discussion

### 2.1. Chemistry

**Scheme 1:**
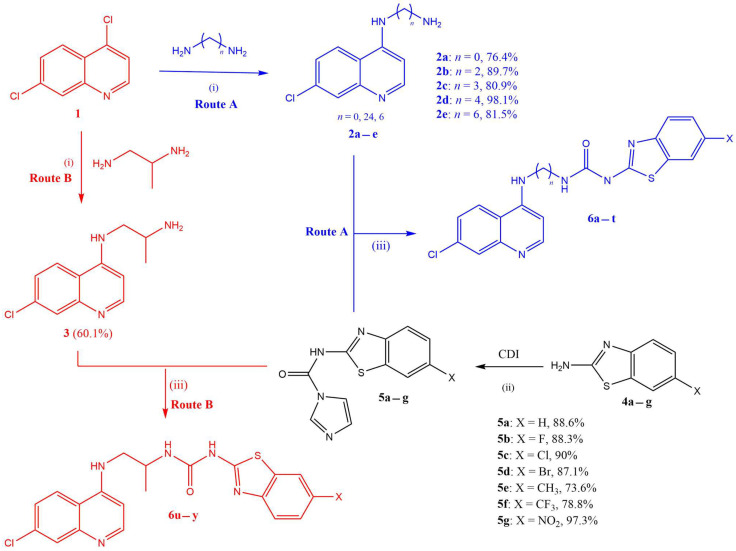
Reagents and conditions: (i) heat at 80 °C, 1 h (without stirring), then at 135 °C, 4 h (with stirring); (ii) CDI, dry DCM, reflux at 45 °C, 20 h; (iii) dry ACN, reflux at 85 °C, 24 h.

The synthesis of target quinoline–urea–benzothiazole hybrids (**6a**–**y**) followed a three-step synthetic protocol outlined in Scheme 1.

Briefly, the first step involved the synthesis of the intermediate 4-aminoquinoline diamines (**2a**–**e** and **3**: Routes A and B, respectively), in excellent yields (76–98%), from the reaction of 4,7-dichloroquinoline (**1**) and various excess diamines following a previously reported procedure [29]. In parallel and separately, various 2-amino-6-substituted benzothiazoles (**4a**–**g**) were converted to benzothiazole-1*H*-imidazol-1-carboxamide intermediates (**5a**–**g**), also in excellent yields (74–97%), using the 1,1′carbonyldiimidazoles (CDIs) in refluxing dry DCM according to modified methods reported in the literature [44]. It is worth noting that the CDI, in addition to facilitating the amide coupling reaction, also provides the urea functionality required in the target hybrids. Furthermore, the CDI must be in excess to avoid two benzothiazoles coupling to a single CDI moiety, which would be undesirable. The last step in the synthesis was the coupling of the 4-aminoquinoline diamines with benzothiazole-1*H*-imidazol-1-carboxamide intermediates (**5a**–**g**) in refluxing ACN to yield the desired compounds (**6a**–**y**) in excellent yields (Table 1). All the synthesized target compounds were confirmed by ^1^H and ^13^C NMR and by HRMS (see the Supplementary Materials).

### 2.2. In Vitro Antitubercular Activity Studies

The synthesized new hybrid compounds (**6a**–**y**) were evaluated for their potential in vitro antitubercular activity against the H_37_Rv strain over seven days of incubation in two slightly different media (7H9/CAS/Glu/Tx and 7H9/ADC/Glu/Tw) (Table 2). The two media shared the standard mycobacterial broth base (Middlebrook 7H9) and glucose (Glu) supplement. The only difference was that the former contained casitone (CAS) and tyloxapol (Tx) and the latter albumin–dextrose–catalase (ADC) and Tween-80 (Tx). ADC is considered a very useful indicator of potential protein binding (binding to serum) since it contains albumin (bovine albumin fraction V), while CAS is a source of amino acids for the bacteria [45]. Both Tx and Tw are useful surfactants in preventing mycobacterial clumping/agglomerating in liquid assays. The lowest concentration of a compound resulting in no visible mycobacterial growth is scored as the MIC_90_ (minimum inhibitory concentration required to inhibit 90% of the *Mtb* growth). Rifampicin (RIF) was used as a reference or positive control in the assays.

From the data, it is evident that activity is dependent on the structural features the compounds possess (i.e., the type of the diamine and its chain length, as well as the substituent on the benzothiazole moiety). In the hydrazine sub-series (**6a**–**d**), the CF_3_-containing compound **6b** was the only one showing excellent activity in both media. In contrast, **6a** (which had a H on the benzothiazole ring) only showed moderate activity (21.0 µM) in the CAS media. The F- and NO_2_-substituted compounds in this sub-series were devoid of activity, as seen by MIC_90_ values >125 µM in both media. In the ethylenediamine sub-series (**6e**–**h**), the Br-containing compound **6g** was the most active, displaying an excellent activity ranging from 8.89–14.90 µM in both media, while the F-substituted compound **6h** showed moderate activity of 62.5 µM in both media. In the propyldiamine sub-series (**6i**–**n**), generally, all the tested compounds showed excellent activity (<20 µM) in both media, with the Cl-substituted **6j** being the most active in the CAS assay (4.39 µM), while in the ADC assay the unsubstituted compound **6l** (9.45 µM) was the most active. Similar to the hydrazine series, the F-containing compound **6n** was devoid of any activity. In the butyldiamine sub-series (**6o**–**q**) (MIC_90_ in the range of 7.81–15.61 µM), the Br-containing compound (**6p**: 7.81 and 7.05 µM) was the most active, while the F-containing compound (**6q**) was not active at all. Interestingly, the F-substituted compound **6t**, in the hexyldiamine sub-series (**6r**–**t**), was the most active compound in comparison with the other sub-series tested. The last sub-series, the propane-1,2-diamine sub-series (**6u**–**y**), yielded the most active compounds (<10 µM) of them all. The CF_3_ substituted **6u** was the most active of all compounds tested with a sub-micromolar activity of (0.97 µM) in the CAS assay and 5.73 µM in the ADC assay. The fact that the activity decreased drastically (i.e., a six-fold decrease), as evidenced by the increasing MIC_90_ values, in the ADC media, could indicate potential protein binding to the albumin protein. The reference or positive control drug (RIF) was more active than all the tested compounds in both media.

In terms of intermolecular comparison across the six sub-series (Table 2), the propane-1,2-diamine sub-series provided the most active compound (**6u**). Notably, all the tested CF_3_-containing **6b**, **6i**, and **6u** appear to be the consistently active compounds in both media in their respected sub-series. This observation is not surprising because various studies have confirmed the essential biological role attributed to the CF_3_ substituent by virtue of containing fluorides [47]. The CF_3_-containing propane-1,2-diamine compound **6u** could serve as an interesting starting point in the next round of structure–activity relationship (SAR) studies to discover the next generation of these designed hybrids. Lipophilicity did not appear to play any prominent role in the activities of these compounds since there was no observable trend in this property based on *Mtb* activities.

In assessing whether the hybridization strategy offered any advantage or not, the individual intermediates (**2e**, **4b** (including **5b**) and urea) making up compound **6t** were tested separately and in combination (weight/weight), and their activities were compared to that of **6t**. All intermediates making up compound **6t** and their combination were found to be largely inactive (>125 µM) compared to the hybrid, which had MIC_90_ values ranging from 6.97–31.25 µM. Secondly, the combination was also not active, conclusively suggesting that the hybridization was advantageous compared to the individual entities (and their combination) making up the hybrid. Furthermore, this could also indicate that the intermediates making up the hybrid have a synergistic relationship, as seen by the improved activity when hybridized.

### 2.3. In Vitro Cytotoxicity Studies

**Figure 3:**
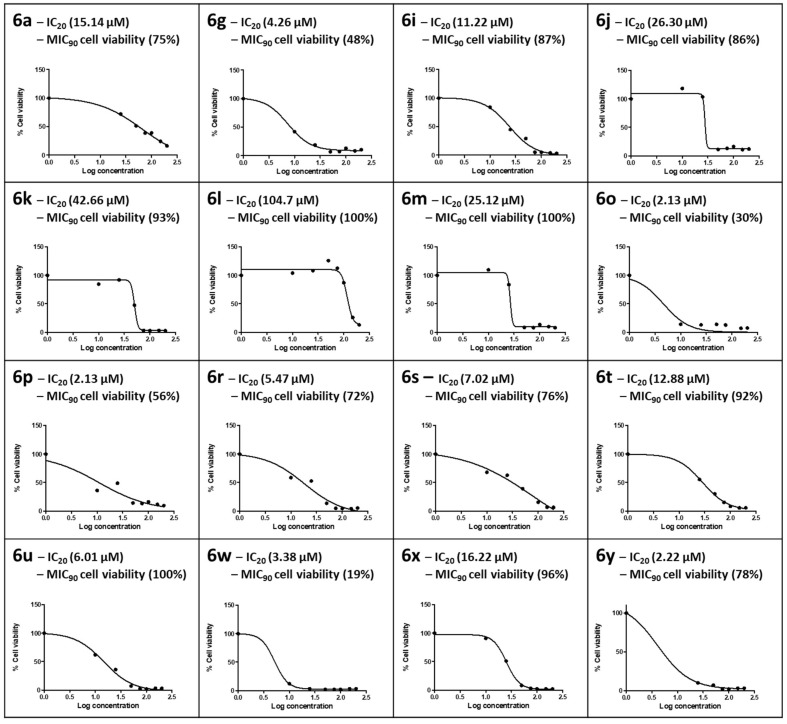
The dose–response curves for HepG2 cells exposed to 0–200 µM concentrations of the selected number of synthesized compounds. The 20% inhibitory concentration (IC_20_) and cell viability at the 90% *Mtb* inhibitory concentration for CAS (MIC_90_) were extrapolated from the dose–response curve.

In vitro cytotoxicity of the synthesized compounds was determined using the MTT assay. This cell viability assay quantifies the enzymatic conversion of the yellow tetrazolium salt into purple formazan crystals by healthy, living mammalian cells. The formazan crystals produced are directly proportional to the number of viable cells [48]. The synthesized compounds showed variable cytotoxic responses for HepG2 cells at the concentrations tested (0–200 µM), with cell viability ranging from 10% to 100% (Figure 3). The ISO norm requires >75% cell viability for biomedical products [49]. In this study, an IC_20_ where HepG2 cells retained 80% viability was applied for the compound to be deemed safe at the MIC_90_ obtained in the CAS assay; only those with MIC_90_ values <60 µM were tested for cytotoxicity. For compound **6a**, part of the hydrazine sub-series containing only H on the benzothiazole ring, the IC_20_ of 15.14 µM in HepG2 cells was lower than its MIC_90_ (21.0 µM), where HepG2 cells at this concentration retained 75% cell viability in accordance with the ISO norm.

Compound **6g** in the ethylenediamine sub-series was cytotoxic to HepG2 cells; HepG2 cell viability was 48% at the MIC_90_ for **6g**, and an IC_20_ of 7.47 µM was calculated. In the propyldiamine sub-series, only **6i** exhibited a dose-dependent toxic response, with **6j**–**6m** showing a sharp drop in cell viability at concentrations >25 µM. The IC_20_s of HepG2 cells for this series were all above the respective MIC_90_s. Moreover, cell viability at the MIC_90_ ranged from 86% to 100% in accordance with the ISO norm for biomedical products, and **6l** yielded the best safety profile over a more comprehensive concentration range, with an IC_20_ of 104.7 µM and 100% cell viability at the MIC_90_. Both **6o** (MIC_90_ 7.43 µM) and **6p** (MIC_90_ 7.81 µM) from the butyldiamine sub-series were cytotoxic to HepG2 cells and did not conform to the ISO norm, retaining only 30% and 56% cell viability at the respective MIC_90_s. Although the IC_20_s for **6o** and **6p** were both 2.13 µM in HepG2 cells, it is interesting to note a protective effect in the Br-containing **6p** at the MIC_90_. It is also interesting to note that the F-substituted compound **6t** in the hexyldiamine sub-series exhibited the highest cell viability at the MIC_90_ (92%) compared to **6r** (72%) and **6s** (76%). Compound **6u** (CF3 substituted), the most active of all the synthesized compounds of the propane-1,2-diamine sub-series, yielded 100% cell viability at its MIC_90_ (0.97 µM), but **6w** was extremely cytotoxic, with only 19% cell viability at its MIC_90_. Both **6x** and **6y** conformed to the ISO standard, with MIC_90_ HepG2 cell viability levels of 96% and 78%, respectively.

### 2.4. In Silico ADME Profiling of the Synthesized Compounds Using QikProp

To further validate the potential albumin protein binding observed in the 7H9/ADC/Glu/Tw media data above and to understand the possible effects of the high lipophilicity (clogP > 4), all the synthesized compounds were profiled in silico using a Schrodinger QikProp tool [50] for their ADME properties (Table 3). QikProp is a very useful tool for comparing any new compound’s potential ADME properties against known drugs (>95%) used to develop the tool. Studies have shown that the primary reasons for the high attrition rate of drug candidates during clinical trials are poor ADME and unfavourable drug-likeness properties [51,52]. Prior to the prediction, all the structures of the synthesized compounds were neutralized, and the program was run on the normal mode. Generally, all the synthesized compounds showed high predicted human oral absorption percentage (>80%) and the majority fulfilled Lipinski’s drug-like properties by having two (compound **6s** only) or fewer violations (compounds **6p**, **6r**, **6t**, and **6u**—one violation each) of the rule of five (less than four violations) [53]. The predicted brain–blood partition coefficient (QPlogBB) was also within the acceptable range. Surprisingly, the predicted binding to human serum albumin (QPlogKhsa) was within the acceptable range, contrary to the experimentally observed potential protein binding in the ADC *Mtb* assay. Encouragingly, all the synthesized compounds were predicted to have IC_50_ values for the blockage of HERG K^+^ channels (QlopHERG) greater than −5. This is indicative of compounds that are devoid of any potential to inhibit the cardiac potassium HERG K^+^ channels and less likely to cause any cardiotoxicity.

## 3. Materials and Methods

### 3.1. Chemistry

All chemical reagents were purchased from Sigma-Aldrich (Modderfontein South Africa), Merck (Modderfontein, South Africa), Alfa Aesar (Kyalami, South Africa), or Thembane chemicals (Johannesburg, South Africa) and were used without further purification. Throughout the study, 1.1′Carbonyldiimidazole was kept in a desiccator. All solvents were purchased from Sigma-Aldrich and were HPLC or analytical grade. Deionized water was used throughout the study. Nuclear magnetic resonance (NMR) spectroscopic studies were performed using a Bruker Fourier 400 MHz and a Varian Gemini 400 spectrometer at 400 MHz and 100 MHz for the ^1^H NMR and ^13^C NMR nuclei, respectively, and all chemical shifts were referenced using solvent signals. Tetramethylsilane (TMS) was used as the internal standard, with chemical shifts reported in ppm and coupling constants (*J*) reported in Hertz (Hz). Reference peaks for deuterated chloroform (CDCl_3_) were 7.26 ppm for ^1^H NMR and 77.2 ppm for ^13^C NMR; for dimethyl sulfoxide (DMSO-*d*_6_), 2.50 ppm for ^1^H NMR and 39.5 ppm for ^13^C NMR; and for deuterated methanol (Meth-*d*), 4.87 ppm and 3.31 ppm for ^1^H NMR and 49.1 ppm for ^13^C NMR. Melting point studies were conducted on Stuart Scientific melting point apparatus SMP3. Fourier-transform infrared (FT-IR) spectroscopy studies were performed using a PerkinElmer FT-IR spectrometer. High-resolution mass spectrometry (HRMS) data, used to predict the present elements and to confirm the molecular weights of the synthesized compounds, were obtained with Waters Synapt G2, ESI probe, ESI Pos, Cone Voltage 15 V HRMS.

### 3.2. General Procedure for the Synthesis of Hybrid Compounds ***6a**–**y***

*N-(Benzothiazole-2-yl)-1H-imidazole-1-carboxamides* (**5a**–**g**) (1.1 mmol) with the respective *N*-(7-Chloro-4-quinolinyl)-diamines (**2a**–**e** and **3**) (1.3 mmol) in HPLC grade acetonitrile (20 mL) were refluxed in an oil bath at 85 °C for 24 h. After reaction completion, the reaction mixture was cooled to room temperature and quenched with 1 M HCl solution (30 mL). The precipitated product was filtered and washed thoroughly with cold deionized water and air dried to obtain the desired target compounds (**6a**–**y**) in good to excellent yields and at an acceptable purity.

*N-(Benzo[d]thiazol-2yl)-2-(7-chloroquinolin-4-yl)hydrazine-1-carboxamide* (**6a**). Peach powder; yield: 85.52%; m.p. 198–202 °C; IR (cm^−1^): 3292, 1534 (NH), 1649 (C=C), 1750 (C=O), 1350 (C-N); ^1^H NMR (400 MHz, DMSO-*d*_6_, ppm); ^1^H NMR (400 MHz, DMSO-*d*_6_, ppm): δ_H_ 10.13 (NH, brs, 1H), 8.63 (H2 and H1′, dd, *J* 13.6, 8.0 Hz, 2H), 8.17 (H4′, d, *J* 2.1 Hz, 1H), 7.88–7.80 (H8 and H5, m, 2H), 7.58 (H6, d, *J* 8.1 Hz, 1H), 7.36 (H2′, ddd, *J* 8.2, 7.3, 1.3 Hz, 1H), 7.21 (H3′, ddd, *J* 8.3, 7.3, 1.2 Hz, 1H), 6.99 (H3, d, *J* 6.9 Hz, 1H); ^13^C NMR (101 MHz, DMSO-*d*_6_, ppm): δ_C_ 157.6, 144.3, 138.9, 128.0, 127.6, 127.1, 124.7, 123.1, 119.7, 114.3, 99.6. HRMS (TOF MS ES^+^): found M + 1: 370.0524, C_17_H_13_N_5_OSCl requires MH^+^, 370.0529.

*2-(7-Chloroquinolin-4-yl)-N-(6-(trifluoromethyl)benzo[d]thiazol-2-yl)hydrazine-1-carboxamide* (**6b**). Yellow powder; yield: 84.81%; m.p. 243–245 °C; IR (cm^−1^): 3226, 1543 (NH), 1662 (C=C), 1655 (C=O), 1363 (C-N); ^1^H NMR (400 MHz, DMSO-*d*_6_, ppm): δ_H_ 10.26 (NH, brs, 1H), 8.67 (H2 and H1′, dd, *J* 16.6, 8.0 Hz, 2H), 8.36 (H4′, s, 1H), 8.16 (H8, d, *J* 2.1 Hz, 1H), 7.84 (H5, dd, *J* 2.2, 0.8 Hz, 1H), 7.76 (H2′, d, *J* 8.5 Hz, 1H), 7.67 (H6, dd, *J* 8.5, 1.9 Hz, 1H), 7.02 (H3, d, *J* 6.9 Hz, 1H); ^13^C NMR (101 MHz, DMSO-*d*_6_, ppm): δ_C_ 157.5, 144.4, 138.9, 132.1, 128.8, 128.02, 126.4, 125.3, 124.9, 124.1, 123.45, 123.3, 121.9, 120.1, 119.7, 114.4, 99.6. HRMS (TOF MS ES^+^): found M + 1: 438.0402, C_18_H_12_N_5_OF_3_SCl requires MH^+^, 438.0403.

*2-(7-Chloroquinolin-4-yl)-N-(6-fluorobenzo[d]thiazol-2-yl)hydrazine-1-carboxamide* (**6c**). Yellow oxide powder; yield: 86.11%; m.p. 240–242 °C; IR (cm^−1^): 3362, 1543 (NH), 1648 (C=C), 1643 (C=O), 1377 (C-N); ^1^H NMR (400 MHz, DMSO-*d*_6_, ppm): δ_H_ 10.32 (NH, brs, 1H), 8.74 (H-2, d, *J* 6.6 Hz 1H), 8.69 (H1′, d, *J* 10.9, 1H), 8.18 (H4′, s, 1H), 7.78 (H5 and H8, m, 2H), 7.59 (NH, s, 1H), 7.37 (H2′, *J* 6.9 Hz, 1H), 7.27 (H6, d, *J* 7.1 Hz, 1H), 6.73 (H3, d, *J* 3.2 Hz 1H); ^13^C NMR (101 MHz, DMSO-*d*_6_, ppm): δ_C_ 158.0, 146.7, 138.1, 131.1, 128.1, 127.4, 127.2, 127.0, 127.3, 126.2 (2 × C), 125.4, 124.1, 123.9, 120.4, 119.90, 99.9. HRMS (TOF MS ES^+^): found M + 1: 388.0434, C_17_H_12_N_5_OFSCl requires MH^+^, 388.0435.

*2-(7-Chloroquinolin-4-yl)-N-(6-nitrobenzo[d]thiazol-2-yl)hydrazine-1-carboxamide* (**6d**). Yellow powder; yield: 78.75%; m.p. 239–241 °C; IR (cm^−1^): 3222, 1534 (NH), 1658 (C=C), 1653 (C=O), 1393 (C-N); ^1^H NMR (400 MHz, DMSO-*d*_6_, ppm): δ_H_ 8.79–8.58 (2 × NH, s, 2H), 8.59–8.45 (H2 and H1′, m, 2H), 8.20 (H5, dd, *J* 6.6 and 1.4 Hz, 1H), 8.10 (H-8, d, *J* 1.6 Hz, 1H), 7.69 (H4′, d, *J* 1.3 Hz, 1H), 7.40 (H2′ and H6, d, *J* 9.0 Hz, 2H), 7.12 (H3, m, 1H); ^13^C NMR (101 MHz, DMSO-*d*_6_, ppm): δ_C_ 172.2, 157.7, 156.1, 143.2, 142.5, 141.4, 138.8, 138.2, 131.5, 126.9, 125.9, 122.6, 119.3, 118.3, 117.0, 114.1, 98.6. HRMS (TOF MS ES^−^): found M-1: 413.0208, C_17_H_10_N_6_O_3_SCl requires M-H, 413.224.

*1-(2-((7-Chloroquinolin-4-yl)amino)ethyl)-3-(6-(trifluoromethyl)benzo[d]thiazol-2-yl)urea* (**6e**). Cream white powder; yield: 64.90%; m.p. 249–251 °C; IR (cm^−1^): 3281, 1533 (NH), 1614 (C=C), 1758 (C=O), 1358 (C-N); ^1^H NMR (400 MHz, DMSO-*d*_6_, ppm): δ_H_ 9.60 (NH, brt, *J* 5.5 Hz, 1H), 8.64 (H2, d, *J* 9.2 Hz, 1H), 8.60 (H1′, d, *J* 7.1 Hz, 1H), 8.16 (H4′, s, 1H), 7.99 (NH, s, 1H), 7.79 (H5 and H8, dd, *J* 7.1, 2.3 Hz, 2H), 7.55–7.32 (H2′ and NH, m, 2H), 7.24 (H6, m, 1H), 6.83 (H3, d, *J* 9.2 Hz, 1H), 3.62 (H10a, m, 1H), 3.22 (H10b, m, 1H), 199-178 (2H, M, H9); ^13^C NMR (101 MHz, DMSO-*d*_6_, ppm): δ_C_ 163.2, 158.3, 156.9, 143.2, 138.9, 138.4, 132.44, 128.2, 127.5, 127.0, 126.4, 123.3, 123.1, 120.3, 119.8, 119.4, 116.8, 99.1, 43.5, 38.3. HRMS (TOF MS ES^+^): found M + 1: 466.0719, C_20_H_16_N_5_OF_3_SCl requires MH^+^, 466.0716.

*1-(6-Chlorobenzo[d]thiazol-2-yl)-3-(2-((7-chloroquinolin-4-yl)amino)ethyl)urea* (**6f**). As a dark cream solid; yield: 83.23%; m.p. 244–245 °C; FT-IR (*v_max_*/cm^−1^): *v* (C=O) 1535.72, *v* (NH) 2938.94; 3238.94, 3354.12, ^1^H NMR (400 MHz, DMSO-*d*_6_, ppm): δ_H_ 11.24–10.99 (2 × NH, 2 × brs, 2H), 9.28 (H2, d, *J* 8.90 Hz, 1H), 8.56 (H1′, m, 2H), 8.09 (H4′ and H8, m, 2H), 7.89 (H5, m, 1H), 7.73 (NH and H2′, m, 2H), 7.23 (H6, d, *J* 8.7 Hz, 1H), 6.99 (H3, d, *J* 9.1 Hz, 1H), 3.77 (H10, m, 2H), 3.55 (H9, m, 2H); ^13^C NMR (101 MHz, DMSO-*d*_6_, ppm): δ_c_ 162.9, 156.7, 155.2, 154.9, 148.3, 144.3, 140.1, 138.7, 133.2, 127.9, 127.3, 127.0, 121.2, 120.3, 119.9, 116.5, 98.4, 44.3, 37.8. HRMS (TOF MS ES^+^): found M + 1: 432.0451, C_19_H_16_N_5_OSCl_2_ requires MH^+^, 432.0453.

*1-(6-Bromobenzo[d]thiazol-2-yl)-3-(2-((7-chloroquinolin-4-yl)amino)ethyl)urea* (**6g**). Light brown solid; yield: 78.41%; m.p. 240–242 °C; FT-IR (*v_max_*/cm^−1^): *v* (C=O) 1667.12, *v* (NH) 2936.09; 3340.35, 3413.64, ^1^H NMR (400 MHz, DMSO-*d*_6_, ppm): δ_H_ 8.49 (H2, d, *J* 7.8 Hz, 1H), 8.25 (H1′, dd, *J* 7.1, 2.1 Hz, 1H), 8.16 (H4′, d, *J* 1.91 Hz, 1H), 7.82 (H8, s, 1H), 7.55 (H2′ and H5, 2 × d, *J* 7.0 and 6.5 Hz, 2H), 7.49 (H6 and NH, m, 2H), 7.15 (NH, brs, 1H), 6.97 (NH, brs, 1H), 6.52 (H3, d, *J* 7.8 Hz, 1H), 3.49 (H10, m, 2H), 3.35 (H9, m, 2H); ^13^C NMR (101 MHz; DMSO-*d*_6_, ppm): δ_c_ 163.0, 154.9, 154.2, 153.6, 151.7, 148.3, 134.9, 134.0, 129.7, 127.3, 124.9, 123.7, 123.4, 123.1, 122.3, 118.6, 114.7, 98.6, 44.8, 37.6. HRMS (TOF MS ES^+^): found M + 1: 475.9948, C_20_H_16_N_5_OBrSCl requires MH^+^, 475.9947.

*1-(6-Fluorobenzo[d]thiazol-2-yl)-3-(2-((7-chloroquinolin-4-yl)amino)ethyl)urea* (**6h**). Pink solid; yield: 75.23%; m.p. 230–232 °C; FT-IR (*v_max_*/cm^−1^): *v* (C=O) 1649.32, *v* (NH) 3261.71; 3354.28, 3399.65; ^1^H NMR (400 MHz, DMSO-*d*_6_, ppm) δ_H_ 11.05-10.55 (2 × NH, 2 × brs, 2H), 8.52 (H4′, dd, *J* 6.8, 1.4 Hz, 1H), 7.89 (H1′ and H2, m, 2H), 7.69 (H5 and H8, m, 2H), 7.32 (2H, H2′ and H6, m, 2H), 6.79 (H3, d, *J* 6.9 Hz, 1H), 3.73 (H10a, m, 1H), 3.51 (H10b, m, 1H), 3.21 (H9, m, 2H); ^13^C NMR (101 MHz; DMSO-*d*_6_, ppm): δ_c_ 161.9, 159.7, 157.8, 156.3, 155.0, 146.3, 143.2, 138.2, 137.5, 132.9–132.1 (*C3′ coupling to F atom*), 128.1, 127.3, 121.9, 119.4, 117.3, 113.9–113.0 (*C2′ coupling to F atom*), 108.3–107.6 (*C4′ coupling to F atom*), 98.3, 44.9, 32.8. HRMS (TOF MS ES^+^): found M + 1: 416.0740, C_20_H_16_N_5_OFSCl requires MH^+^, 416.0748.

*1-(3-((7-Chloroquinolin-4-yl)amino)propyl)-3-(6-(trifluoromethyl)benzo[d]thiazol-2-yl)urea* (**6i**). Yellow powder; yield: 83.42%; m.p. 198–200 °C; IR (cm^−1^): 3255, 1532 (NH), 1691 (C=C), 152 (C=O), 1343 (C-N); ^1^H NMR (400 MHz, DMSO-*d*_6_, ppm): δ_H_ 9.42 (NH, brs, 1H), 8.61 (H2, d, *J* 7.9 Hz, 1H), 8.55 (H1′, d, *J* 6.8 Hz, 1H), 8.32 (H4′, s, 1H), 7.83 (H8, s, 1H), 7.75 (H5, d, *J* 7.2 Hz, 1H), 7.51 (H2′ and NH, m, 1H), 7.24 (NH, t, *J* 7.2 Hz, 1H), 6.89 (H6, d, *J* 6.9 Hz, 1H), 6.89 (H3, d, *J* 7.8 Hz, 1H), 3.58 (H11, m, 2H), 3.20 (H9a, m, 1H and *H9b overlaps with water peak), 1.90 (H10, m, 2H); ^13^C NMR (101 MHz, DMSO-*d*_6_, ppm): δ_c_ 207.1, 162.7, 153.7, 152.3, 149.5, 136.38, 134.3, 129.5, 128.3 (2 × C), 127.8 (2 × C), 125.1, 118.4, 99.5, 59.3, 44.5, 33.4. HRMS (TOF MS ES^+^): found M + 1: 480.0167, C_21_H_18_N_5_OSF_3_Cl requires MH^+^, 480.0173.

*1-(6-Chlorobenzo[d]thiazol-2-yl)-3-(3-((7-chloroquinolin-4-yl)amino)propyl)urea* (**6j**). Yellow-brown solid; yield: 76.82%; m.p. 286–288 °C; IR (cm^−1^): 3301, 1564 (NH), 1664 (C=C), 1618 (C=O), 1360 (C-N); ^1^H NMR (400 MHz, DMSO-*d*_6_, ppm): δ_H_ 11.25 (NH, s, 1H), 9.51 (NH, brs, 1H), 8.63 (H2 and H1′, m, 2H), 8.41 (H4′, s, 1H), 8.01 (H8, s, 1H), 7.79 (H2′ and NH, m, 2H), 7.64 (H5, d, *J* 6.9 Hz, 1H), 7.33 (H6, d, *J* 6.8 Hz, 1H), 7.12 (H3, d, *J* 7.4 Hz, 1H), 4.12 (H11a, m, 1H and *H11b overlaps with water peak), 3.71–3.55 (H9, m, 2H), 1.33 (H10, q, *J* 9.1 Hz, 2H); ^13^C NMR (101 MHz, DMSO-*d*_6_, ppm): δ_c_ 163.9, 158.8–158.0 (2 × C), 144.6, 138.9–138.5 (2 × C), 137.6–137.3 (2 × C), 128.3–127.9 (2 × C), 126.5–126.0 (2 × C), 124.5, 119.7–119.6 (2 × C), 117.3, 99.8, 48.6, 45.2, 18.7. HRMS (TOF MS ES^+^): found M + 1: 446.0607, C_20_H_18_N_5_OSCl_2_ requires MH^+^, 446.0609.

*1-(3-((7-Chloroquinolin-4-yl)amino)propyl)-3-(6-methylbenzo[d]thiazol-2-yl)urea* (**6k**). Faint pink powder; yield: 55.94%; m.p. 234–239 °C; IR (cm^−1^): 3266, 1543 (NH), 1660 (C=C), 1625 (C=O), 1373 (C-N); ^1^H NMR (400 MHz, DMSO-*d*_6_, ppm): δ_H_ 9.51 (2 × NH, m, 2H), 8.81 (H2, d, *J* 9.0 Hz, 1H), 8.51 (H1′, d, *J* 7.0 Hz, 1H), 7.96 (H2′, d, *J* 7.0 Hz, 1H), 7.75 (H4′, d, *J* 2.1 Hz, 1H), 7.68 (H8, s, 1H), 7.41 (H5, d, *J* 8.2 Hz, 1H), 7.21 (H6, d, *J* 8.2 Hz, 1H), 6.85 (H3, d, *J* 9.0 Hz, 1H), 3.53 (H11a, m, 1H and *H11b and H9 overlaps with water peak), 2.31 (CH_3_, s, 3H), 1.83 (H10, m, 2H); ^13^C NMR (101 MHz, DMSO-*d*_6_, ppm): δ_c_ 160.9, 157.1, 154.9, 143.2, 139.7 (2 × C), 139.4, 132.7, 132.1, 127.4 (2 × C), 126.5, 122.1 (2 × C), 119.4, 116.8, 99.8, 41.7, 37.5, 28.9, 20.1. HRMS (TOF MS ES^+^): found M + 1: 426.1155, C_21_H_21_N_5_OSCl requires MH^+^, 426.1155.

*1-(3-((7-Chloroquinolin-4-yl)amino)propyl)-3-(benzo[d]thiazol-2-yl)urea* (**6l**). Grey powder; yield: 59.31%; m.p. 285–288 °C; ^1^H NMR (400 MHz, DMSO-*d*_6_, ppm): δ_H_ 9.32 (NH, brs, 1H), 8.64 (H2, d, *J* 8.1 Hz, 1H), 8.53 (H1′, d, *J* 9.1 Hz, 1H), 8.40 (H8, s, 1H), 7.82 (H4′ and H5, m, 2H), 7.69 (H3′ and H2′, m, 2H), 7.43 (H6, d, *J* 8.1 Hz, 1H), 6.88 (H3, d, *J* 8.0 Hz, 1H), 3.61 (H11, m, 2H), 3.31 (H9a, m, 1H and *H9b overlaps with water peak), 1.88 (H10, m, 2H); ^13^C NMR (101 MHz, DMSO-*d*_6_, ppm): δ_c_ 154.6 (2 × C), 151.1, 150.2, 134.7 (2 × C), 127.9–127.5 (4 × C), 124.6–124.2 (3 × C), 118.9 (2 × C), 99.8, 52.5, 45.1, 23.1. HRMS (TOF MS ES^+^): found M + 1: 412.1002, C_20_H_19_N_5_OSCl requires MH^+^, 412.0999.

*1-(6-Bromobenzo[d]thiazol-2-yl)-3-(3-((7-chloroquinolin-4-yl)amino)propyl)urea* (**6m**). Pale yellow solid; yield: 60.20%; m.p. 254–257 °C; ^1^H NMR (400 MHz, DMSO-*d*_6_, ppm): δ_H_ 8.40 (H2, d, *J* 5.3 Hz, 1H), 8.25 (H1′, m, 1H), 8.12 (H4′ and NH, s, 2H), 7.78 (H8, d, *J* 1.4 Hz, 1H), 7.54 (H5, m, 1H), 7.48 (H2′, m, 1H), 7.34 (H6, t, *J* 5.0 Hz, 1H), 6.95 (2 × NH, 2 × brs, 2H), 6.49 (H3, d, *J* 5.4 Hz, 1H), 3.23 (H11 and H9, m, 4H), 1.91 (H10a, m, 1H), 1.68 (H10b, m, 1H); ^13^C NMR (101 MHz, DMSO-*d*_6_, ppm): δ_c_ 162.9, 154.6, 152.3, 150.1, 148.3, 147.0, 135.2, 128.3, 126.4, 124.3, 123.8, 122.4, 118.9 (2 × C), 114.6, 99.8, 41.3, 38.6, 31.3, 27.3: HRMS (TOF MS ES^+^): found M + 1: 490.0110, C_20_H_18_N_5_OBrSCl requires MH^+^, 490.0104.

*1-(6-Fluorobenzo[d]thiazol-2-yl)-3-(3-((7-chloroquinolin-4-yl)amino)propyl)urea* (**6n**). Light brown solid; yield: 78.50%; m.p. 241–243 °C; ^1^H NMR (400 MHz, DMSO-*d*_6_, ppm): δ_H_ 8.42 (H1′, m, 1H), 8.26 (H2, d, *J* 8.9 Hz, 1H), 8.07 (H4′, d, *J* 1.7 Hz, 1H), 7.79 (H8, d, *J* 4.3 Hz, 1H), 7.52 (H5, d, *J* 10.6 Hz, 2H), 7.45 (H2′ and H6, d, *J* 8.6 Hz, 2H), 6.92 (NH, t, *J* 5.0 Hz, 1H), 6.55 (H3, d, *J* 8.9 Hz, 1H), 3.38 (H11a, m, 1H), 3.29 (H11b, m, 1H), 3.20 (H9, m, 2H), 1.92 (H10a, m, 1H), 1.67 (H10b, m, 1H); ^13^C NMR (101 MHz, DMSO-*d*_6_, ppm): δ_c_ 163.9, 153.2, 151.1, 149.9, 148.6, 148.0, 137.9, 129.3, 127.2, 123.8, 123.3, 122.5, 119.9 (2 × C), 116.4, 98.5, 43.0, 37.9, 30.2, 26.9: HRMS (TOF MS ES^+^): found M + 1: 430.0896, C_20_H_18_N_5_OFSCl requires MH^+^, 430.0905.

*1-(6-Chlorobenzo[d]thiazol-2-yl)-3-(4-((7-chloroquinolin-4-yl)amino)butyl)urea* (**6o**). Yellow-brown solid; yield: 83.04%; m.p. 189.8–191.3 °C; FT-IR (*v_max_*/cm^−1^): *v* (C=O) 1538.60*v* (NH) 2964.7, 3339.73, 3389.02; ^1^H NMR (400 MHz, CH_3_OH-*d*4, ppm): δ_H_ 8.39 (H2 and H1′, m, 2H), 7.80 (H4′ and H5, d, *J* 8.8 Hz, 2H), 7.69 (H8, d, *J* 3.3 Hz, 1H), 7.51 (H2′, d, *J* 7.2 Hz, 1H), 7.33 (H6, d, *J* 8.6 Hz, 1H), 6.87 (H3, d, *J* 6.5 Hz, 1H), 3.62 (H12, m, 2H), 3.36 (H9, m, 2H), 1.83 (H11, m, 2H), 1.75 (H10, m, 2H); ^13^C NMR (101 MHz; DMSO-*d*_6_, ppm): δ_c_ 163.1, 156.2, 154.8, 148.3, 144.6, 139.7, 138.3, 133.7, 127.5, 127.0, 126.7, 122.4, 122.1, 120.3, 116.9, 98.3, 43.6, 41.3 (*overlaps with solvent peak), 27.3, 21.2. HRMS (TOF MS ES^+^): found M + 1: 460.0763, C_21_H_20_N_5_OSCl_2_ requires MH^+^, 460.0766.

*1-(6-Bromobenzo[d]thiazol-2-yl)-3-(4-((7-chloroquinolin-4-yl)amino)butyl)urea)* (**6p**). Dark yellow solid; yield: 82.34% m.p. 192–194 °C; ^1^H NMR (400 MHz, DMSO-*d*_6_, ppm): δ_H_ 10.93 (NH, brs, 1H), 8.49 (H2, d, *J* 7.1 Hz, 1H), 8.35 (H1′, d, *J* 8.1 Hz, 1H), 8.19 (H4′, s, 1H), 8.19 (H8 and NH, s, 2H), 7.50–7.42 (H2′, H5 and H6, m, 3H), 6.92 (NH, brs, 1H), 6.63 (H3, d, *J* 7.0 Hz, 1H), 3.25 (H12 and H9, m, 4H), 1.71 (H11, m, 2H), 1.65 (H10, m, 2H); ^13^C NMR (101 MHz; DMSO-*d*_6_, ppm): δ_c_ 162.1, 154.3, 152.7, 151.9, 149.3, 135.3, 134.8, 129.3, 126.7, 125.9, 125.3, 124.7, 122.4, 118.1, 114.9, 99.6, 43.1, 42.2, 27.5, 26.2. HRMS (TOF MS ES^+^): found M + 1: 504.0263, C_21_H_20_N_5_OBrSCl requires MH^+^, 540.0260.

*1-(6-Fluorobenzo[d]thiazol-2-yl)-3-(4-((7-chloroquinolin-4-yl)amino)butyl)urea* (**6q**). Pale yellow solid; yield: 96.05% m.p. 256–259 °C; ^1^H NMR (400 MHz, DMSO-*d*_6_, ppm): δ_H_ 8.39 (H2, d, *J* 7.2 Hz, 1H), 8.25 (H1′, d, *J* 9.6 Hz, 1H), 8.15 (H4′, d, *J* 2.2 Hz, 1H), 7.81 (H8, d, *J* 3.2 Hz, 1H), 7.72 (NH, brs, 1H), 7.51 (H5, d, *J* 9.8 Hz, 1H), 7.42 (H2′ and H6, m, 2H), 6.81 (2 × NH, brs, 2H), 6.61 (H3, d, *J* 7.0 Hz, 1H), 3.36 (H12, m, 2H), 3.21 (H12, m, 2H), 1.71 (H11, m, 2H), 1.62 (H10, m, 2H); ^13^C NMR (101 MHz; DMSO-*d*_6_, ppm): δ_c_ 167.8, 162.1, 160.3, 157.5, 147.5, 133.9–132.9 (*C3′ coupling to F atom*), 125.7, 125.0, 122.7, 119.3, 114.9–114.7 (*C2′ coupling to F atom*), 113.9, 113.2, 109.9, 109.7–109.2 (*C4′ coupling to F atom*), 99.7, 44.6, 42.5, 22.5, 20.7. HRMS (TOF MS ES^+^): found M + 1: 444.1063, C_21_H_20_N_5_OFSCl requires MH^+^, 444.1061.

*1-(6-Chlorobenzo[d]thiazol-2-yl)-3-(6-((7-chloroquinolin-4-yl)amino)hexyl)urea* (**6r**). Yellow-brown solid; yield: 79.7%; m.p. 239–241 °C; FT-IR (*v_max_*/cm^−1^): *v* (C=O) 1666.63, *v* (NH) 3076.20, 3340.97, 3412.01; ^1^H NMR (400 MHz, CH_3_OH-*d*4, ppm): δ_H_ 8.35 (H2, d, *J* 6.9 Hz, 1H), 8.19 (H1′, d, *J* 8.8 Hz, 1H), 7.75 (H4′ and NH, d, *J* 10.2 Hz, 2H), 7.56 (H5, d, *J* 7.2 Hz, 1H), 7.40 (H8, d, *J* 3.2 Hz, 1H), 7.33 (H6, d, *J* 7.3 Hz, 1H), 6.50 (H3, d, *J* 6.8 Hz, 1H), 3.27 (H14, m, 2H), 3.25 (H9, m, 2H), 1.75 (H13, m, 2H, 1.69 (H10, m, 2H), 1.56–1.47 (H11 and H12, m, 4H); ^13^C NMR (101 MHz; DMSO-*d*_6_, ppm): δ_c_ 156.9, 152.5, 151.7, 151.0, 149.7, 149.1, 137.8, 137.2, 128.3, 127.0, 126.8, 125.1, 122.9, 122.6, 122.1, 118.6, 98.9, 43.5, 30.6, 28.2, 27.5, 27.1, 26.9. HRMS (TOF MS ES^+^): found M + 1: 488.1081, C_23_H_24_N_5_OSCl_2_ requires MH^+^, 488.1079.

*1-(6-Bromobenzo[d]thiazol-2-yl)-3-(6-((7-chloroquinolin-4-yl)amino)hexyl)urea* (**6s**). Pale cream colour; yield: 82.32%; m.p. 192–194 °C; FT-IR (*v_max_*/cm^−1^): *v* (C=O) 1533.51, *v* (NH) 2861.00, 2935.51, 3234.26; ^1^H NMR (400 MHz, DMSO-*d*_6_, ppm): δ_H_ 10.93, (NH, brs, 1H), 9.32 (NH, brs, NH), 8.65 (H2, d, *J* 8.8 Hz, 1H), 8.58 (H1′, d, *J* 7.6 Hz, 1H), 8.12 (H4′, s, 1H), 7.97 (H8, s, 1H), 7.75 (H5, d, *J* 9.3 Hz, 1H), 7.51 (H2′, m, 1H), 7.48 (H6, m, 1H), 7.21 (NH, t, *J* 5.0 Hz, 1H), 6.88 (H3, d, *J* 8.5 Hz, 1H), 3.51 (H14, m, 2H), 3.21 (H9, m, 2H), 1.71 (H13, m, 2H), 152–1.32 (H10, H11 and H12, m, 6H); ^13^C NMR (101 MHz; DMSO-*d*_6_, ppm): δ_c_ 161.2, 156.3, 154.2, 148.7, 144.3, 139.3, 138.2, 134.3, 129.1, 127.5, 126.5, 124.9, 122.1, 119.7, 116.3, 115.2, 98.7, 44.6, 30.9, 30.7, 27.5, 26.5, 26.0. HRMS (TOF MS ES^+^): found M + 1: 432.0576. C_23_H_24_N_5_OBrSCl requires MH^+^, 432.0573.

*1-(6-((7-Chloroquinolin-4-yl)amino)hexyl)-3-(6-fluorobenzo[d]thiazol-2-yl)urea* (**6t**). Yellow solid; yield: 92.0%; m.p. 256–259 °C; ^1^H NMR (400 MHz, DMSO-*d*_6_, ppm): δ_H_ 10.75 (NH, brs, 1H), 9.75 (NH, brs, 1H), 8.65 (H2, *J* 8.3 Hz, 1H), 8.58 (H1′, d, *J* 6.8 Hz, 1H), 8.0 (H4′, s, 1H), 7.75 (H2′, m, 1H), 7.60 (H5 and H8, m, 2H), 7.16 (H6, m, 1H), 6.81 (H3, d, *J* 7.8 Hz, 1H), 3.61 (H14, m, 2H), 3.36 (H9, m, 2H), 1.75 (H13, m, 2H), 1.61–1.31 (H10, H11 and H12, m, 6H); ^13^C NMR (101 MHz; DMSO-*d*_6_, ppm): δ_c_ 162.3, 161.9, 157.3, 155.9, 154.3, 147.6, 144.3, 139.0–138.9 (*C3′ coupling to F atom*), 133.2, 127.6, 126.1, 122.3, 121.4, 117.9, 114.9–114.3 (*C2′ coupling to F atom*), 108.6–108.2 (*C4′ coupling to F atom*), 98.2, 43.9, 30.1, 29.9, 28.6, 28.0, 27.6, 26.9. HRMS (TOF MS ES^+^): found M + 1: 472.1374, C_23_H_24_N_5_OFSCl requires MH^+^, 472.1374.

*1-(1-((7-Chloroquinolin-4-yl)amino)propan-2-yl)-3-(6-(trifluoromethyl)benzo[d]thiazol-2-yl)urea* (**6u**). Yellow brown powder; yield: 84.81%; m.p. 243–245 °C; IR (cm^−1^): 3298, 1557 (NH), 1661 (C=C), 1562 (C=O), 1373 (C-N); ^1^H NMR (400 MHz, DMSO-*d*_6_, ppm): δ_H_ 11.13 (NH, brs, 1H), 9.47 (NH, brs, 1H), 8.69 (H2 and H1′, d, *J* 7.4 Hz, 2H), 8.32 (H8, s, 1H), 7.95 (H4′, s, 1H), 7.75 (H2′, d, *J* 7.6 Hz, 1H), 7.63 (H5, d, *J* 8.7 Hz, 1H), 7.31 (H6, d, *J* 8.7 Hz, 1H), 7.13 (H3, d, *J* 7.1 Hz, 1H), 4.18 (H10, m, 1H), 3.74 (H9a, m, 1H), 3.63 (H9b, m, 1H), 1.30 (H11, d, *J* 5.9 Hz, 3H); ^13^C NMR (101 MHz, DMSO-*d*_6_, ppm): δ_C_ 163.1, 156.2, 154.3, 143.7, 139.4, 138.3, 136.9, 133.1, 127.2 (2 × C), 126.9, 126.1, 123.1, 121.0, 119.8, 116.1, 99.4, 48.5, 45.3, 18.5. HRMS (TOF MS ES^+^): found M + 1: 480.0885, C_21_H_18_N_5_OSF_3_Cl requires MH^+^, 480.0873.

*1-(1-((7-Chloroquinolin-4-yl)amino)propan-2-yl)-3-(6-fluorobenzo[d]thiazol-2-yl)urea* (**6v**). Yellow brown powder; yield: 87.52%; m.p. 228–230 °C; IR (cm^−1^): 3356, 1547 (NH), 1676 (C=C), 1576 (C=O), 1377 (C-N); ^1^H NMR (400 MHz, DMSO-*d*_6_, ppm): δ_H_ 9.57 (NH, brs, 1H), 8.58 (H2 and H-1′, m, 2H), 8.31 (H4′, s, 1H), 7.96 (H8, d, *J* 2.0 Hz, 1H), 7.73 (H5, d, *J* 8.8 Hz, 1H), 7.63 (H6, dd, *J* 8.8, 2.0 Hz, 1H), 7.36 (H2′, d, *J* 7.7 Hz, 1H), 7.07 (H3, d, *J* 7.1 Hz, 1H), 4.16 (H-10, m, 1H), 3.73 (H9a, m, 1H), 3.62 (H9b, m, 1H), 1.26 (H11, d, *J* 6.7 Hz, 3H); ^13^C NMR (101 MHz, DMSO-*d*_6_, ppm): δ_c_ 207.2, 157.3, 152.9, 149.6, 141.5, 136.3, 135.5, 134.0, 131.3, 129.7, 126.3, 125.1, 124.3, 123.1, 122.5, 99.8, 32.3, 27.1, 18.2. HRMS (TOF MS ES^+^): found M + 1: 430.0910, C_20_H_18_N_5_OSFCl requires MH^+^, 430.0905.

*1-(1-((7-Chloroquinolin-4-yl)amino)propan-2-yl)-3-(6-bromobenzo[d]thiazol-2-yl)urea* (**6w**). Yellow powder; yield: 89.99%; m.p. 246–248 °C; IR (cm^−1^): 3245, 1525 (NH), 1672 (C=C), 1571 (C=O), 1335 (C-N); ^1^H NMR (400 MHz, DMSO-*d*_6_, ppm): δ_H_ 8.45 (H2, d, *J* 8.7 Hz, 1H), 8.29 (H1′, d, *J* 9.0 Hz, 1H), 7.99 (H4′, s, 1H), 7.70 (H8, s, 1H), 7.61 (H5, d, *J* 7.9 Hz, 1H), 7.51 (H2′, d, *J* 9.0 Hz, 1H), 7.39 (H6, d, *J* 7.7 Hz, 1H), 6.89 (H3, d, *J* 8.5 Hz, 1H), 6.64 (NH, s, 1H), 4.14 (H-10, m, 1H), 3.48 (H9a, m, 1H and *H9b overlaps with water peak), 1.23 (H-11, d, *J* = 6.7 Hz, 3H); ^13^C NMR (101 MHz, DMSO-*d*_6_, ppm): δ_c_ 207.2, 157.3, 152.9, 149.6, 141.5, 136.3, 135.5, 134.0, 131.3, 129.7, 126.3, 125.1, 124.3, 123.1, 122.5, 99.8, 32.3, 27.1, 18.2. HRMS (TOF MS ES^+^): found M + 1: 490.0103, C_20_H_18_N_5_OSBrCl requires MH^+^, 490.0104.

*1-(1-((7-Chloroquinolin-4-yl)amino)propan-2-yl)-3-(6-methylbenzo[d]thiazol-2-yl)urea* (**6x**). Brown yellow powder; yield: 78.81%; m.p. 229–231 °C; IR (cm^−1^): 3359, 1542 (NH), 1672 (C=C), 1615 (C=O), 1346 (C-N); ^1^H NMR (400 MHz, DMSO-*d*_6_, ppm): δ_H_ 9.67 (NH, t, *J* 5.1 Hz, 1H), 8.66 (H2, d, *J* 9.1 Hz, 1H), 8.57 (H4′, s, 1H), 8.04 (H1′, *J* 2.1 Hz, 1H), 7.74 (H5, d, *J* 8.3 Hz, 1H), 7.62 (H8, s, 1H), 7.53 (H2′ and NH, m, 2H), 7.16 (H6, d, *J* 8.3 Hz, 1H), 7.08 (H3, d, *J* 9.1 Hz, 1H), 4.14 (H10, m, 1H), 3.70 (H9a, m, 1H), 3.56 (H9b, m, 1H), 2.36 (H5′, s, 3H), 1.26 (H11, d, *J* 6.7 Hz, 3H); ^13^C NMR (101 MHz, DMSO-*d*_6_, ppm): δ_c_ 159.3, 157.2, 156.4, 146.3, 144.6, 138.9, 138.6, 132.4, 131.6, 129.9, 129.6, 129.3, 127.7, 122.5, 119.4, 116.9, 99.3, 48.5, 45.1, 21.3, 18.6. HRMS (TOF MS ES^+^): found M + 1: 426.1154, C_21_H_21_N_5_OSCl requires MH^+^, 426.1155.

*1-(1-((7-Chloroquinolin-4-yl)amino)propan-2-yl)-3-(6-chlorobenzo[d]thiazol-2-yl)urea* (**6y**). Brownish white powder; yield: 81.19%; m.p. 301–304 °C; IR (cm^−1^): 3254, 1532 (NH), 1672 (C=C), 1646 (C=O), 1343 (C-N); ^1^H NMR (400 MHz, DMSO-*d*_6_, ppm): δ_H_ 11.32 (NH, brs, 1H), 9.51 (NH, s, 1H), 8.69 (H2 and H1′, d, *J* 5.8 Hz, 2H), 8.39 (H4′, s, 1H), 7.96 (H8, s, 1H), 7.79 (H5 and NH, d, *J* 6.9 Hz, 2H), 7.70 (H2′, d, *J* 3.8 Hz, 1H), 7.32 (H6, d, *J* 7.1 Hz, 1H), 7.12 (H3, d, *J* 5.8 Hz, 1H), 4.12-4.06 (H-10, m, 1H), 3.43-3.01 (H9, m, 2H) 1.26 (H-11, d, *J* = 3.4 Hz, 3H); ^13^C NMR (101 MHz, DMSO-*d*_6_, ppm): δ_c_ 160.88, 154.77, 152.71, 149.18, 147.72, 147.69, 142.83 135.49, 133.61, 130.02, 127.09, 126.46, 125.53, 121.18, 118.30 117.21, 99.34, 48.28, 45.12, 18.70. HRMS (TOF MS ES^+^): found M + 1: 446.0699, C_20_H_18_N_5_OSCl_2_ requires MH^+^, 446.0609.

### 3.3. In Vitro Antitubercular Activity

The minimum inhibitory concentration (MIC) is determined using the standard broth microdilution method, according to which a 10 mL culture of *Mycobacterium tuberculosis* H_37_Rv pMSp12::GFP [54,55,56] is grown to an optical density (OD600) of 0.6–0.7. Cultures are diluted prior to the inoculation of assays as follows:

(i) 1:500 in middelbrook 7H9 supplemented with 0.03% casitone, 0.4% glucose, and 0.05% tyloxapol [57,58];
(ii) 1:500 in 7H9 supplemented with 10% Albumin Dextrose Catalase supplement (ADC), 0.05% Tween-80, 0.2% glucose [59,60].

The compounds to be tested are reconstituted to a concentration of 10 mM in DMSO. Two-fold serial dilutions of the test compound are prepared across a 96-well black microtiter plate, after which 50 μL of the diluted *M. tuberculosis* cultures is added to each well in the serial dilution in triplicate. The plate layout is a modification of the method previously described [61]. The assay controls used are a minimum growth control (rifampicin at 2 × MIC) and a maximum growth control (5% DMSO). The microtiter plates are sealed in a secondary container and incubated at 37 °C with 5% CO_2_ and humidification. Relative fluorescence (excitation 485 nM; emission 520 nM) is measured and the assay is set up in a black 96-well microtiter plate. Relative fluorescent readings are taken at day 7, using Spectramax i3x, and the lowest concentration of material displaying no visible growth is scored as the MIC_90_.

The microtiter plats are re-incubated for a further 7 days. Fluorescent readings are taken after 7 days again and reported as day 14 data.

Raw fluorescent data (relative fluorescent units) are acquired using a SpectraMax i3x Plate reader (serial No. 36370 3271), Molecular Devices Corporation 1311 Orleans Drive Sunnyvale, California 94089.

RFU Data are analysed using Softmax^®^ Pro 6 software: (Version 6.5.1, serial No. 1278552768867612530), Molecular Devices Corporation 1311 Orleans Drive Sunnyvale, California 94089.

The onboard fluorescent intensity endpoint protocol is used in conjunction with the following wavelength filters: excitation: 485; emission: 520. The Softmax^®^ Pro 6, 4-parameter curve fit protocol is used to generate a calculated MIC_90_. Raw RFU data are normalised to the minimum and maximum inhibition controls to generate a dose–response curve (percent inhibition), using the Levenberg–Marquardt damped least-squares method, from which the MIC_90_ is calculated. The lowest concentration of drug that inhibits 90% of growth of the bacterial population is considered to be the MIC_90_.

### 3.4. In Vitro MTT Cytotoxicity Studies

HepG2 liver cells (Highveld Biological, Johannesburg, South Africa) are characterised as non-tumorigenic cells with high proliferation rates. Originally derived from a Caucasian male, the cells have retained an epithelial-like morphology and key differentiated liver function, and are thus deemed a suitable model for cytotoxicity testing [62,63].

HepG2 were aseptically maintained at 37 °C (5% CO_2_) until confluent in complete culture medium (CCM) comprising Dulbecco’s Minimum Essential Medium, 10% foetal bovine serum, 1% L-glutamine, and 1% pen–strep–fungizone. The confluent HepG2 cells were rinsed with 0.1 M phosphate-buffered saline (PBS), then trypsinised and counted to facilitate their transfer to a 96-well microtiter plate (2 × 10^4^ cells/150 µL CCM/well). After incubation overnight to ensure adherence to the plate wells, the CCM was discarded and the cells were treated with varying concentrations (0–200 µM) of the synthesized compounds for 24 h (37 °C, 5% CO_2_). A vehicle control (1% DMSO in CCM) was included. The treatment medium was removed and the wells were replenished with 20 µL MTT solution (5 mg/mL MTT in PBS) and 100 µL CCM. After 4 h (37 °C, 5% CO_2_), the MTT/CCM solution was replaced with 100 µL DMSO (1 h, 37 °C, 5% CO_2_) to solubilize the formazan crystals. The absorbance was read at 570 nm/690 nm using the SPECTROstar Nano (BMG Labtech, Ortenberg, Germany). Percentage cell viability was calculated using the absorbance values ($\frac{absorbance\;of\;treatment}{absorbance\;of\;control}$ × 100). A nonlinear regression analysis (GraphPad Prism V5.0, GraphPad PRISM^®^, La Jolla, CA, USA) for cell viability vs. log treatment concentration was performed to extrapolate the 20% inhibitory concentration (IC_20_); at this concentration cells retained 80% cell viability. The cell viability at the respective 90% *Mtb* inhibitory concentration (MIC_90_ in the CAS assay) was also extrapolated from the curve.

### 3.5. In Silico ADME Profiling

QikProp (Schrödinger, LLC, New York, NY, USA) was utilized for predicting physically relevant descriptors for predicting potential drug-likeness, such as human oral absorption, QlogHERG, QlogBB, QlogKhsa, and the compliance to the Lipinski rule of five.

## 4. Conclusions

In this study, 25 new quinoline–urea–benzothiazole hybrids (**6a**–**y**) have been successfully synthesized via a three-step synthetic protocol in yields ranging from 50 to 90%. These compounds were then evaluated for their MIC_90_ anti-*Mtb* activity, in protein-rich (ADC) and -poor (CAS) media, with activities ranging from 0.97–62.5 μM. The anti-*Mtb* activities suggest that the type of the diamine used and its chain length, as well as the substituent on the benzothiazole moiety, influences activity. For example, (i) CF_3_-containing compounds (**6b**, **6i** and **6u**) on the benzothiazole moiety displayed the most consistent activities across the two media, with **6u** being the most active of all the compounds in this study; and (ii) the propane-1,2-diamine (sub-series **6u**–**y**) was the most preferred diamine, as this produced compounds with activities less than 15 µM across the board, even though **6u** exhibited a potential for protein binding. The hybridization strategy was also found to be advantageous and the intermediate making up the hybrids appeared to function in a synergistic manner since on their own and in combination they are devoid of any activity. Lastly, based on the data, compound **6u** seems to be a very good starting point in the quest for the next generation of hybrid compounds.

Overall, minimal cytotoxicity to HepG2 cells (cell viability above 75%) was observed in 11 compounds, indicating that they may be exploited for potential antimycobacterial effects, including activity under different growth conditions thought to mimic human infection, for example, nutrient starvation, hypoxia, and/or low pH. The cytotoxicity data also demonstrate the anti-proliferative activities of the synthesized compounds in HepG2 cells. Thus, further investigation into the possible anticancer effects of the synthesized compounds and their possible anti-*Mtb* mode of action are warranted in future. Generally, all the synthesized compounds showed favourable predicted in silico profiles against the majority of the selected descriptors. More importantly, these compounds were predicted to have a reduced potential to inhibit the cardiac potassium hERG channel (QlogHERG > −5).

## Figures and Tables

**Table 1:** Yields and melting points of target hybrid compounds **6a**–**y**.

Compound	Diamine Linker	X	m.p. (°C)	Yield (%)
**6a**	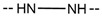	H	298–302	88.52
**6b**		CF_3_	343–345	84.81
**6c**		F	340–342	86.11
**6d**		NO_2_	339–341	78.75
**6e**	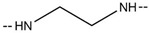	CF_3_	249–251	64.90
**6f**		Cl	244–245	83.23
**6g**		Br	240–242	78.41
**6h**		F	230 –232	75.23
**6i**	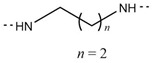	CF_3_	198–200	83.42
**6j**		Cl	286–288	76.82
**6k**		CH_3_	234–239	55.94
**6l**		H	285–288	59.31
**6m**		Br	254–257	60.20
**6n**		F	241–243	78.50
**6o**	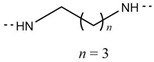	Cl	189–191	83.04
**6p**		Br	192–194	82.34
**6q**		F	256–259	92.05
**6r**	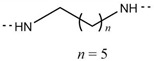	Cl	239–241	79.72
**6s**		Br	192–194	82.32
**6t**		F	256–259	92.03
**6u**	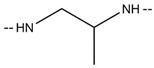	CF_3_	243–245	89.01
**6v**		F	228–230	87.52
**6w**		Br	246–248	89.99
**6x**		CH_3_	229–231	78.81
**6y**		Cl	301–304	81.19

**Table 2:** In vitro 90% *Mtb* inhibition in 7H0/CAS/Glu/Tx and 7H9/ADC/Glu/Tw with clogP values of the target compounds **6a**–**y**.

Compound	^a^ 7H9/CAS/GLU/Tx	^b^ 7H9/ADC/GLU/Tw	^c^ clogP
	7 Days (µM)	7 Days (µM)	
**6a**	21.001	>125	4.32
**6b**	4.943	6.854	5.22
**6c**	>125	>125	4.44
**6d**	>125	>125	4.10
**6e**	ND	ND	6.35
**6f**	125	>125	6.10
**6g**	8.89	14.898	6.25
**6h**	62.5	62.5	5.53
**6i**	7.812	12.837	6.72
**6j**	4.389	11.748	6.48
**6k**	31.25	62.33	6.25
**6l**	9.628	9.447	5.75
**6m**	15.924	16.863	6.63
**6n**	>125	125	5.91
**6o**	7.455	23.529	6.49
**6p**	7.812	15.609	6.64
**6q**	>125	>125	5.92
**6r**	7.597	14.617	7.54
**6s**	8.76	20.954	7.69
**6t**	6.974	31.25	6.97
**6u**	0.968	5.732	6.66
**6v**	>125	>125	5.84
**6w**	8.191	14.001	6.56
**6x**	7.219	10.35	6.19
**6y**	2.331	8.455	6.41
**2e**	>125	>125	4.07
**4b**	>125	>125	1.97
**5b**	>125	>125	0.474
**Urea**	>125	>125	−1.66
**2e:4b:Urea (1:1:1)**	>125	>125	-
**RIF**	0.03	0.001	3.71

^a^ Protein-poor *Mtb* media. ^b^ Protein-rich *Mtb* media; ND = not determined. ^c^ Calculated using PerkinElmer ChemDraw Professional V18.0.0.231 [46].

**Table 3:** In silico ADME and drug-likeness properties of the selected compounds.

Compound	% Human Oral Absorption ^a^	QPlogHERG ^b^	QPlogBB ^c^	QPlogKhsa ^d^	Ro5 ^e^
**6a**	91	−5.3	−0.594	−0.093	0
**6b**	100	−5.235	−0.351	0.142	0
**6c**	93	−5.169	−0.487	−0.056	0
**6d**	71	−5.211	−1.744	−0.117	0
**6e**	100	−5.661	−0.371	0.363	0
**6f**	100	−5.094	−0.378	0.184	0
**6g**	100	−5.119	−0.369	0.205	0
**6h**	100	−5.062	−0.425	0.116	0
**6i**	100	−5.748	−0.648	0.458	0
**6j**	100	−5.695	−0.48	0.313	0
**6k**	100	−5.854	−0.865	0.382	0
**6l**	100	−5.809	−0.634	0.203	0
**6m**	100	−5.725	−0.477	0.337	0
**6n**	100	−5.676	−0.529	0.243	0
**6o**	100	−5.884	−0.831	0.433	0
**6p**	91	−5.904	−0.822	0.456	1
**6q**	100	−5.851	−0.843	0.427	0
**6r**	100	−6.077	−0.863	0.637	1
**6s**	83	−6.212	−0.904	0.695	2
**6t**	96	−6.049	−0.908	0.563	1
**6u**	95	−5.776	−0.319	0.511	1
**6v**	100	−5.736	−0.459	0.303	0
**6w**	100	−5.777	−0.402	0.396	0
**6x**	100	−5.749	−0.593	0.414	0
**6y**	100	−5.753	−0.409	0.374	0

^a^ % Human Oral Absorption: Predicted human absorption on 0 to 100% scale (recommended: >80% is good and <25% is poor). ^b^ QPlog HERG: Predicted IC_50_ values for blockage of HERG K^+^ channels (below −5 is a concern). ^c^ QPlogBB: Predicted brain–blood partition coefficient (recommended: −3.0–1.2 range). ^d^ QPlogKhsa: Prediction of binding to human serum albumin (recommended: −1.5–1.5 range). ^e^ Ro5: Number of violations of Lipinski’s rule (recommended: a maximum of four violations).

## Data Availability

Data is contained within the article and Supplementary Material.

## Supplementary Materials

The following supporting information can be downloaded at: https://www.mdpi.com/article/10.3390/ph15050576/s1, Figures S1–S74: ^1^H, ^13^C NMR and HRMS for **6a**–**y**.
