# Supplementary material for: New Quinoline–Urea–Benzothiazole Hybrids as Promising Antitubercular Agents: Synthesis, In Vitro Antitubercular Activity, Cytotoxicity Studies, and In Silico ADME Profiling
Source: Pharmaceuticals (Basel). 2022 May 5;15(5):576. doi: 10.3390/ph15050576 (PMC9146500; doi:10.3390/ph15050576)
Supplement: Supplementary file 1 [file pharmaceuticals-15-00576-s001.zip › pharmaceuticals-1704276-supplementary.pdf]

New quinoline-urea-benzothiazole hybrids as promising antitubercular agents: Synthesis, *in vitro* antitubercular activity, cytotoxicity studies, and *In in silico* ADME profiling

*Rashmika Moodley[1#], Chakes Mashaba[1#], Goitsemodimo H. Rakodi[2], Nomagugu B. Ncube[1], Mabuatsela V. Maphoru[2], Mohammed O. Balogun[3], Audrey Jordan[4], Digby F. Warner[4,5,6], Rene Khan[7], Matshawandile Tukulula[1]**

[1]School of Chemistry and Physics, University of KwaZulu Natal, Westville Campus, Durban 4000, South Africa.

[2]Department of Chemistry, Tshwane University Technology, Pretoria 0001, South Africa.

[3]Bio-polymer Modification and Therapeutics Laboratory, Council for Scientific and Industrial Research (CSIR), Pretoria 0001, South Africa.

[4]SAMRC/NHLS/UCT Molecular Mycobacteriology Research Unit, Department of Pathology, University of Cape Town, Medical School Campus, Observatory 7925, South Africa.

[5]Institute of Infectious Disease and Molecular Medicine, University of Cape Town, Medical School Campus, Observatory 7925, South Africa.

[6]Welcome Centre for Infectious Diseases Research in Africa (CIDRI-Africa), University of Cape Town, Medical School Campus, Observatory 7925, Cape Town.

[7]Department of Medical Biochemistry, School of Laboratory Medicine and Medical Science, College of Health Sciences, University of KwaZulu Natal, Howard Campus, Durban 4001, South Africa.

*Corresponding author(s): Email: TukululaM@ukzn.ac.za Tel 031 260 8756 Fax 031 260 3091;

#Equal contribution

REPRESENTATIVE NMR AND HRMS SPECTRA OF FINAL COMPOUNDS

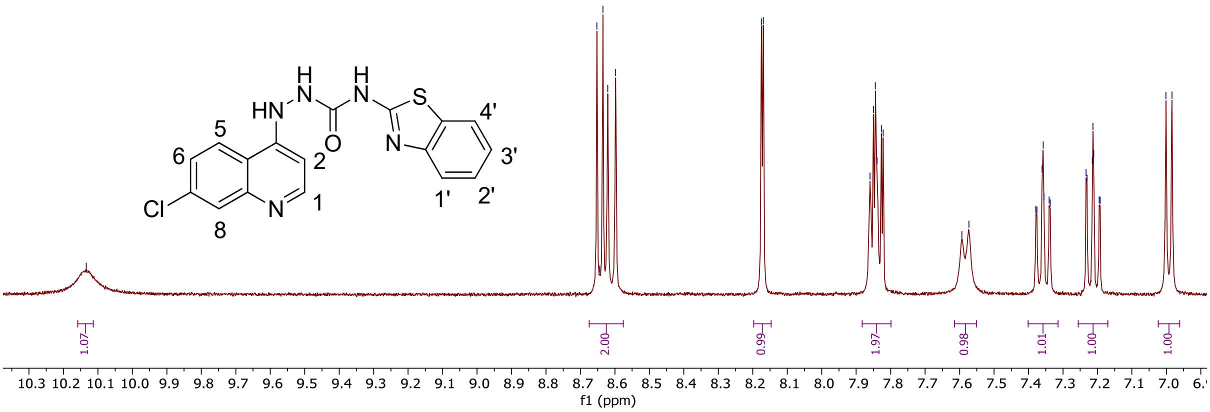

Figure S1: 400 MHz ^{1}H-NMR *N-(benzo[d]thiazol-2yl)-2-(7-chloroquinolin-4-yl)hydrazine-1-carboxamide in DMSO-d6 (**6a**).*

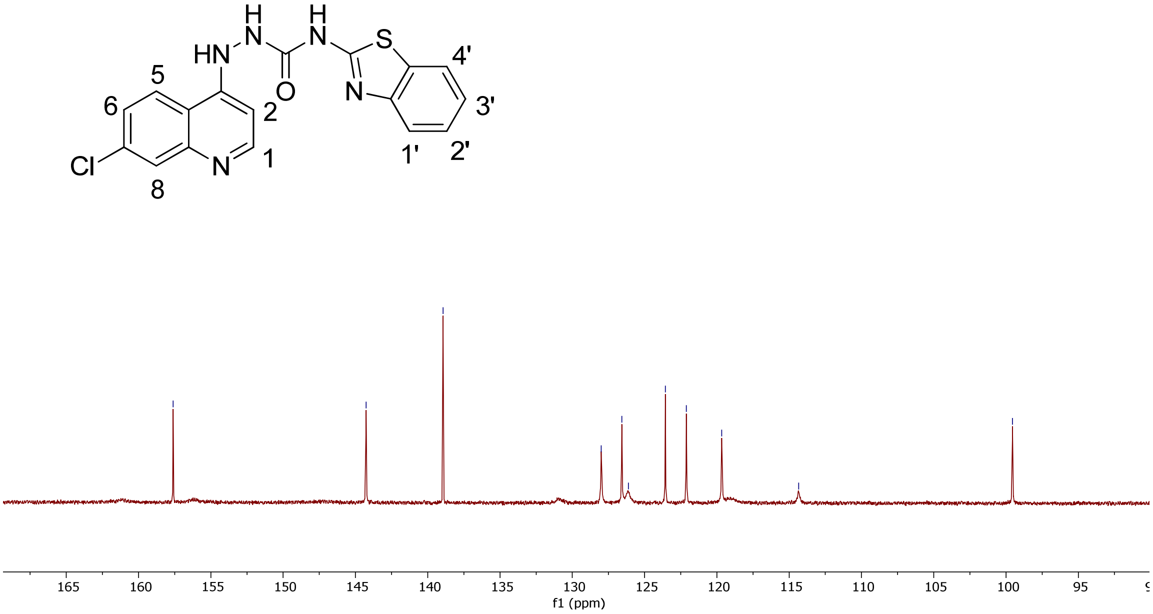

Figure S2: 101 MHz ^{13}C-NMR *N-(benzo[d]thiazol-2yl)-2-(7-chloroquinolin-4-yl)hydrazine-1-carboxamide in DMSO-d6 (**6a**).*

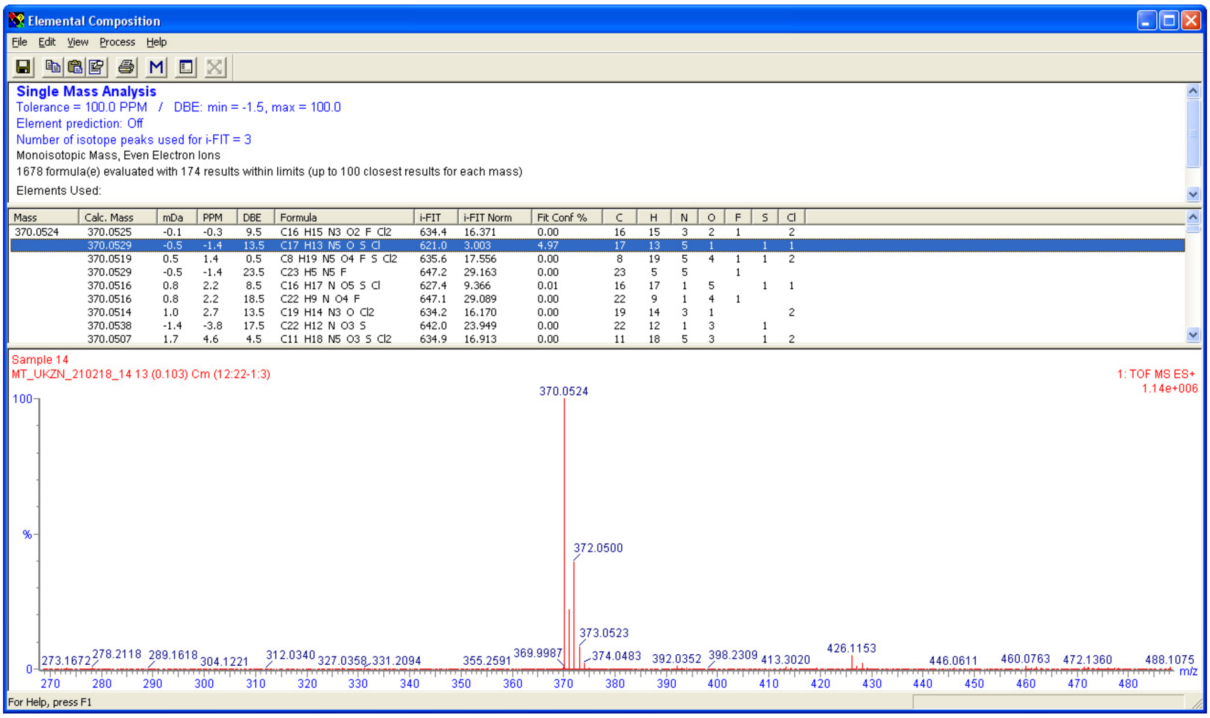

Elemental Composition

File Edit View Process Help

Single Mass Analysis

Tolerance = 100.0 PPM / DBE: min = -1.5, max = 100.0

Element prediction: Off

Number of isotope peaks used for i-FIT = 3

Monoisotopic Mass, Even Electron Ions

1678 formula(e) evaluated with 174 results within limits (up to 100 closest results for each mass)

Elements Used:

Mass	Calc. Mass	mDa	PPM	DBE	Formula	i-FIT	i-FIT Norm	Fit Conf %	C	H	N	O	F	S	Cl
370.0524	370.0525	-0.1	-0.3	9.5	C16 H15 N3 O2 F Cl2	634.4	16.371	0.00	16	15	3	2	1		2
	370.0529	-0.5	-1.4	13.5	C17 H13 N5 O S Cl	621.0	3.003	4.97	17	13	5	1		1	1
	370.0519	0.5	1.4	0.5	C8 H19 N5 O4 F S Cl2	635.6	17.556	0.00	8	19	5	4	1	1	2
	370.0529	-0.5	-1.4	23.5	C23 H5 N5 F	647.2	29.163	0.00	23	5	5		1		
	370.0516	0.8	2.2	8.5	C16 H17 N O5 S Cl	627.4	9.366	0.01	16	17	1	5		1	1
	370.0516	0.8	2.2	18.5	C22 H9 N O4 F	647.1	29.089	0.00	22	9	1	4	1		
	370.0514	1.0	2.7	13.5	C19 H14 N3 O Cl2	634.2	16.170	0.00	19	14	3	1			2
	370.0538	-1.4	-3.8	17.5	C22 H12 N O3 S	642.0	23.949	0.00	22	12	1	3		1	
	370.0507	1.7	4.6	4.5	C11 H18 N5 O3 S Cl2	634.9	16.913	0.00	11	18	5	3		1	2

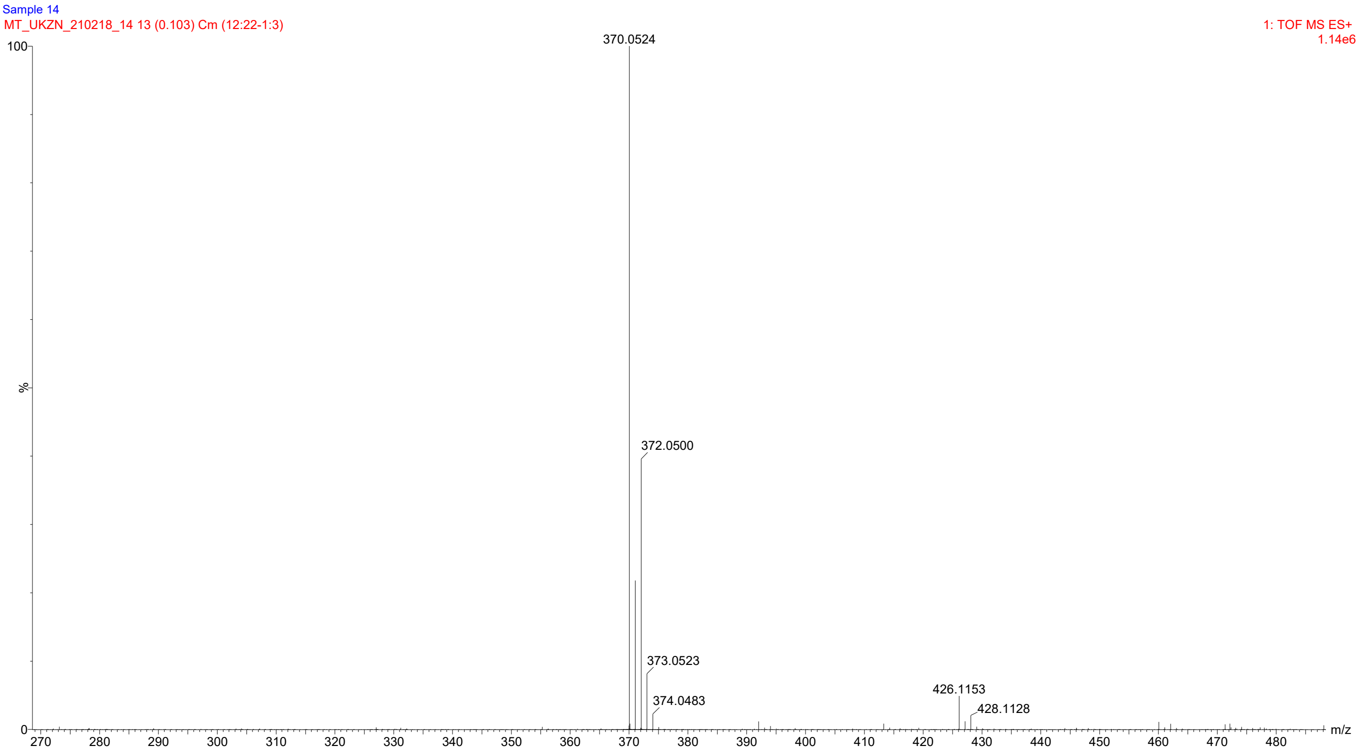

Figure S3: HRMS (ES$^+$) for *N-(benzo[d]thiazol-2yl)-2-(7-chloroquinolin-4-yl)hydrazine-1-carboxamide* (***6a***).

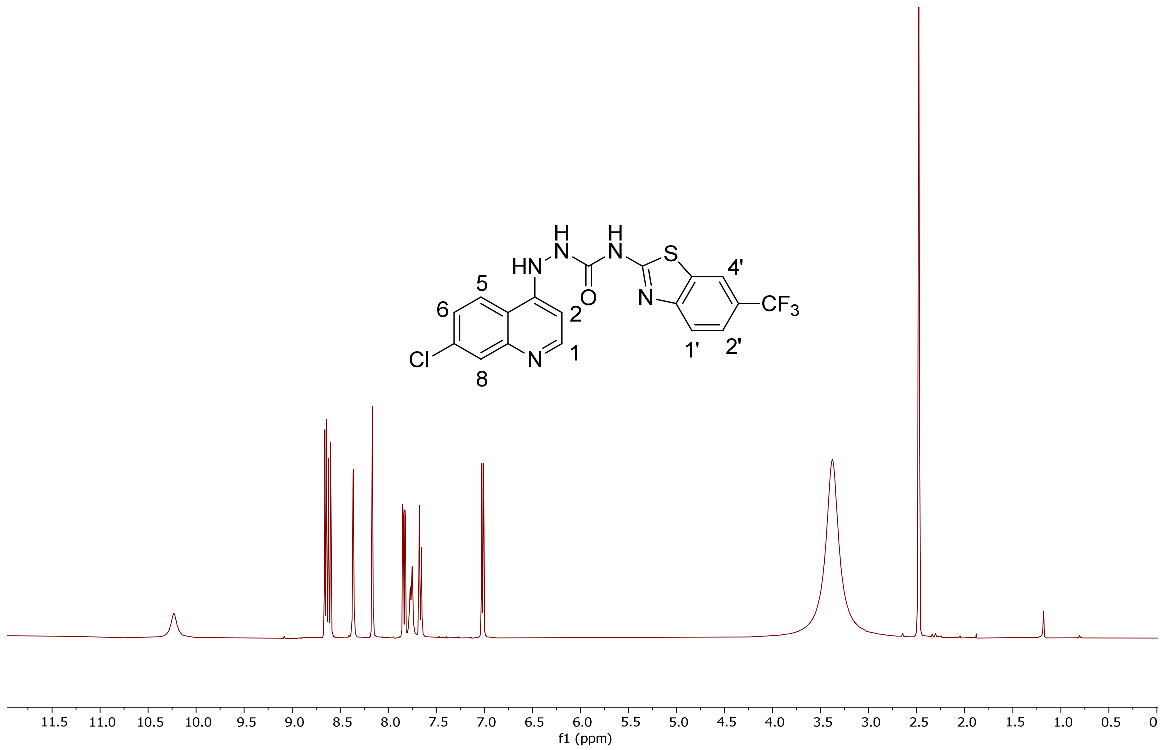

Figure S4: 400 MHz ^{1}H-NMR of *2-(7-chloroquinolin-4-yl)-N-(6-(trifluoromethyl)benzo[d]thiazol-2-yl)hydrazine-1-carboxamide* in DMSO-d6 ***(6b)***.

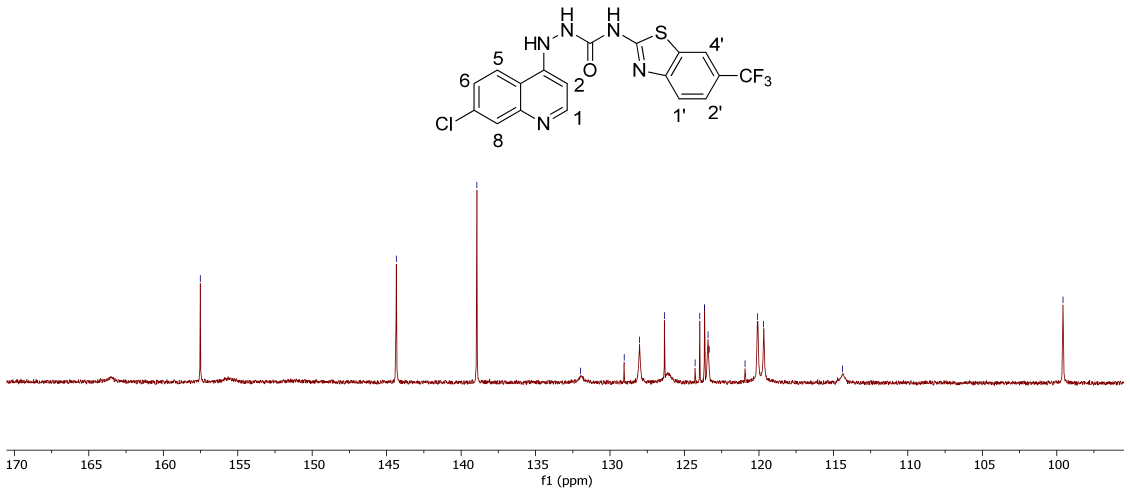

Figure S5:101 MHz ^{13}C-NMR of *2-(7-chloroquinolin-4-yl)-N-(6-(trifluoromethyl)benzo[d]thiazol-2-yl)hydrazine-1-carboxamide in* DMSO-d6 ***(6b)***.

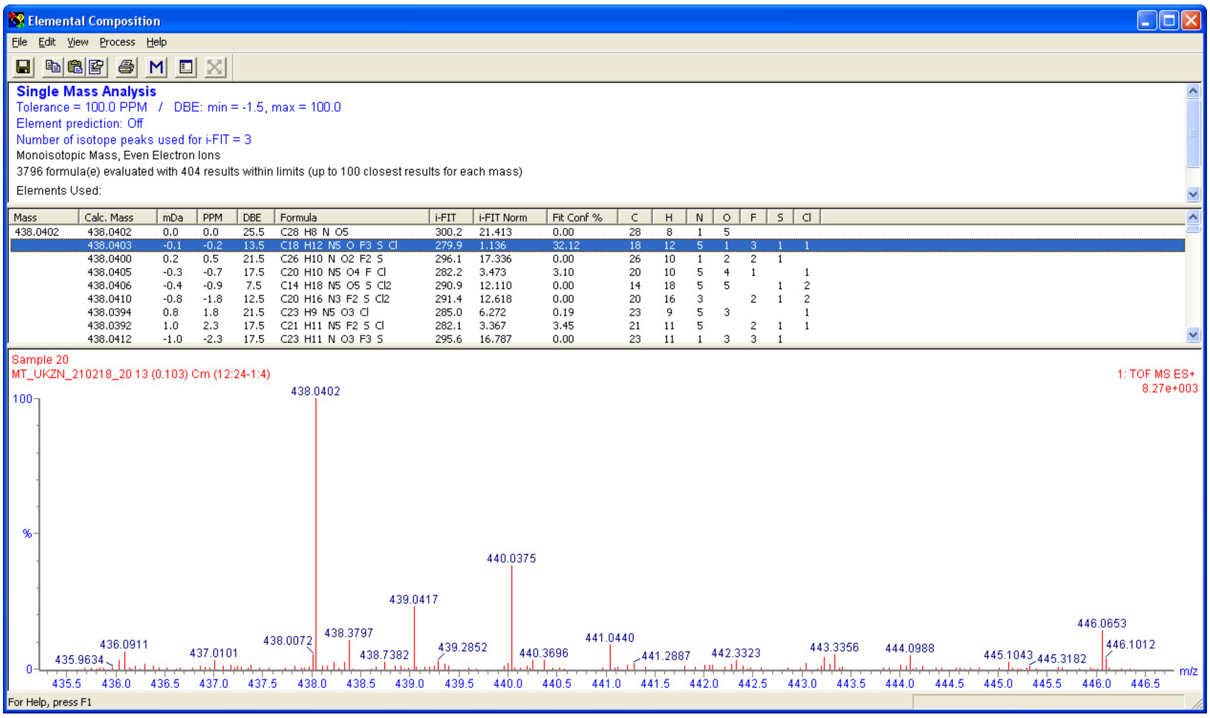

Mass	Calc. Mass	mDa	PPM	DBE	Formula	i-FIT	i-FIT Norm	Fit Conf %	C	H	N	O	F	S	Cl
438.0402	438.0402	0.0	0.0	25.5	C28 H8 N O5	300.2	21.413	0.00	28	8	1	5			
	438.0403	-0.1	-0.2	13.5	C18 H12 N5 O F3 S Cl	279.9	1.136	32.12	18	12	5	1	3	1	1
	438.0400	0.2	0.5	21.5	C26 H10 N O2 F2 S	296.1	17.336	0.00	26	10	1	2	2	1	
	438.0405	-0.3	-0.7	17.5	C20 H10 N5 O4 F Cl	282.2	3.473	3.10	20	10	5	4	1		1
	438.0406	-0.4	-0.9	7.5	C14 H18 N5 O5 S Cl2	290.9	12.110	0.00	14	18	5	5		1	2
	438.0410	-0.8	-1.8	12.5	C20 H16 N3 F2 S Cl2	291.4	12.618	0.00	20	16	3		2	1	2
	438.0394	0.8	1.8	21.5	C23 H9 N5 O3 Cl	285.0	6.272	0.19	23	9	5	3			1
	438.0392	1.0	2.3	17.5	C21 H11 N5 F2 S Cl	282.1	3.367	3.45	21	11	5		2	1	1
	438.0412	-1.0	-2.3	17.5	C23 H11 N O3 F3 S	295.6	16.787	0.00	23	11	1	3	3	1	

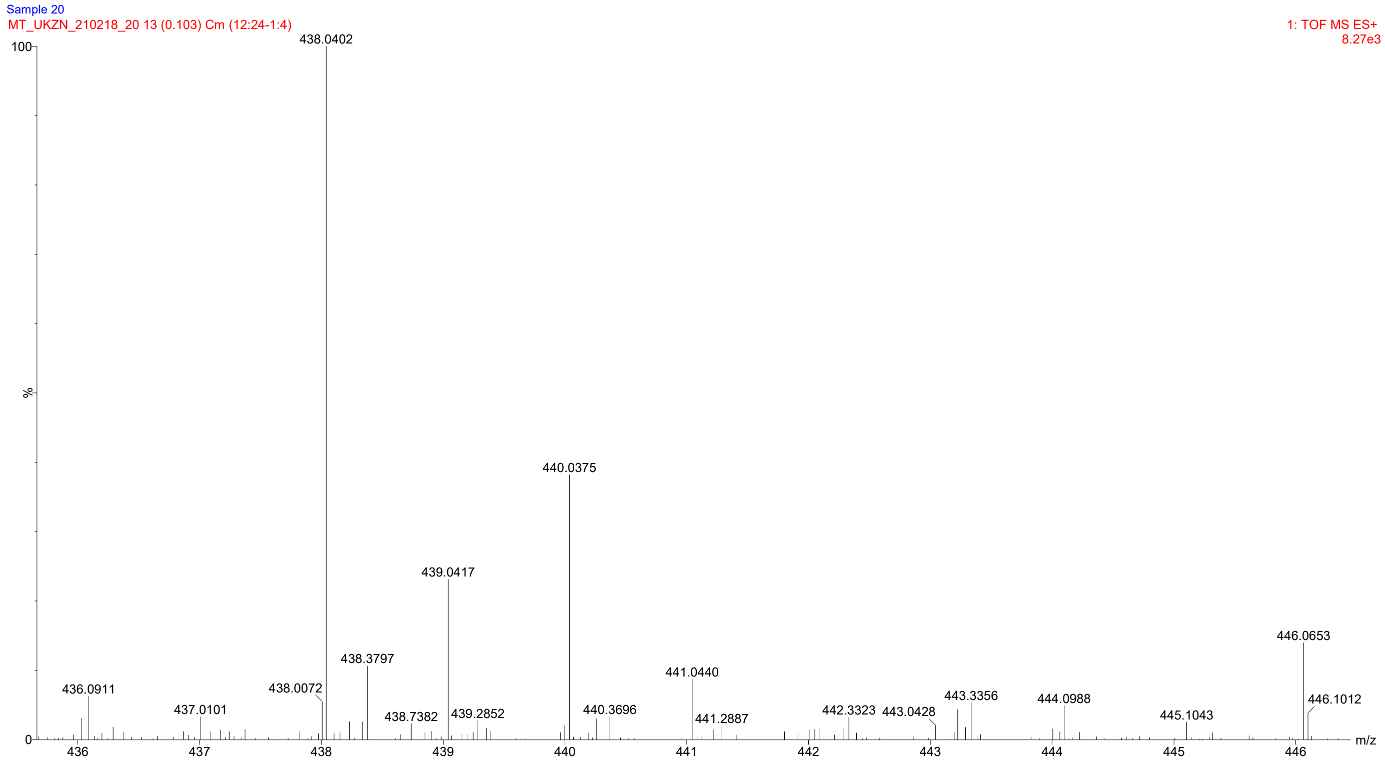

Figure S6: HRMS (ES$^+$) for *2-(7-chloroquinolin-4-yl)-N-(6-(trifluoromethyl)benzo[d]thiazol-2-yl)hydrazine-1-carboxamide* (***6b***).

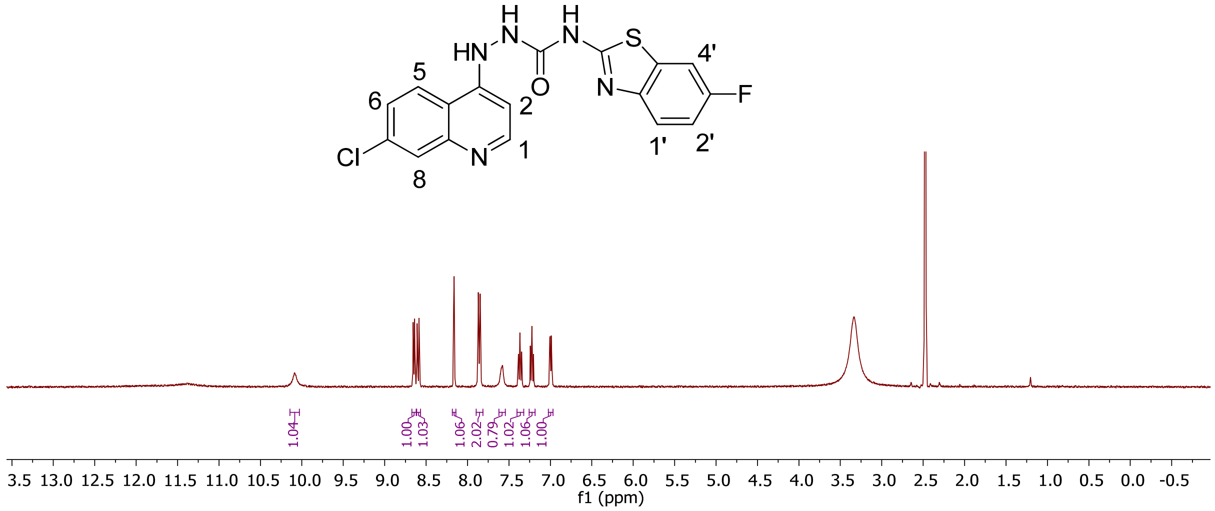

Figure S7: 400 MHz ^{1}H-NMR of *2-(7-chloroquinolin-4-yl)-N-(6-fluorobenzo[d]thiazol-2-yl)hydrazine-1-carboxamide in* DMSO-d6 *(**6c**).*

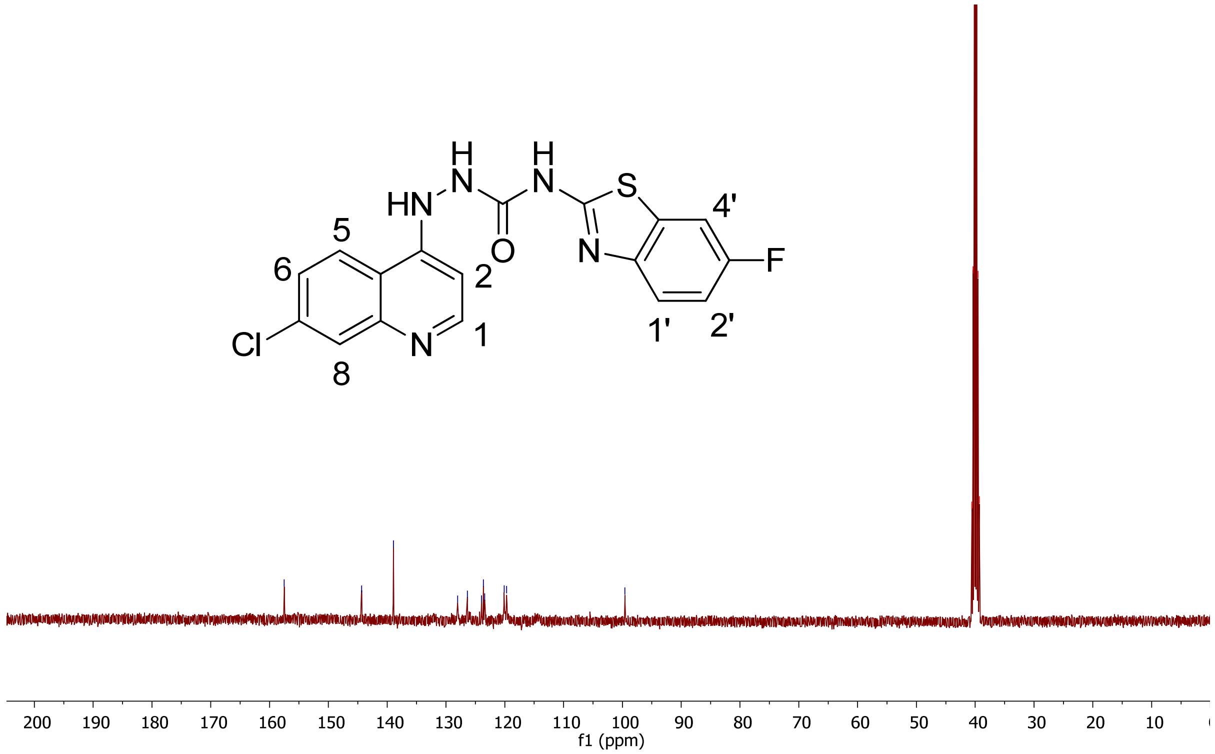

Figure S8: 101 MHz ^{13}C-NMR of *2-(7-chloroquinolin-4-yl)-N-(6-fluorobenzo[d]thiazol-2-yl)hydrazine-1-carboxamide* in DMSO-d6 *(**6c**).*

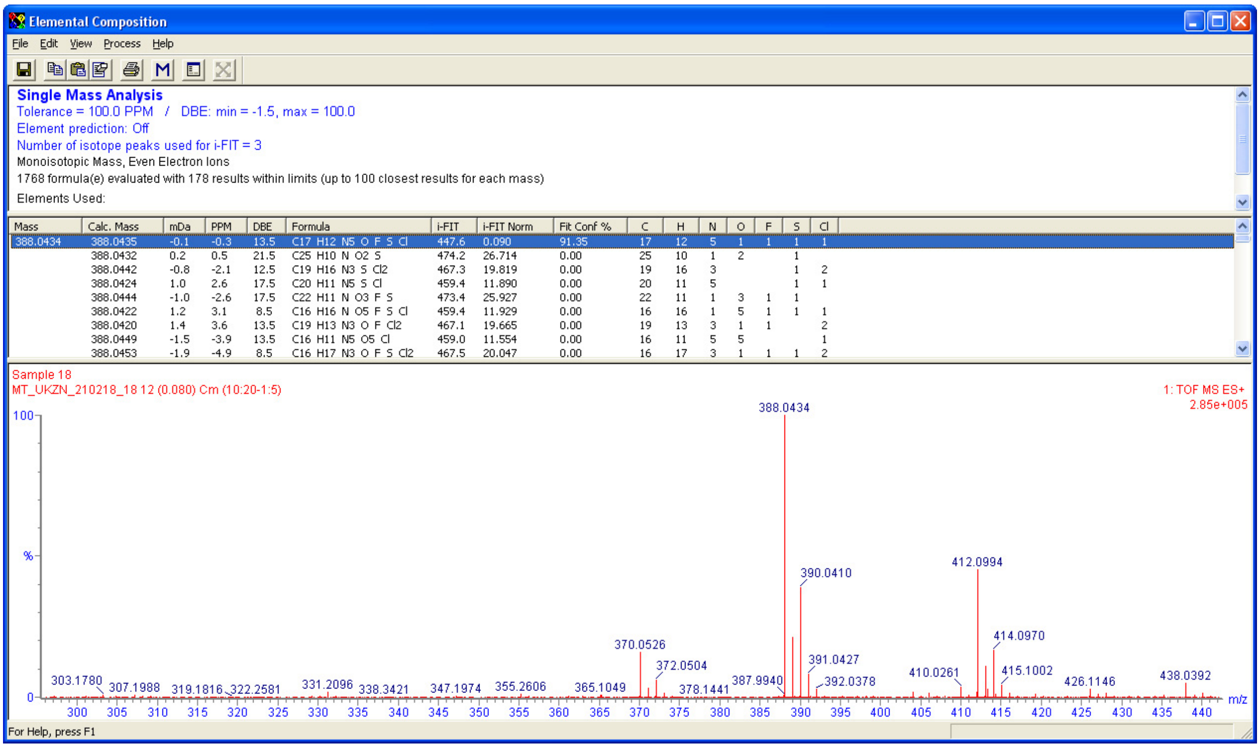

Mass	Calc. Mass	mDa	PPM	DBE	Formula	i-FIT	i-FIT Norm	Fit Conf %	C	H	N	O	F	S	Cl
388.0434	388.0435	-0.1	-0.3	13.5	C17 H12 N5 O F S Cl	447.6	0.090	91.35	17	12	5	1	1	1	1
	388.0432	0.2	0.5	21.5	C25 H10 N O2 S	474.2	26.714	0.00	25	10	1	2		1	
	388.0442	-0.8	-2.1	12.5	C19 H16 N3 S Cl2	467.3	19.819	0.00	19	16	3			1	2
	388.0424	1.0	2.6	17.5	C20 H11 N5 S Cl	459.4	11.890	0.00	20	11	5			1	1
	388.0444	-1.0	-2.6	17.5	C22 H11 N O3 F S	473.4	25.927	0.00	22	11	1	3	1	1	
	388.0422	1.2	3.1	8.5	C16 H16 N O5 F S Cl	459.4	11.929	0.00	16	16	1	5	1	1	1
	388.0420	1.4	3.6	13.5	C19 H13 N3 O F Cl2	467.1	19.665	0.00	19	13	3	1	1		2
	388.0449	-1.5	-3.9	13.5	C16 H11 N5 O5 Cl	459.0	11.554	0.00	16	11	5	5			1
	388.0453	-1.9	-4.9	8.5	C16 H17 N3 O F S Cl2	467.5	20.047	0.00	16	17	3	1	1	1	2

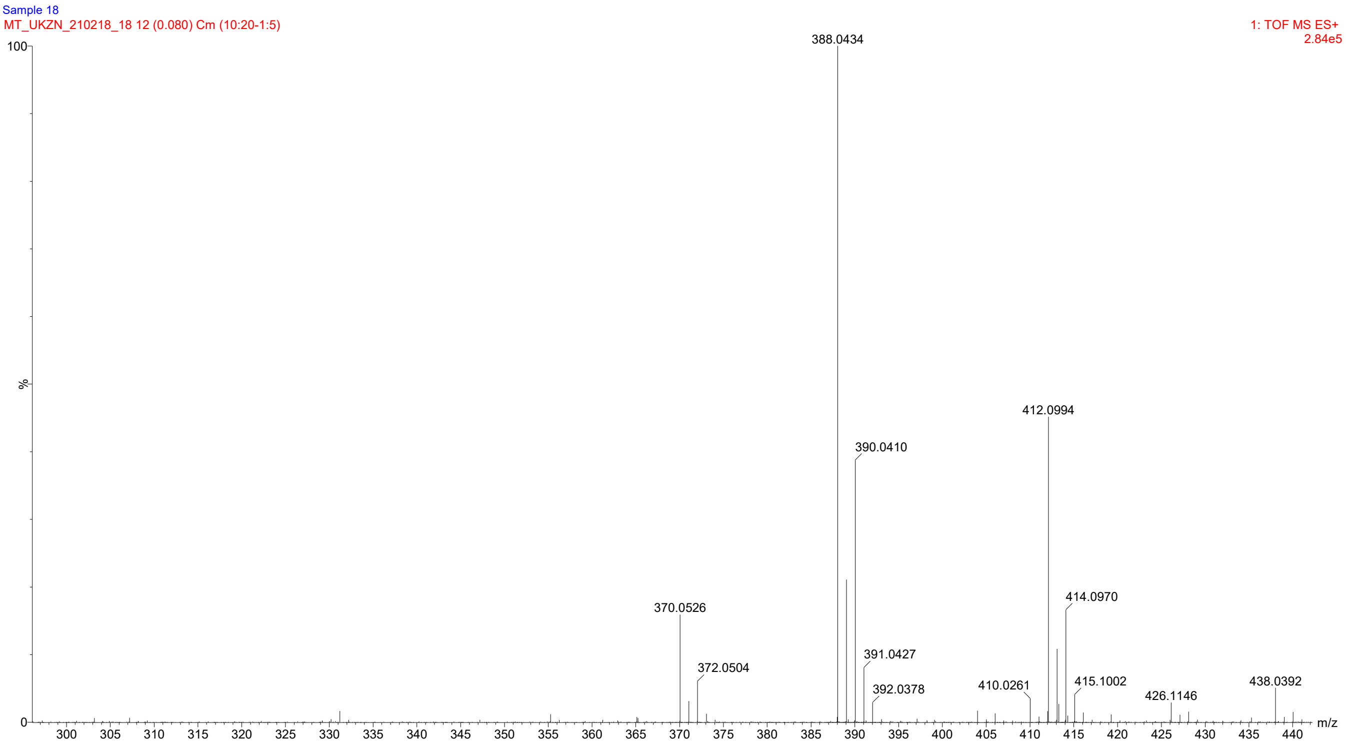

Figure S9: HRMS (ES+) for *2-(7-chloroquinolin-4-yl)-N-(6-fluorobenzo[d]thiazol-2-yl)hydrazine-1-carboxamide* (***6c***).

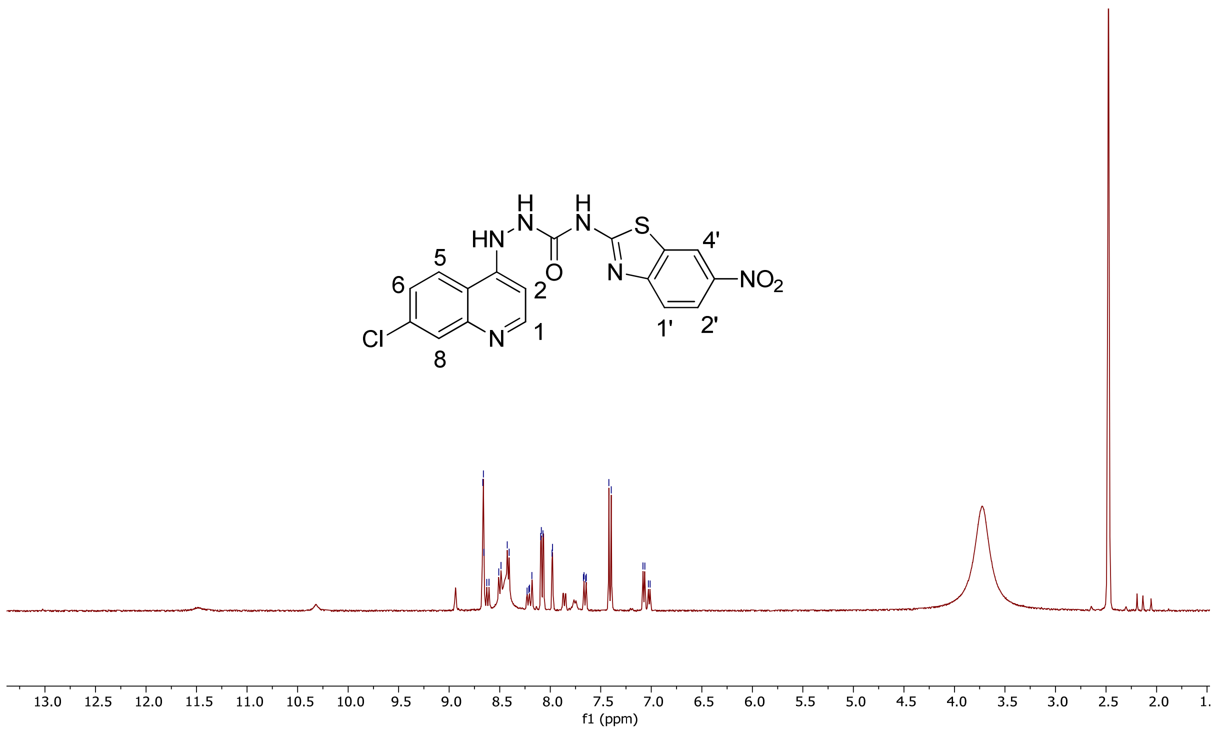

Figure S10: 400 MHz ^{1}H-NMR of *2-(7-chloroquinolin-4-yl)-N-(6-nitrobenzo[d]thiazol-2-yl)hydrazine-1-carboxamide* in DMSO-d6 ***(6d).***

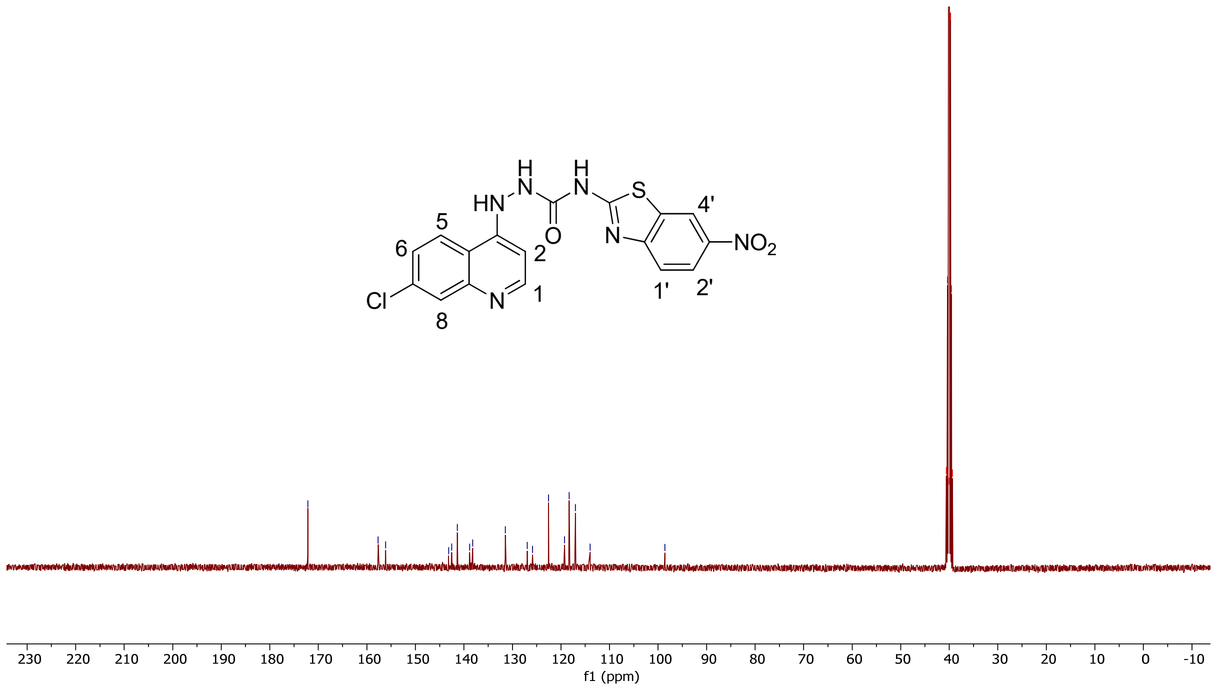

Figure S11: 101 MHz ^{13}C-NMR of *2-(7-chloroquinolin-4-yl)-N-(6-nitrobenzo[d]thiazol-2-yl)hydrazine-1-carboxamide* in DMSO-d6 ***(6d).***

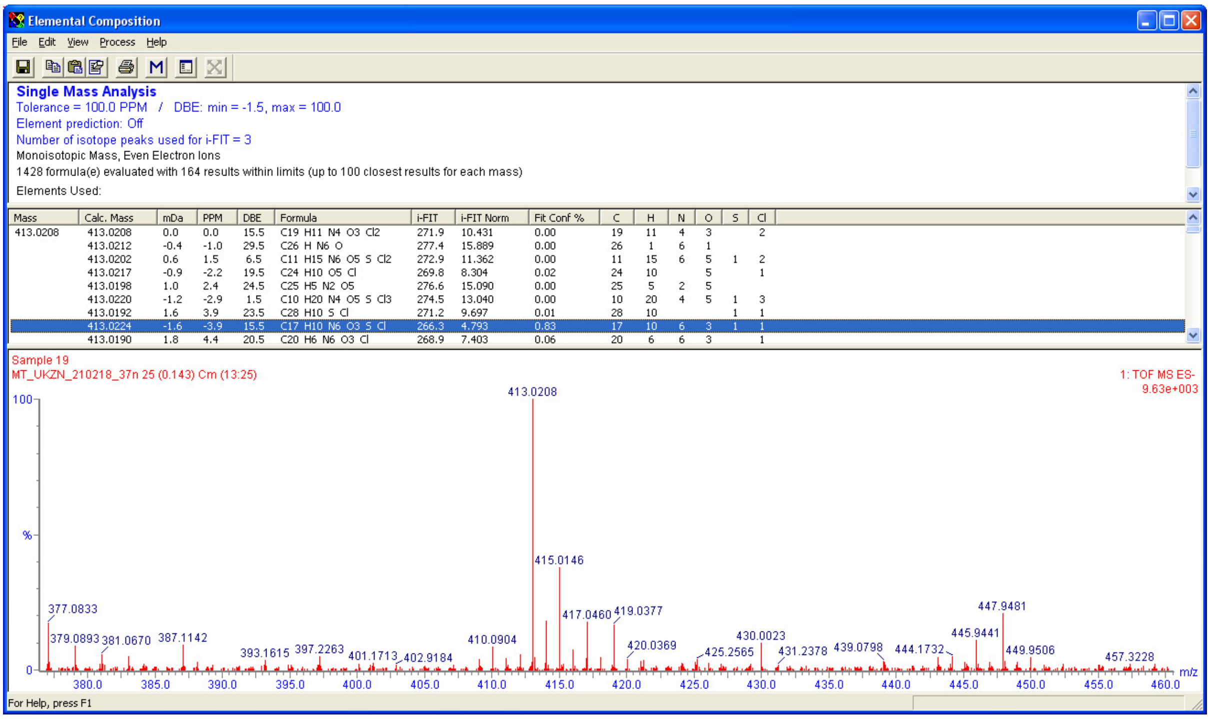

Single Mass Analysis
Tolerance = 100.0 PPM / DBE: min = -1.5, max = 100.0
Element prediction: Off
Number of isotope peaks used for i-FIT = 3
Monoisotopic Mass, Even Electron Ions
1428 formula(e) evaluated with 164 results within limits (up to 100 closest results for each mass)
Elements Used:

Mass	Calc. Mass	mDa	PPM	DBE	Formula	i-FIT	i-FIT Norm	Fit Conf %	C	H	N	O	S	Cl
413.0208	413.0208	0.0	0.0	15.5	C19 H11 N4 O3 Cl2	271.9	10.431	0.00	19	11	4	3		2
	413.0212	-0.4	-1.0	29.5	C26 H N6 O	277.4	15.889	0.00	26	1	6	1		
	413.0202	0.6	1.5	6.5	C11 H15 N6 O5 S Cl2	272.9	11.362	0.00	11	15	6	5	1	2
	413.0217	-0.9	-2.2	19.5	C24 H10 O5 Cl	269.8	8.304	0.02	24	10		5		1
	413.0198	1.0	2.4	24.5	C25 H5 N2 O5	276.6	15.090	0.00	25	5	2	5		
	413.0220	-1.2	-2.9	1.5	C10 H20 N4 O5 S Cl3	274.5	13.040	0.00	10	20	4	5	1	3
	413.0192	1.6	3.9	23.5	C28 H10 S Cl	271.2	9.697	0.01	28	10			1	1
	413.0224	-1.6	-3.9	15.5	C17 H10 N6 O3 S Cl	266.3	4.793	0.83	17	10	6	3	1	1
	413.0190	1.8	4.4	20.5	C20 H6 N6 O3 Cl	268.9	7.403	0.06	20	6	6	3		1

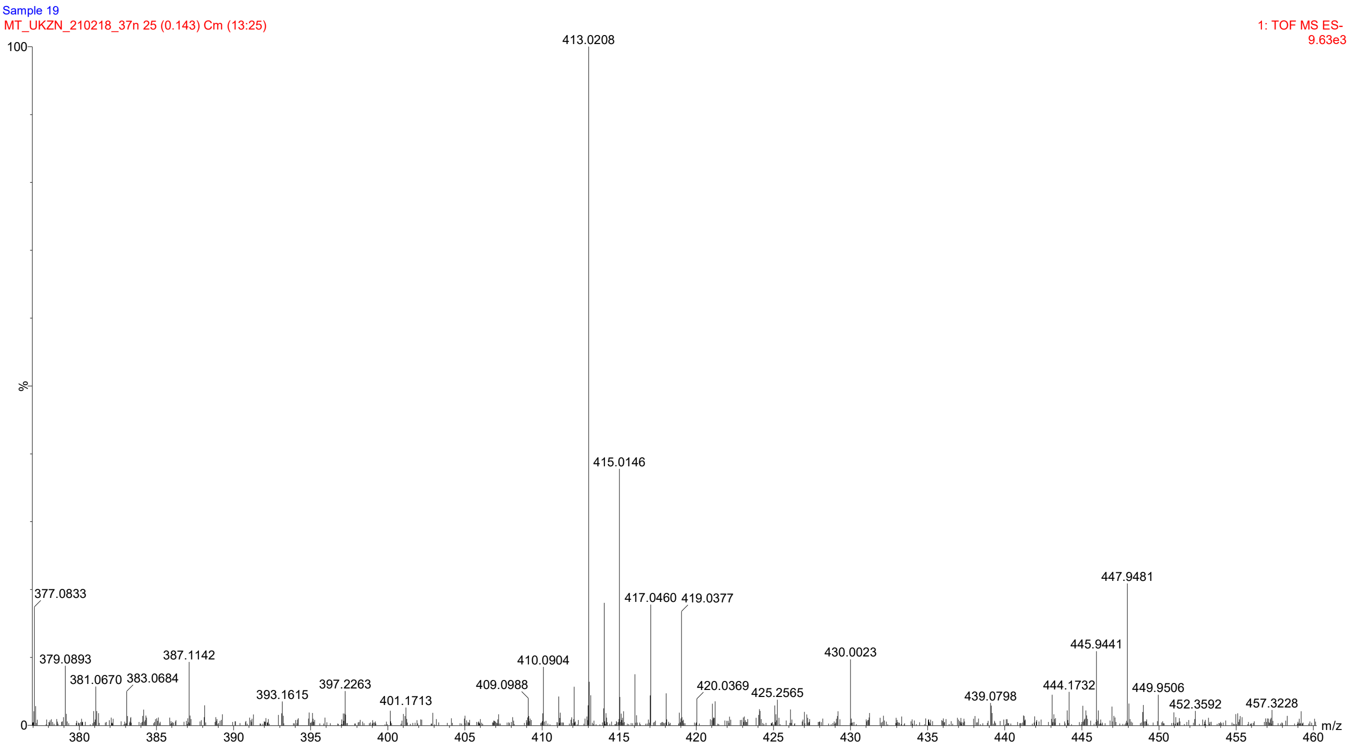

Figure S12: HRMS (ES^{-}) for *2-(7-chloroquinolin-4-yl)-N-(6-nitrobenzo[d]thiazol-2-yl)hydrazine-1-carboxamide* (**6d**) *(negative ion mode)*.

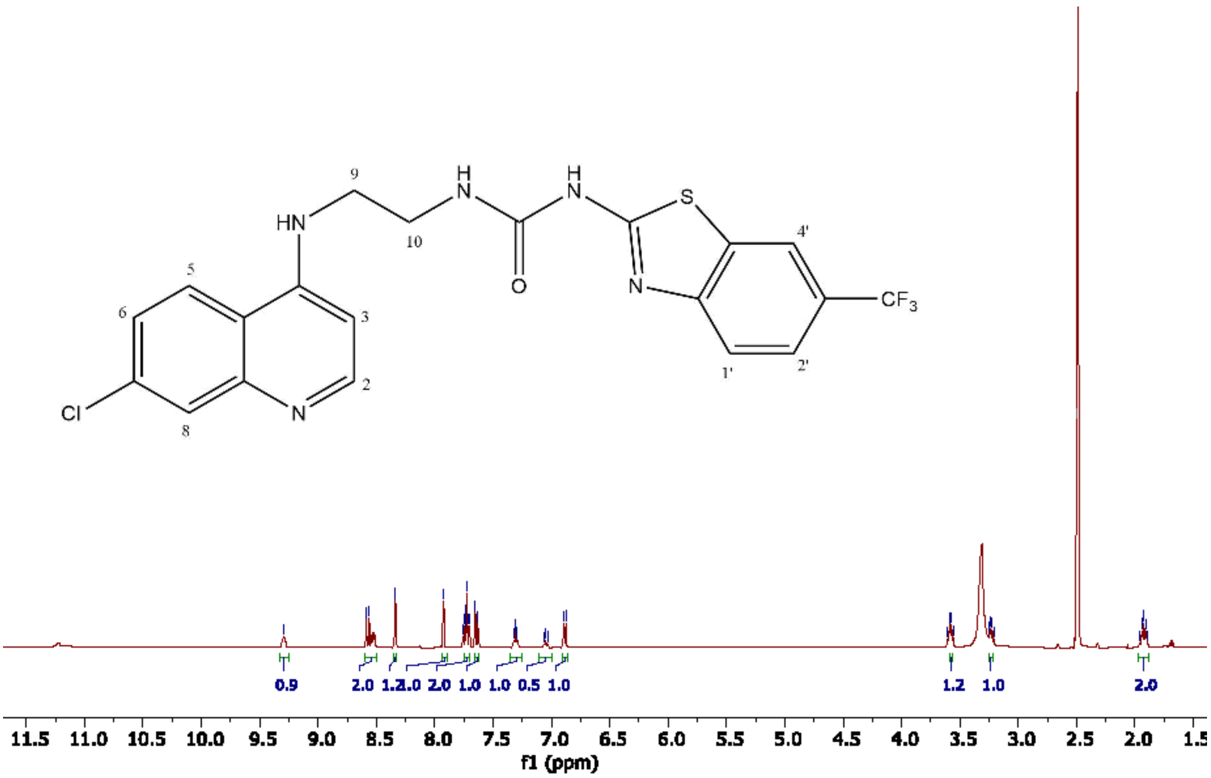

Figure S13: 400 MHz ^{1}H-NMR of *1-(2-((7-chloroquinolin-4-yl)amino)ethyl)-3-(6-(trifluoromethyl)benzo[d]thiazol-2-yl)urea* in DMSO-d6 (***6e***).

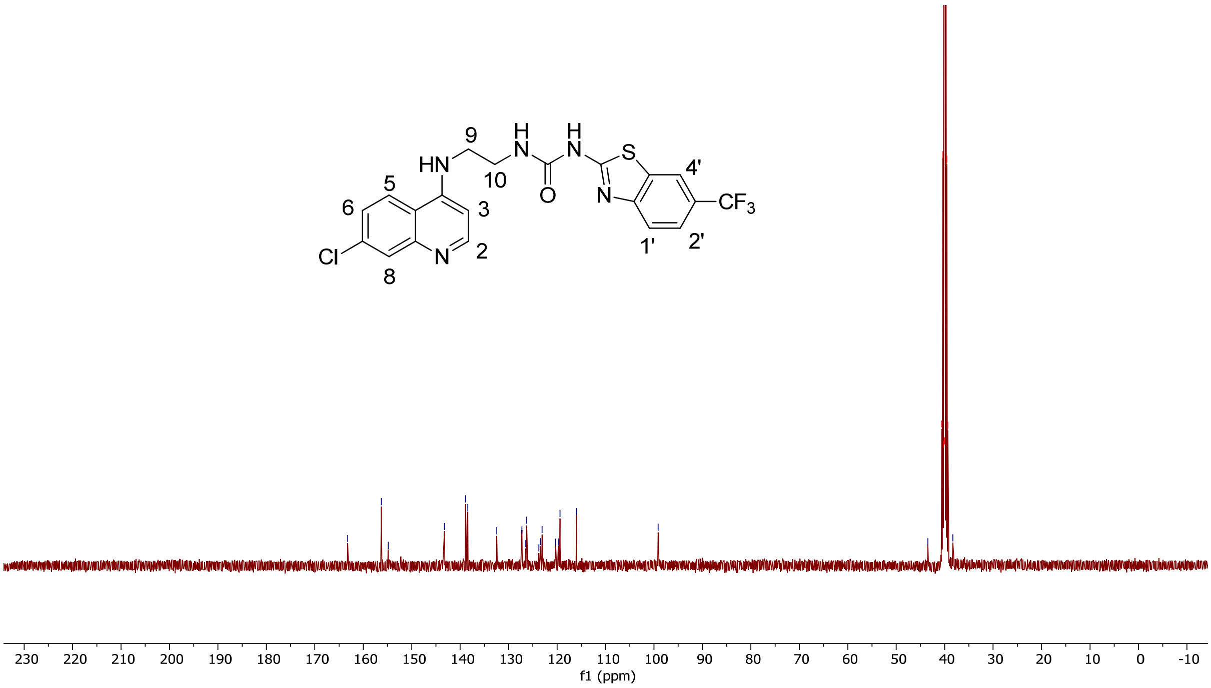

Figure S14: 101 MHz ^{13}C-NMR of *1-(2-((7-chloroquinolin-4-yl)amino)ethyl)-3-(6-(trifluoromethyl)benzo[d]thiazol-2-yl)urea* in DMSO-d6 (***6e***).

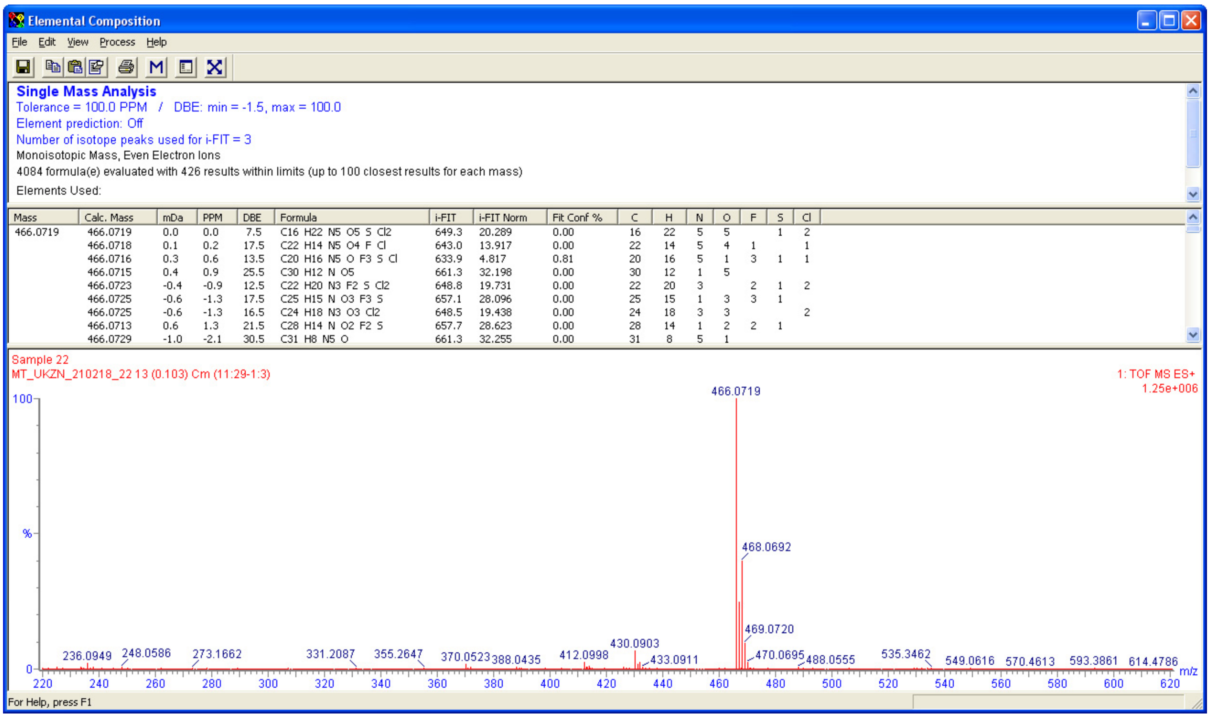

Elemental Composition

Single Mass Analysis
Tolerance = 100.0 PPM / DBE: min = -1.5, max = 100.0
Element prediction: Off
Number of isotope peaks used for i-FIT = 3
Monoisotopic Mass, Even Electron Ions
4084 formula(e) evaluated with 426 results within limits (up to 100 closest results for each mass)
Elements Used:

Mass	Calc. Mass	mDa	PPM	DBE	Formula	i-FIT	i-FIT Norm	Fit Conf %	C	H	N	O	F	S	Cl
466.0719	466.0719	0.0	0.0	7.5	C16 H22 N5 O5 S Cl2	649.3	20.289	0.00	16	22	5	5		1	2
	466.0718	0.1	0.2	17.5	C22 H14 N5 O4 F Cl	643.0	13.917	0.00	22	14	5	4	1		1
	466.0716	0.3	0.6	13.5	C20 H16 N5 O F3 S Cl	633.9	4.817	0.81	20	16	5	1	3	1	1
	466.0715	0.4	0.9	25.5	C30 H12 N O5	661.3	32.198	0.00	30	12	1	5			
	466.0723	-0.4	-0.9	12.5	C22 H20 N3 F2 S Cl2	648.8	19.731	0.00	22	20	3		2	1	2
	466.0725	-0.6	-1.3	17.5	C25 H15 N O3 F3 S	657.1	28.096	0.00	25	15	1	3	3	1	
	466.0725	-0.6	-1.3	16.5	C24 H18 N3 O3 Cl2	648.5	19.438	0.00	24	18	3	3			2
	466.0713	0.6	1.3	21.5	C28 H14 N O2 F2 S	657.7	28.623	0.00	28	14	1	2	2	1	
	466.0729	-1.0	-2.1	30.5	C31 H8 N5 O	661.3	32.255	0.00	31	8	5	1			

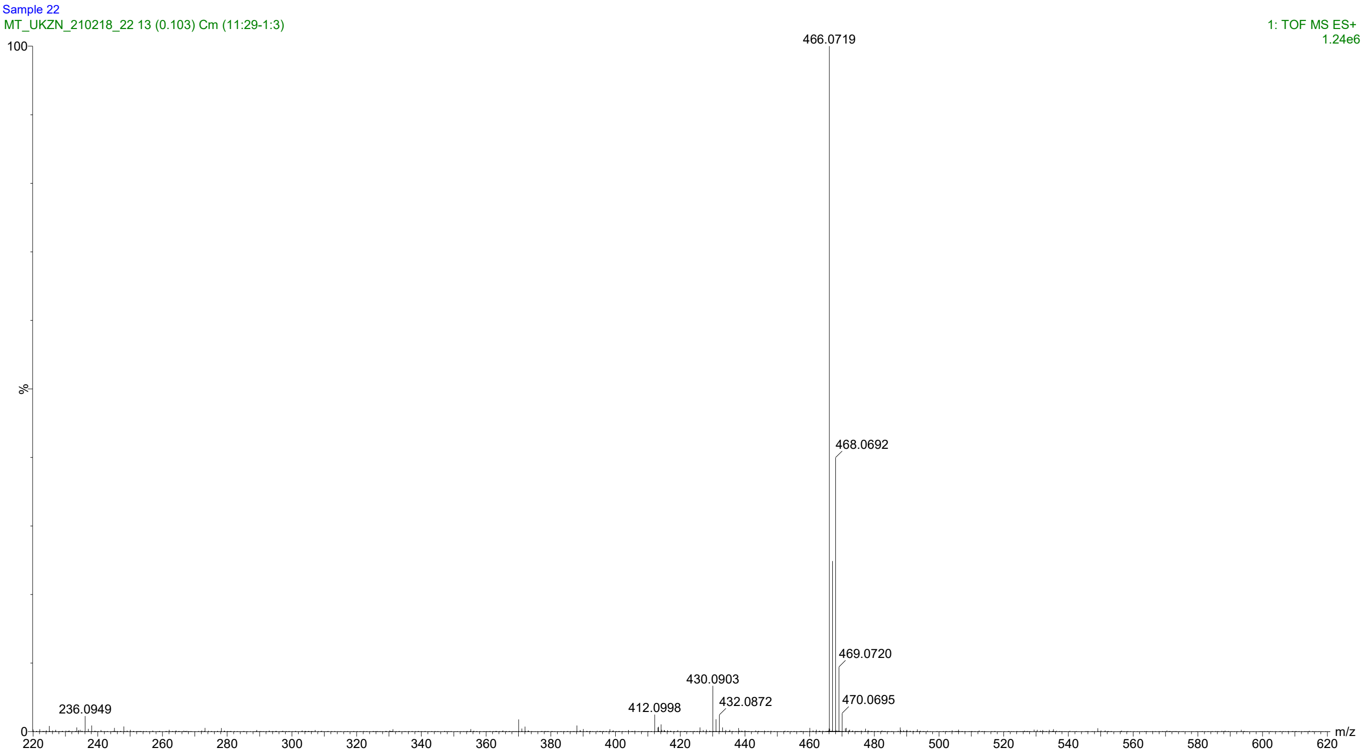

Figure S15: HRMS (ES^+) *1-(2-((7-chloroquinolin-4-yl)amino)ethyl)-3-(6-(trifluoromethyl)benzo[d]thiazol-2-yl)urea* (**6e**).

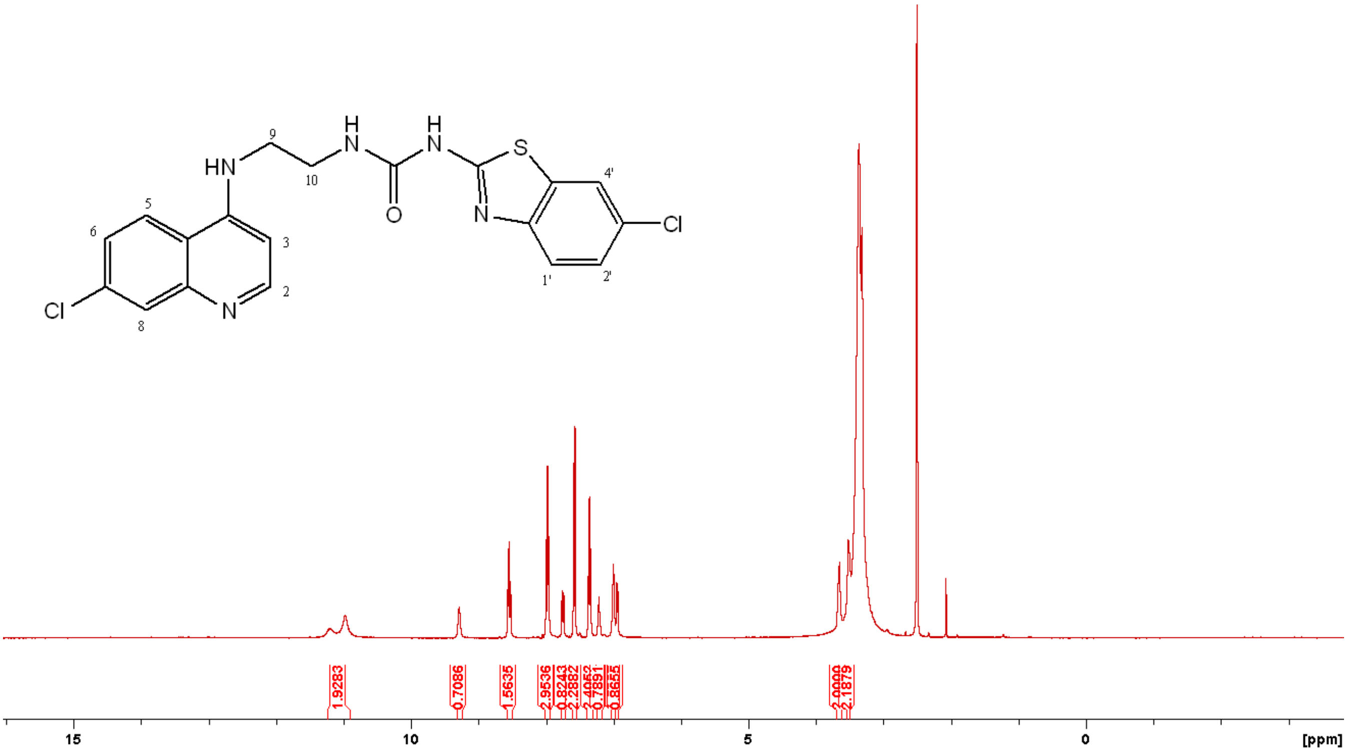

Figure S16: 400 MHz ^{1}H-NMR of *1-(6-chlorobenzo[d]thiazol-2-yl)-3-(2-((7-chloroquinolin-4-yl)amino)ethyl)urea* in DMSO-d6 (***6f***).

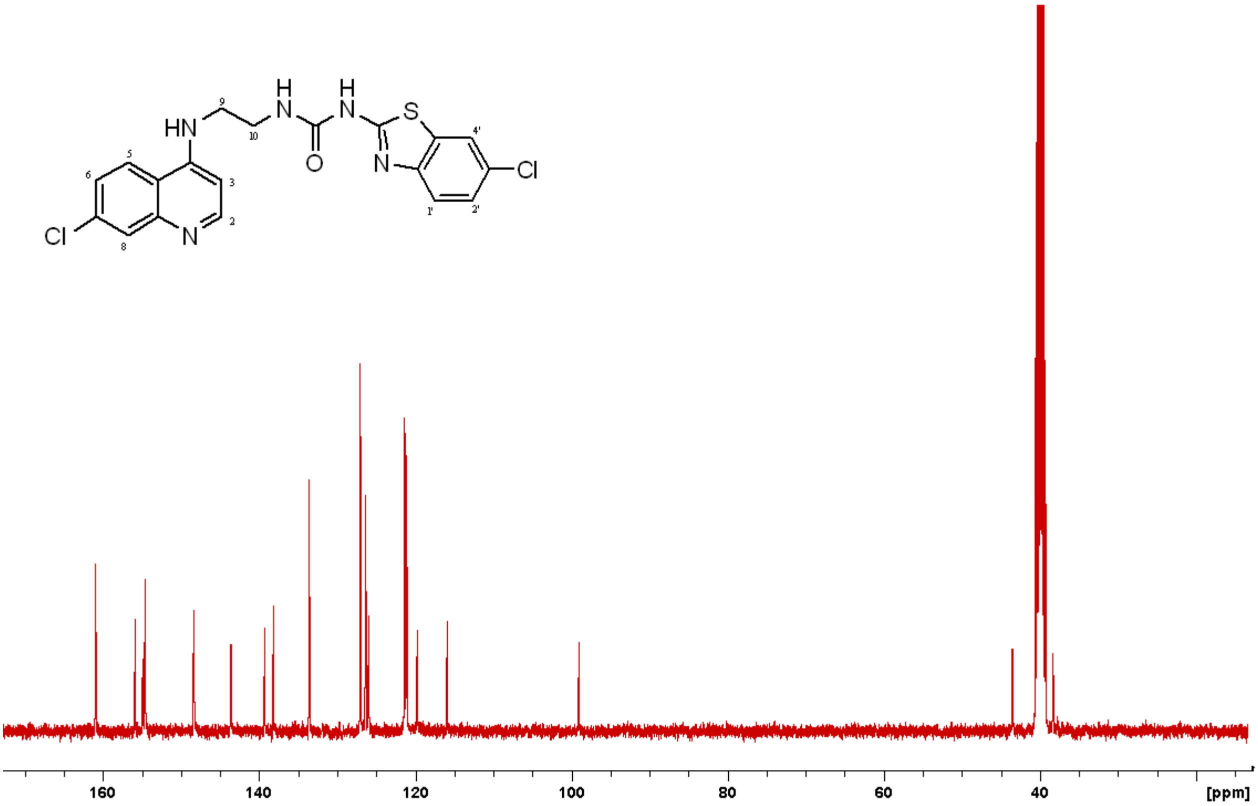

Figure S17: 101 MHz ^{13}C-NMR of *1-(6-chlorobenzo[d]thiazol-2-yl)-3-(2-((7-chloroquinolin-4-yl)amino)ethyl)urea* in DMSO-d6 (***6f***).

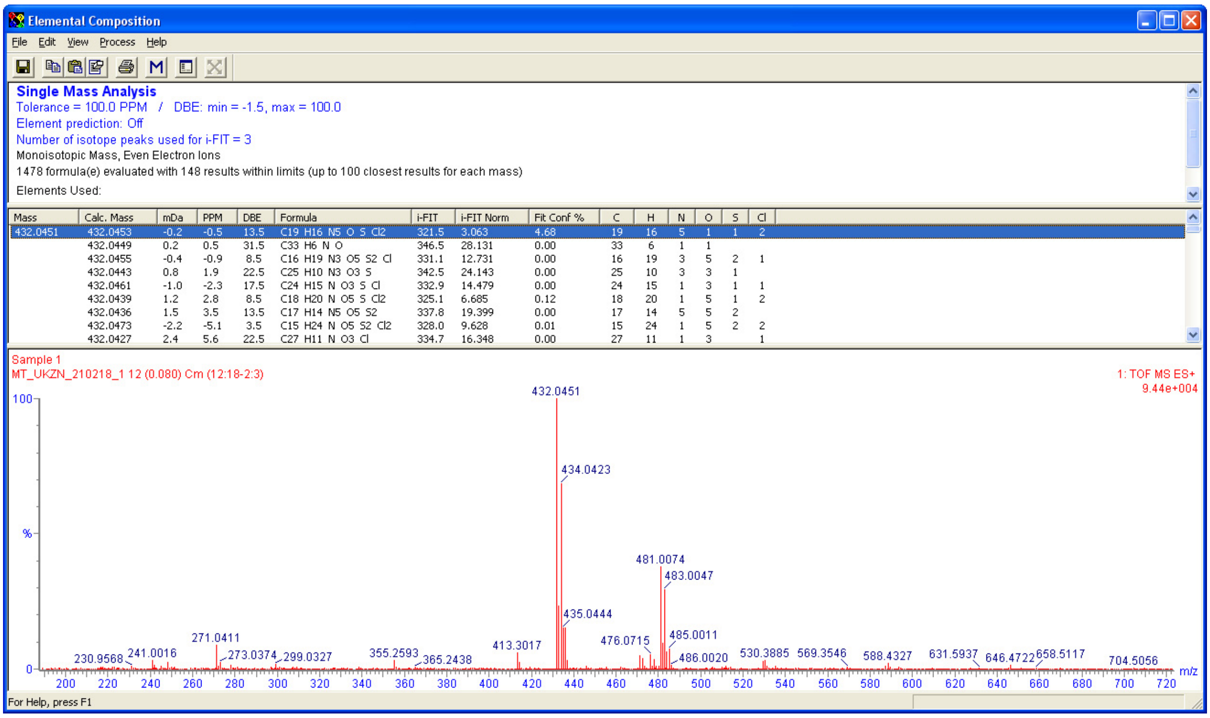

Mass	Calc. Mass	mDa	PPM	DBE	Formula	i-FIT	i-FIT Norm	Fit Conf %	C	H	N	O	S	Cl
432.0451	432.0453	-0.2	-0.5	13.5	C19 H16 N5 O S Cl2	321.5	3.063	4.68	19	16	5	1	1	2
	432.0449	0.2	0.5	31.5	C33 H6 N O	346.5	28.131	0.00	33	6	1	1		
	432.0455	-0.4	-0.9	8.5	C16 H19 N3 O5 S2 Cl	331.1	12.731	0.00	16	19	3	5	2	1
	432.0443	0.8	1.9	22.5	C25 H10 N3 O3 S	342.5	24.143	0.00	25	10	3	3	1	
	432.0461	-1.0	-2.3	17.5	C24 H15 N O3 S Cl	332.9	14.479	0.00	24	15	1	3	1	1
	432.0439	1.2	2.8	8.5	C18 H20 N O5 S Cl2	325.1	6.685	0.12	18	20	1	5	1	2
	432.0436	1.5	3.5	13.5	C17 H14 N5 O5 S2	337.8	19.399	0.00	17	14	5	5	2	
	432.0473	-2.2	-5.1	3.5	C15 H24 N O5 S2 Cl2	328.0	9.628	0.01	15	24	1	5	2	2
	432.0427	2.4	5.6	22.5	C27 H11 N O3 Cl	334.7	16.348	0.00	27	11	1	3		1

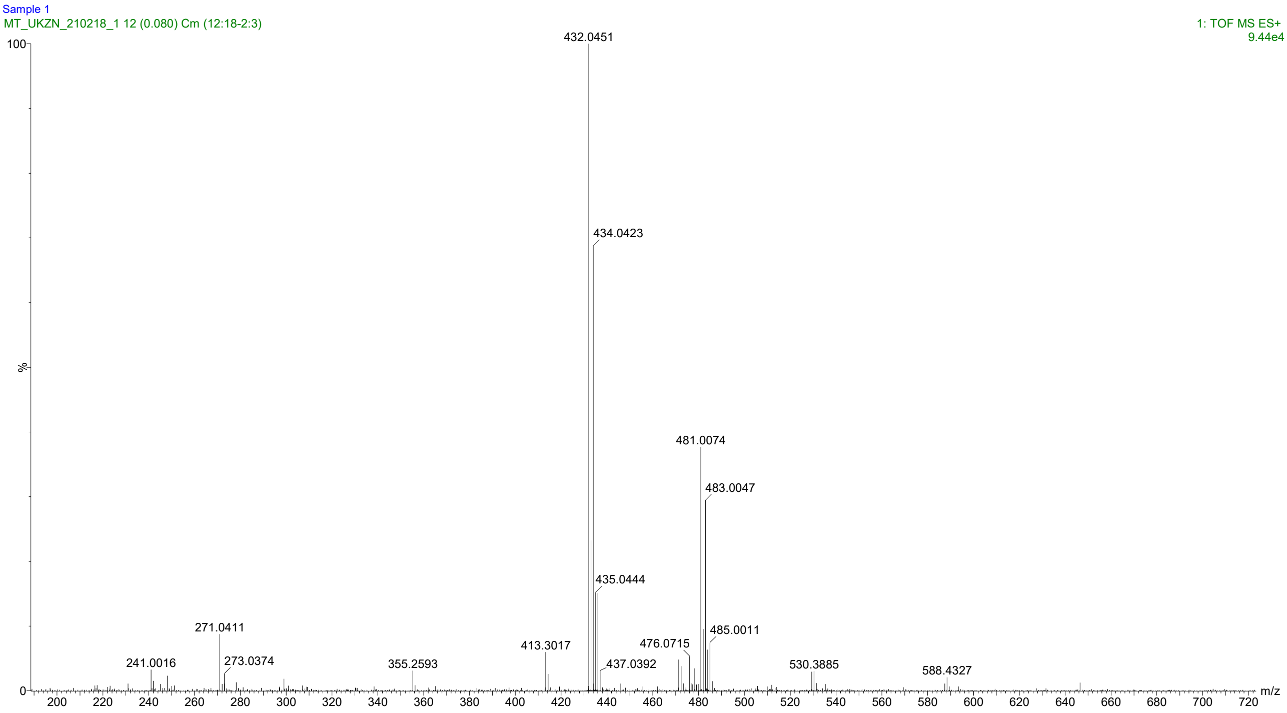

Figure S18: HRMS (ES$^+$) for *1-(6-chlorobenzo[d]thiazol-2-yl)-3-(2-((7-chloroquinolin-4-yl)amino)ethyl)urea* (***6f***).

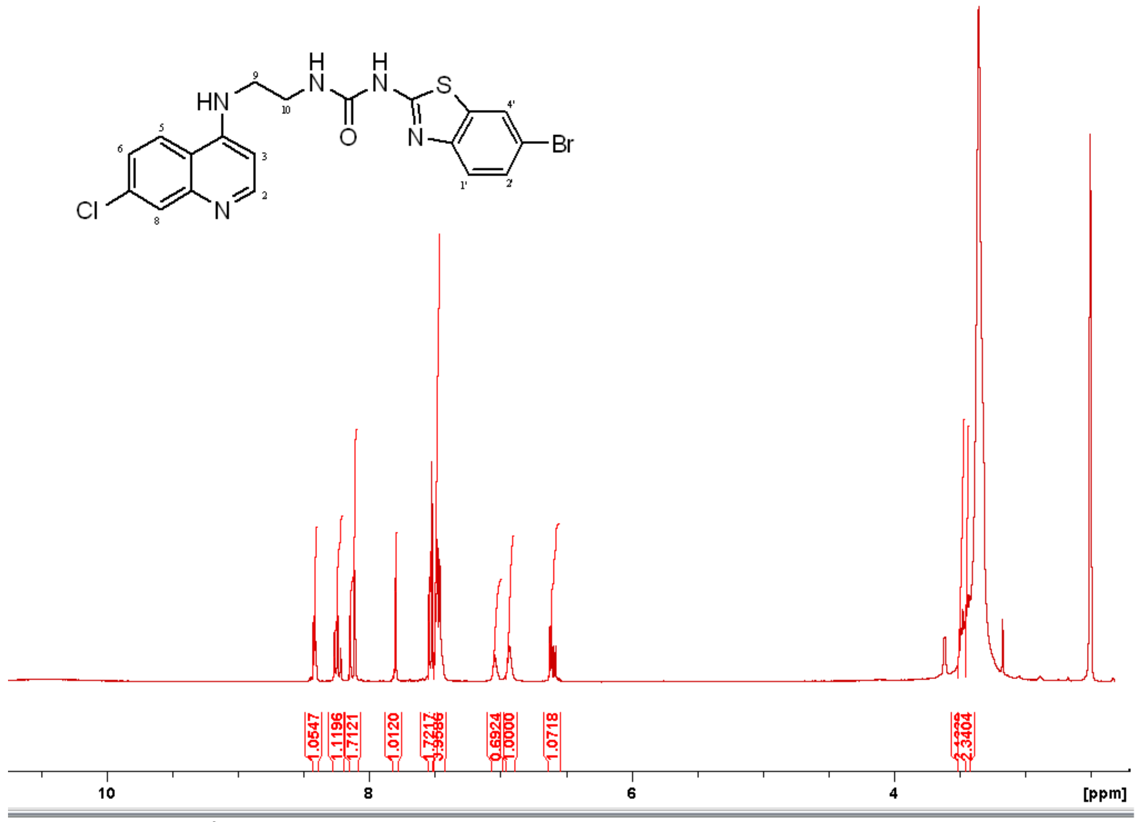

Figure S19: ^{1}H-NMR of *1-(6-bromobenzo[d]thiazol-2-yl)-3-(2-((7-chloroquinolin-4-yl)amino)ethyl)urea* in DMSO-d6 *(**6g**)*.

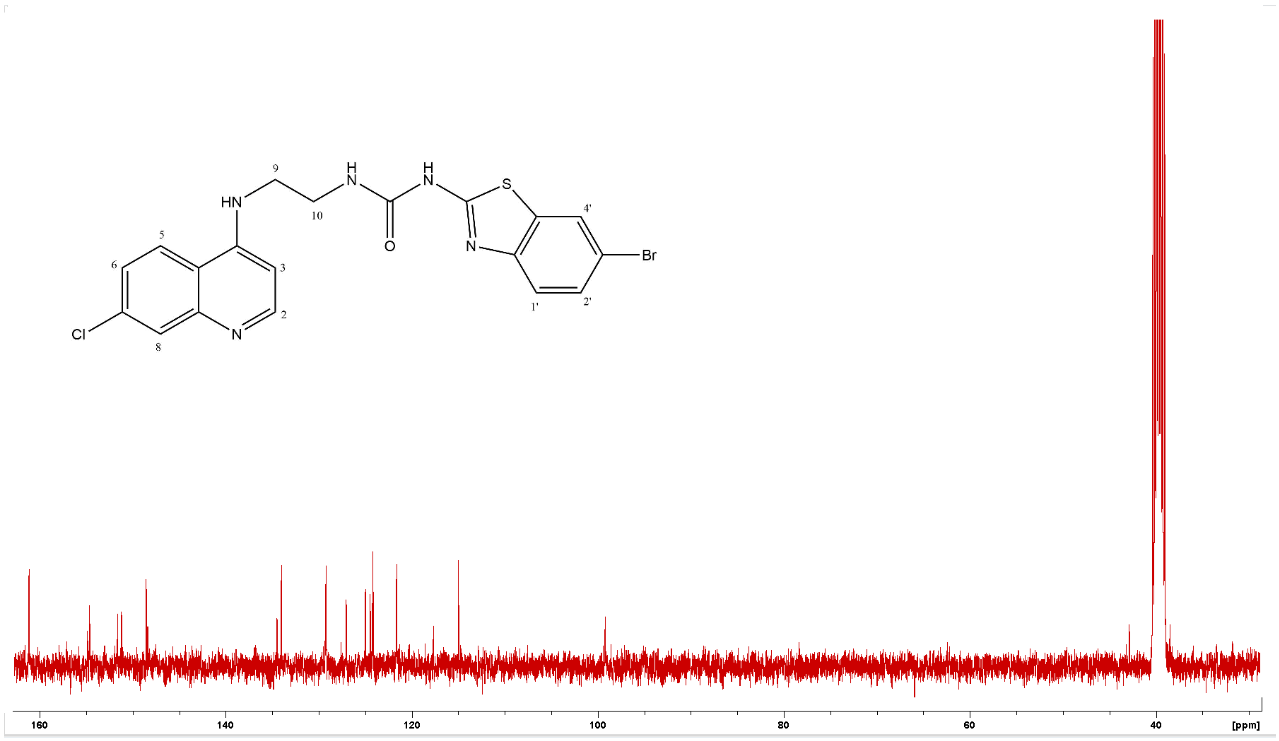

Figure S20: ^{13}C NMR (APT) of *1-(6-bromobenzo[d]thiazol-2-yl)-3-(2-((7-chloroquinolin-4-yl)amino)ethyl)urea* in DMSO-d6 (***6g***).

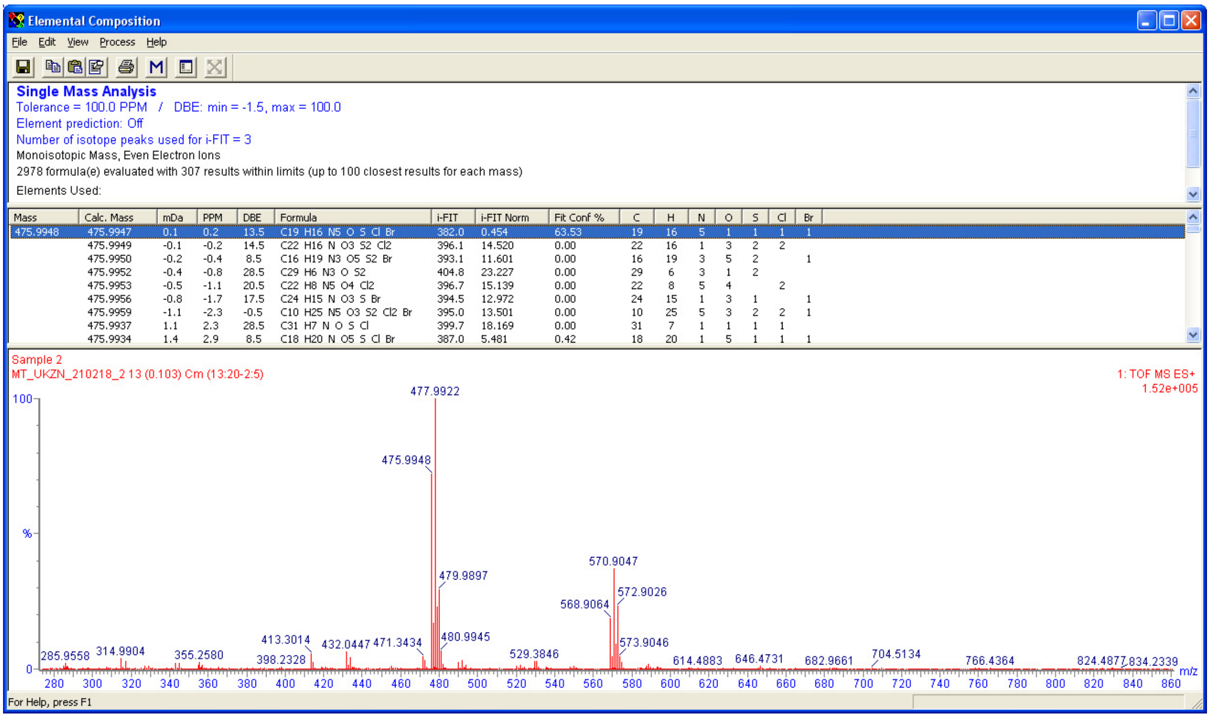

Elemental Composition

File Edit View Process Help

Single Mass Analysis

Tolerance = 100.0 PPM / DBE: min = -1.5, max = 100.0

Element prediction: Off

Number of isotope peaks used for i-FIT = 3

Monoisotopic Mass, Even Electron Ions

2978 formula(e) evaluated with 307 results within limits (up to 100 closest results for each mass)

Elements Used:

Mass	Calc. Mass	mDa	PPM	DBE	Formula	i-FIT	i-FIT Norm	Fit Conf %	C	H	N	O	S	Cl	Br
475.9948	475.9947	0.1	0.2	13.5	C19 H16 N5 O S Cl Br	382.0	0.454	63.53	19	16	5	1	1	1	1
	475.9949	-0.1	-0.2	14.5	C22 H16 N O3 S2 Cl2	396.1	14.520	0.00	22	16	1	3	2	2	
	475.9950	-0.2	-0.4	8.5	C16 H19 N3 O5 S2 Br	393.1	11.601	0.00	16	19	3	5	2		1
	475.9952	-0.4	-0.8	28.5	C29 H6 N3 O S2	404.8	23.227	0.00	29	6	3	1	2		
	475.9953	-0.5	-1.1	20.5	C22 H8 N5 O4 Cl2	396.7	15.139	0.00	22	8	5	4		2	
	475.9956	-0.8	-1.7	17.5	C24 H15 N O3 S Br	394.5	12.972	0.00	24	15	1	3	1		1
	475.9959	-1.1	-2.3	-0.5	C10 H25 N5 O3 S2 Cl2 Br	395.0	13.501	0.00	10	25	5	3	2	2	1
	475.9937	1.1	2.3	28.5	C31 H7 N O S Cl	399.7	18.169	0.00	31	7	1	1	1	1	
	475.9934	1.4	2.9	8.5	C18 H20 N O5 S Cl Br	387.0	5.481	0.42	18	20	1	5	1	1	1

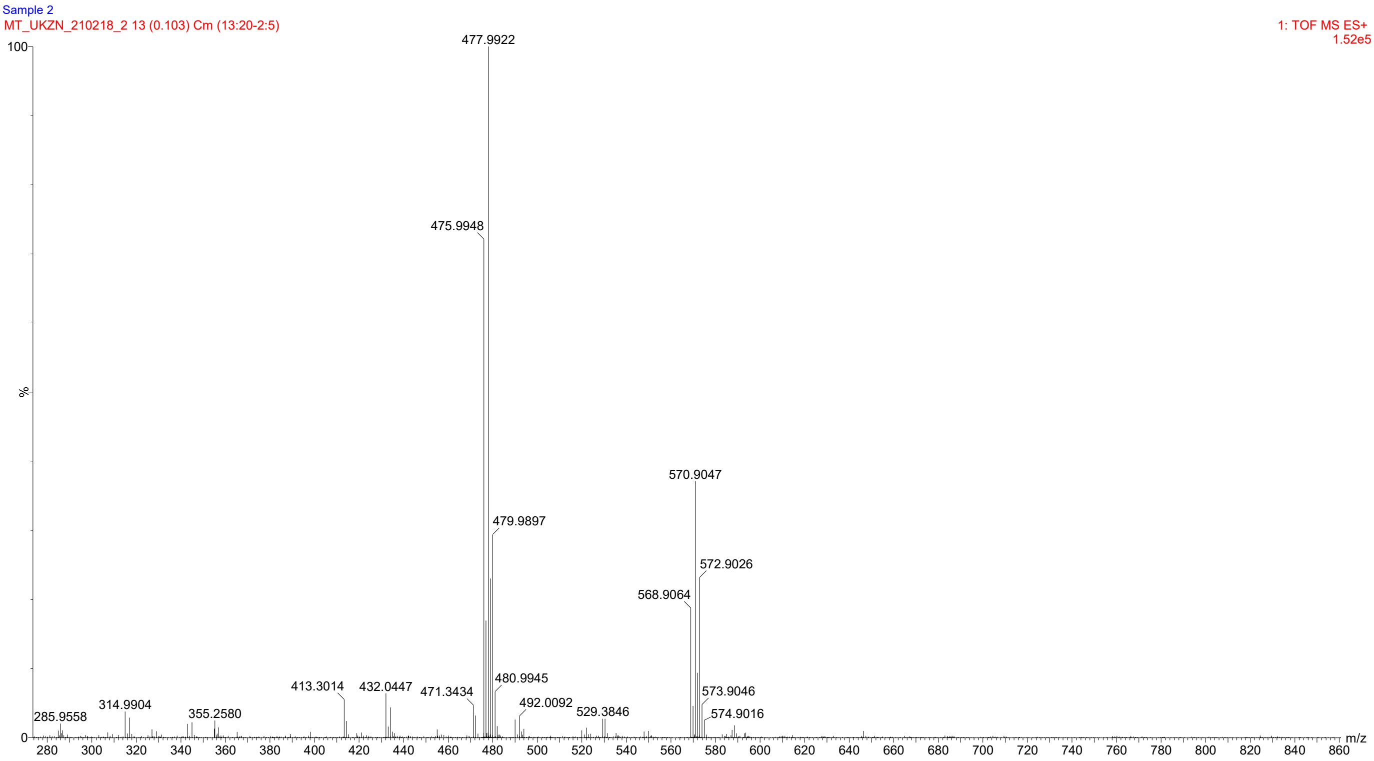

Figure S21:HRMS (ES^{+}) for *1-(6-bromobenzo[d]thiazol-2-yl)-3-(2-((7-chloroquinolin-4-yl)amino)ethyl)urea* (**6g**).

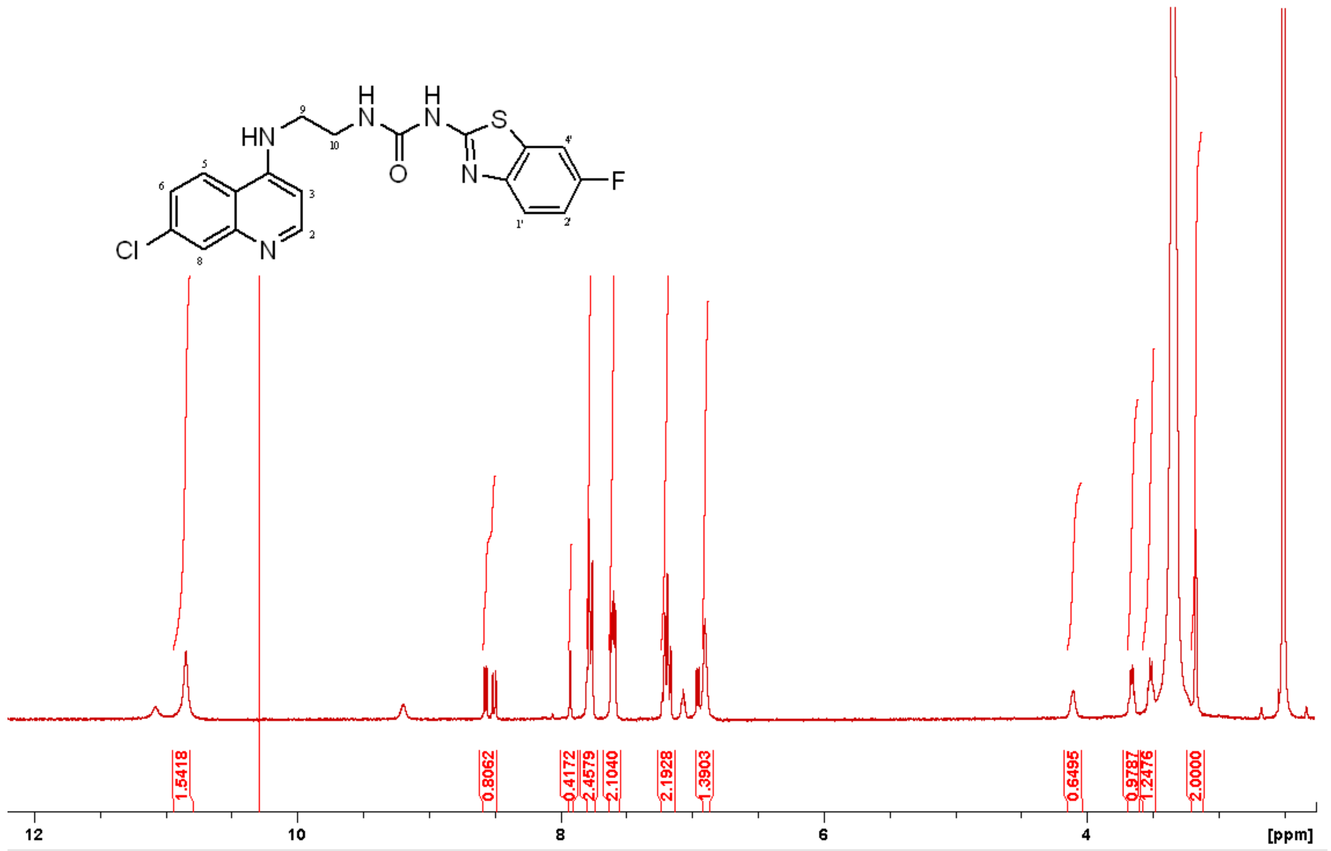

Figure S22: 400 MHz ^{1}H-NMR of *1-(6-flourobenzo[d]thiazol-2-yl)-3-(2-((7-chloroquinolin-4-yl)amino)ethyl)urea* in DMSO-d6 ***(6h)***.

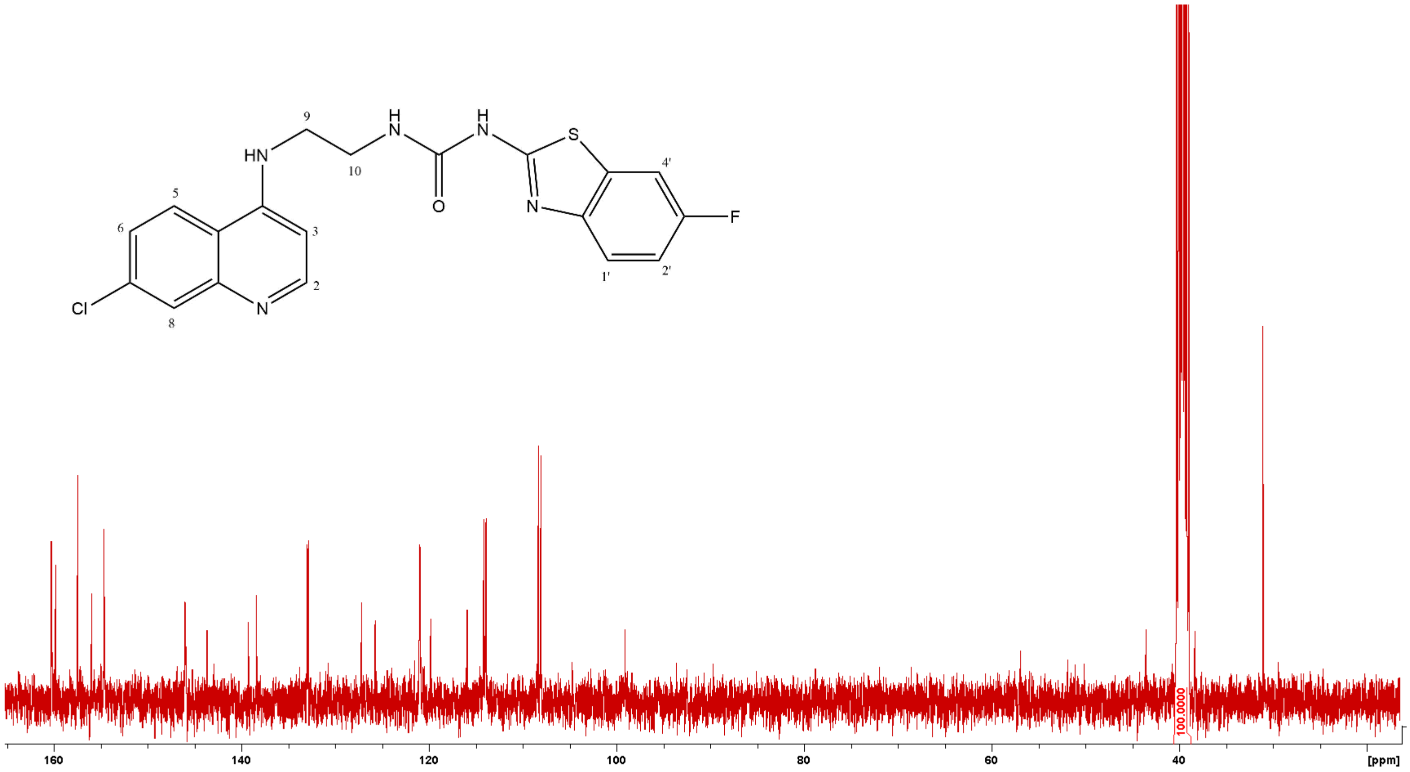

Figure S23: 101 MHz ^{13}C-NMR of *1-(6-flourobenzo[d]thiazol-2-yl)-3-(2-((7-chloroquinolin-4-yl)amino)ethyl)urea* in DMSO-d6 ***(6h)***.

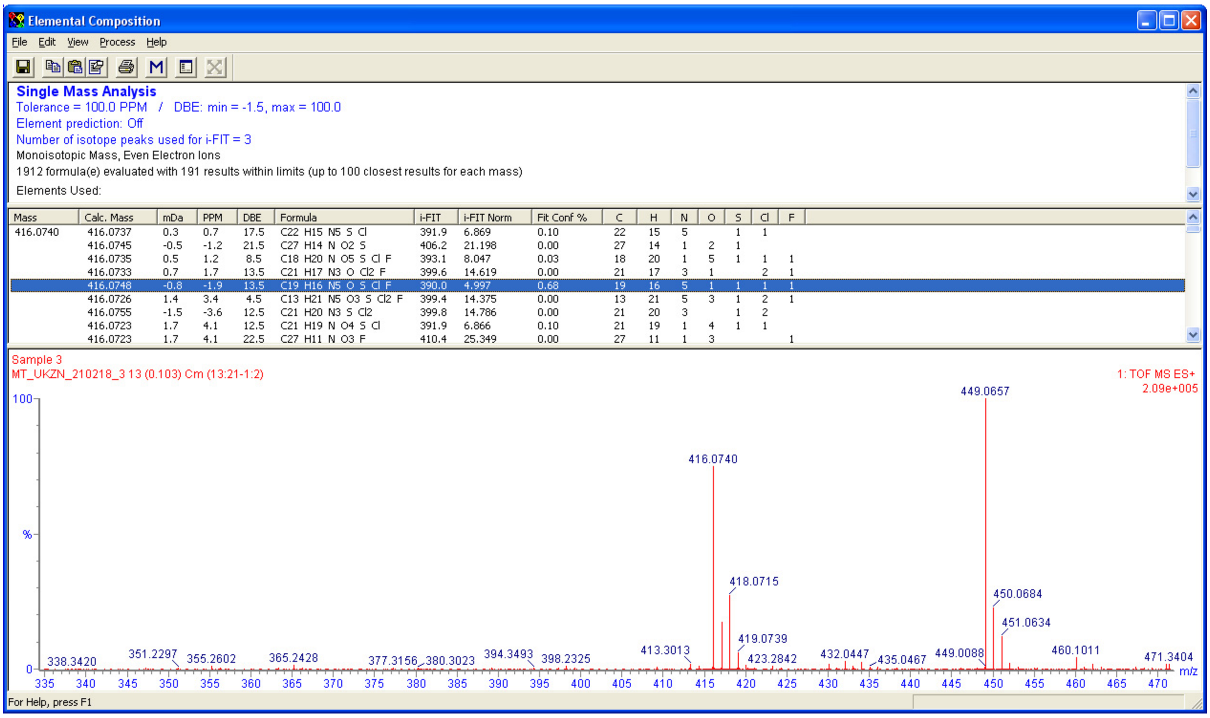

Mass	Calc. Mass	mDa	PPM	DBE	Formula	i-FIT	i-FIT Norm	Fit Conf %	C	H	N	O	S	Cl	F
416.0740	416.0737	0.3	0.7	17.5	C22 H15 N5 S Cl	391.9	6.869	0.10	22	15	5		1	1	
	416.0745	-0.5	-1.2	21.5	C27 H14 N O2 S	406.2	21.198	0.00	27	14	1	2	1		
	416.0735	0.5	1.2	8.5	C18 H20 N O5 S Cl F	393.1	8.047	0.03	18	20	1	5	1	1	1
	416.0733	0.7	1.7	13.5	C21 H17 N3 O Cl2 F	399.6	14.619	0.00	21	17	3	1		2	1
	416.0748	-0.8	-1.9	13.5	C19 H16 N5 O S Cl F	390.0	4.997	0.68	19	16	5	1	1	1	1
	416.0726	1.4	3.4	4.5	C13 H21 N5 O3 S Cl2 F	399.4	14.375	0.00	13	21	5	3	1	2	1
	416.0755	-1.5	-3.6	12.5	C21 H20 N3 S Cl2	399.8	14.786	0.00	21	20	3		1	2	
	416.0723	1.7	4.1	12.5	C21 H19 N O4 S Cl	391.9	6.866	0.10	21	19	1	4	1	1	
	416.0723	1.7	4.1	22.5	C27 H11 N O3 F	410.4	25.349	0.00	27	11	1	3			1

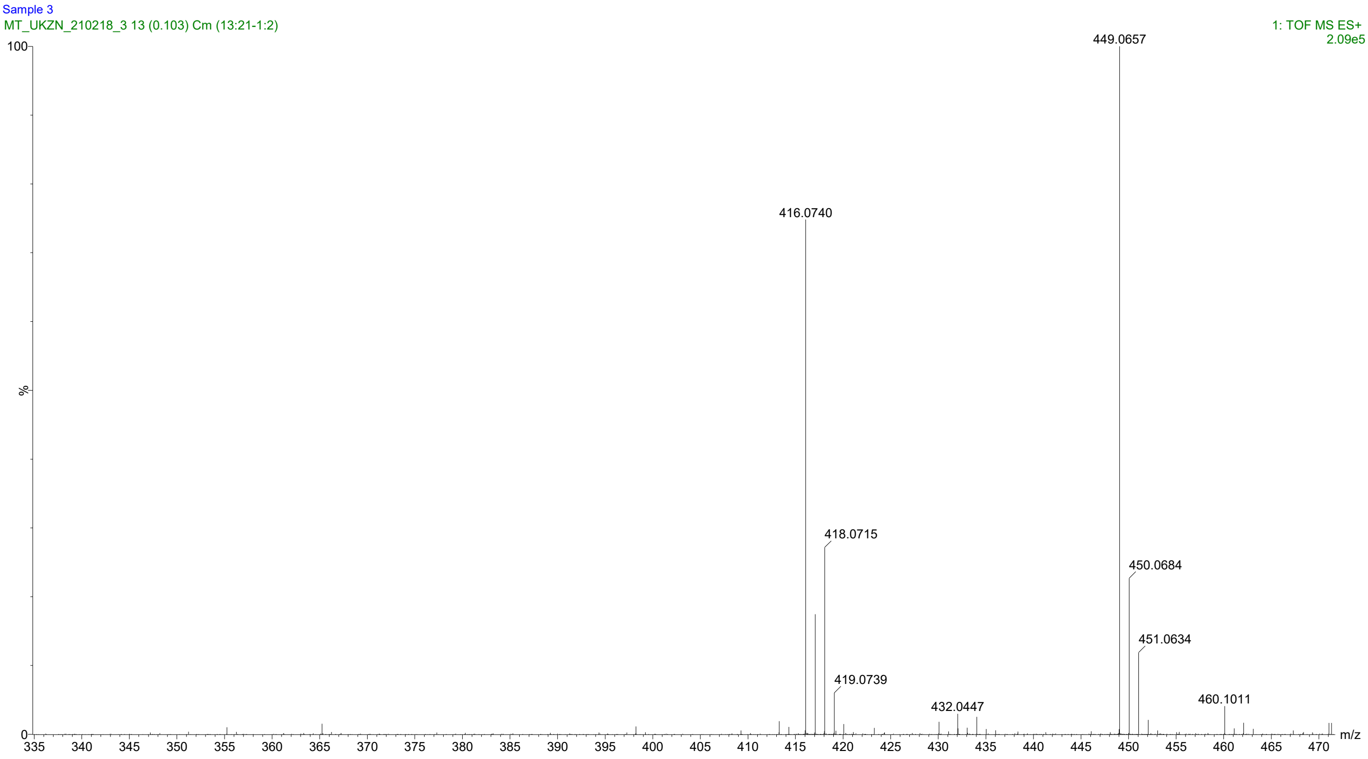

Figure S24: HRMS (ES$^+$) for *1-(6-fluorobenzo[d]thiazol-2-yl)-3-(2-((7-chloroquinolin-4-yl)amino)ethyl)urea (**6h**).*

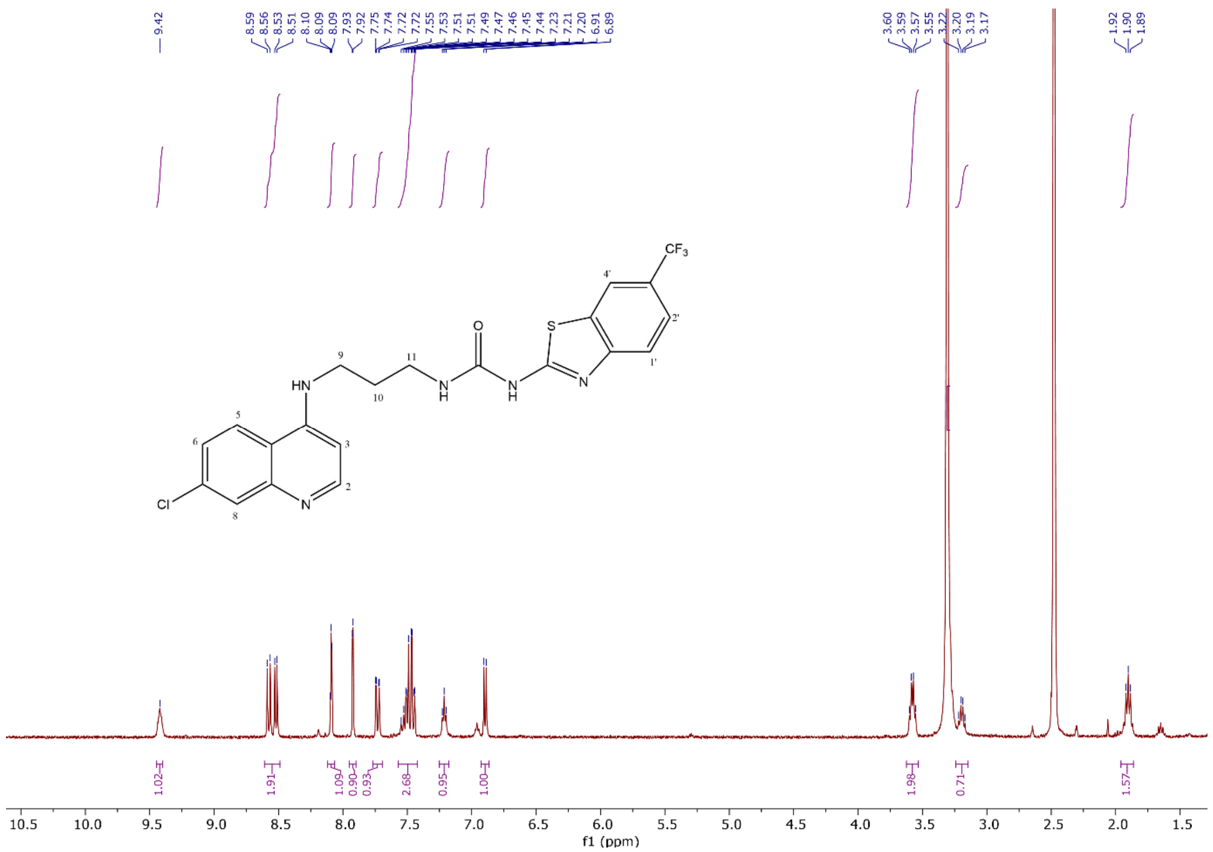

Figure S25: 400 MHz ^{1}H-NMR of *1-(3-((7-chloroquinolin-4-yl)amino)propyl)-3-(6-(trifluoromethyl)benzo[d]thiazol-2-yl)urea* in DMSO-d6 (***6i***).

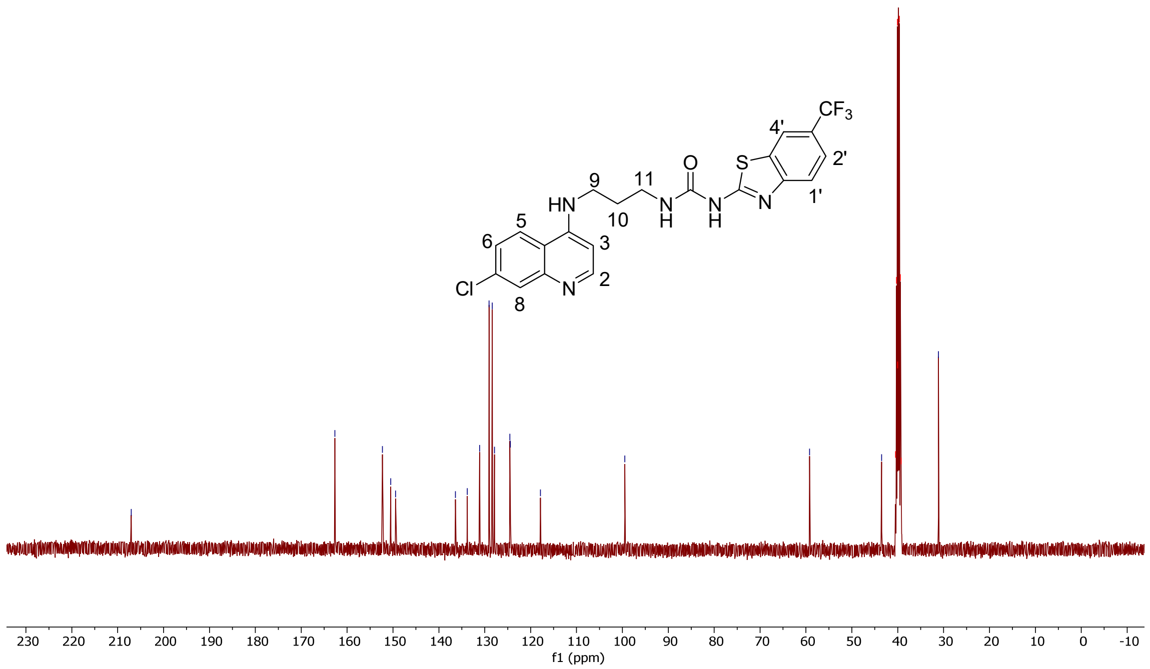

Figure S26: 101 MHz ^{13}C-NMR of *1-(3-((7-chloroquinolin-4-yl)amino)propyl)-3-(6-(trifluoromethyl)benzo[d]thiazol-2-yl)urea* in DMSO-d6 (***6i***).

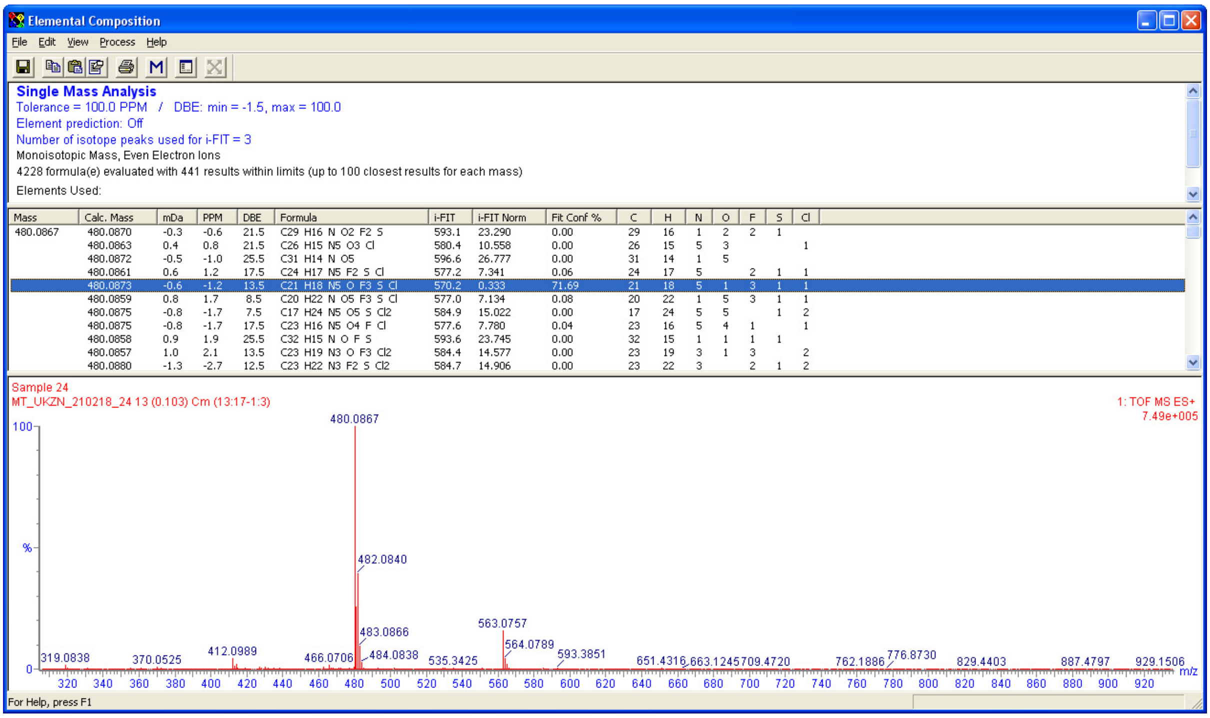

Single Mass Analysis
Tolerance = 100.0 PPM / DBE: min = -1.5, max = 100.0
Element prediction: Off
Number of isotope peaks used for i-FIT = 3
Monoisotopic Mass, Even Electron Ions
4228 formula(e) evaluated with 441 results within limits (up to 100 closest results for each mass)
Elements Used:

Mass	Calc. Mass	mDa	PPM	DBE	Formula	i-FIT	i-FIT Norm	Fit Conf %	C	H	N	O	F	S	Cl
480.0867	480.0870	-0.3	-0.6	21.5	C29 H16 N O2 F2 S	593.1	23.290	0.00	29	16	1	2	2	1	
	480.0863	0.4	0.8	21.5	C26 H15 N5 O3 Cl	580.4	10.558	0.00	26	15	5	3			1
	480.0872	-0.5	-1.0	25.5	C31 H14 N O5	596.6	26.777	0.00	31	14	1	5			
	480.0861	0.6	1.2	17.5	C24 H17 N5 F2 S Cl	577.2	7.341	0.06	24	17	5		2	1	1
	480.0873	-0.6	-1.2	13.5	C21 H18 N5 O F3 S Cl	570.2	0.333	71.69	21	18	5	1	3	1	1
	480.0859	0.8	1.7	8.5	C20 H22 N O5 F3 S Cl	577.0	7.134	0.08	20	22	1	5	3	1	1
	480.0875	-0.8	-1.7	7.5	C17 H24 N5 O5 S Cl2	584.9	15.022	0.00	17	24	5	5		1	2
	480.0875	-0.8	-1.7	17.5	C23 H16 N5 O4 F Cl	577.6	7.780	0.04	23	16	5	4	1		1
	480.0858	0.9	1.9	25.5	C32 H15 N O F S	593.6	23.745	0.00	32	15	1	1	1	1	
	480.0857	1.0	2.1	13.5	C23 H19 N3 O F3 Cl2	584.4	14.577	0.00	23	19	3	1	3		2
	480.0880	-1.3	-2.7	12.5	C23 H22 N3 F2 S Cl2	584.7	14.906	0.00	23	22	3		2	1	2

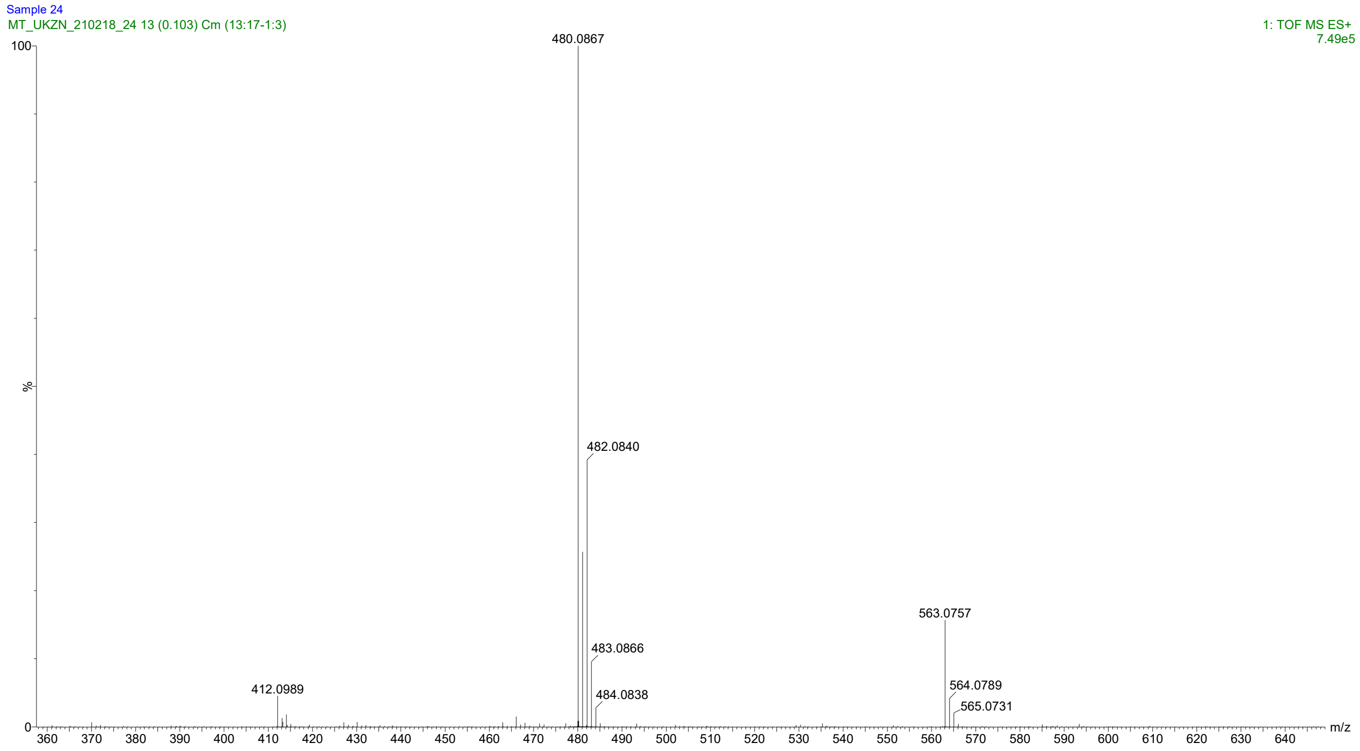

Figure S27: HRMS (ES+) for *1-(3-((7-chloroquinolin-4-yl)amino)propyl)-3-(6-(trifluoromethyl)benzo[d]thiazol-2-yl)urea* in DMSO-d6 (***6i***).

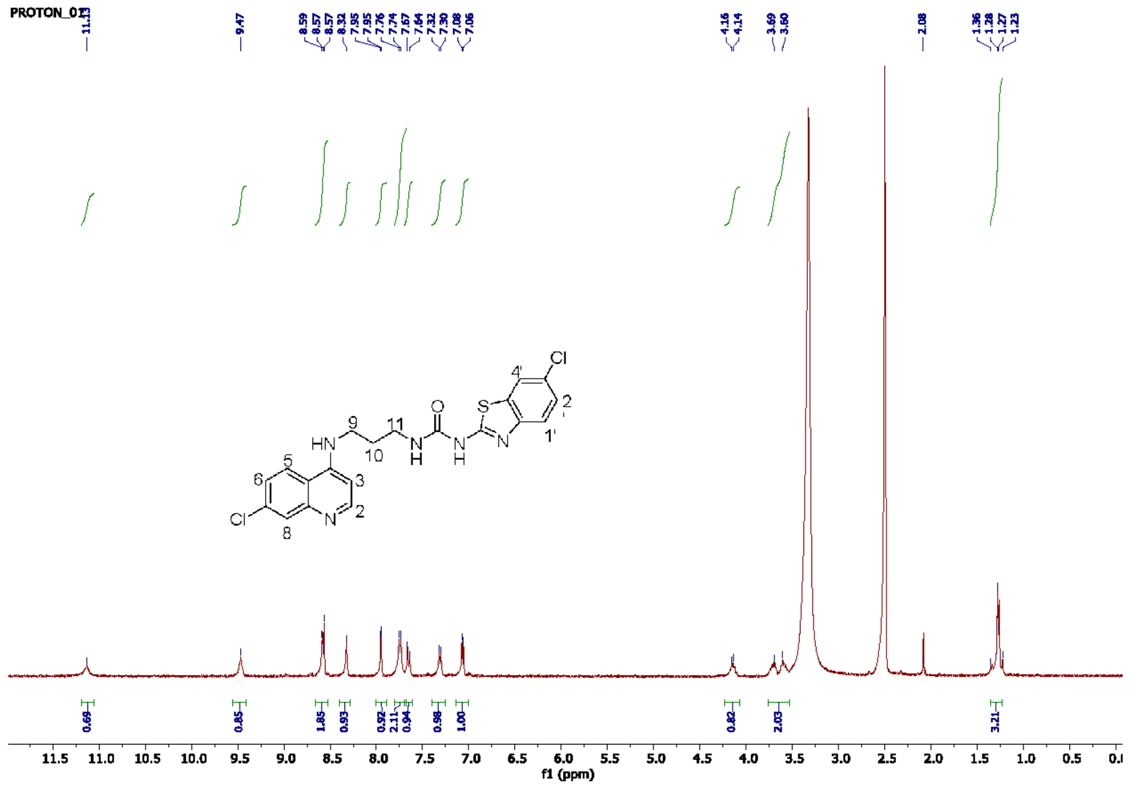

Figure S28: 400 MHz ^{1}H-NMR of *1-(6-chlorobenzo[d]thiazol-2-yl)-3-(3-((7-chloroquinolin-4-yl)amino)propyl)urea* in DMSO-d6 (***6j***).

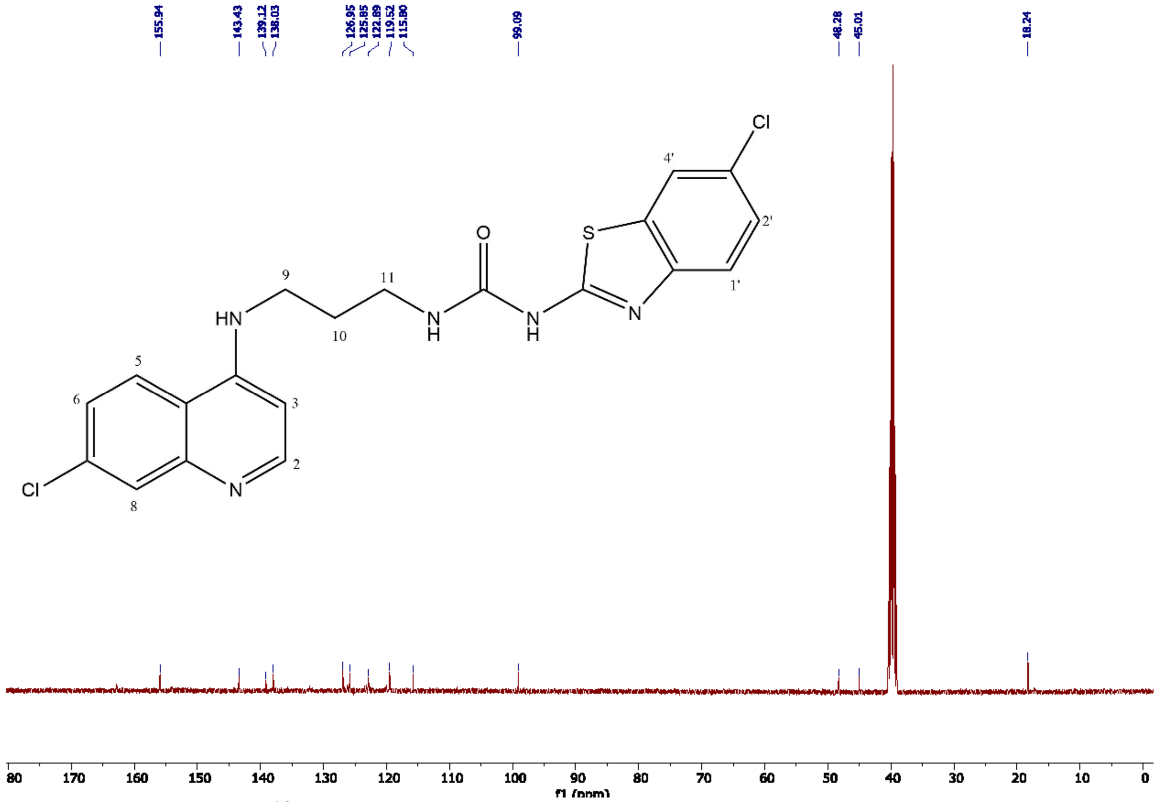

Figure S29: 101 MHz ^{13}C-NMR of *1-(6-chlorobenzo[d]thiazol-2-yl)-3-(3-((7-chloroquinolin-4-yl)amino)propyl)urea* in DMSO-d6 (***6j***).

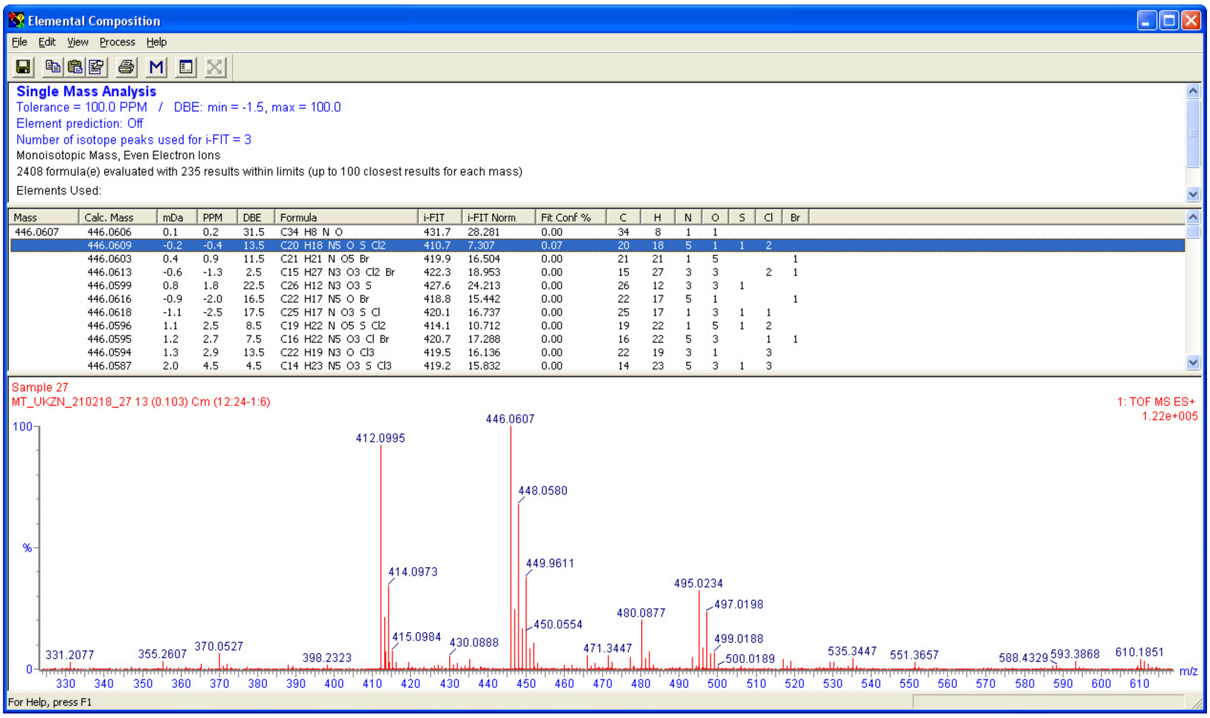

Mass	Calc. Mass	mDa	PPM	DBE	Formula	i-FIT	i-FIT Norm	Fit Conf %	C	H	N	O	S	Cl	Br
446.0607	446.0606	0.1	0.2	31.5	C34 H8 N O	431.7	28.281	0.00	34	8	1	1			
	446.0609	-0.2	-0.4	13.5	C20 H18 N5 O S Cl2	410.7	7.307	0.07	20	18	5	1	1	2	
	446.0603	0.4	0.9	11.5	C21 H21 N O5 Br	419.9	16.504	0.00	21	21	1	5			1
	446.0613	-0.6	-1.3	2.5	C15 H27 N3 O3 Cl2 Br	422.3	18.953	0.00	15	27	3	3		2	1
	446.0599	0.8	1.8	22.5	C26 H12 N3 O3 S	427.6	24.213	0.00	26	12	3	3	1		
	446.0616	-0.9	-2.0	16.5	C22 H17 N5 O Br	418.8	15.442	0.00	22	17	5	1			1
	446.0618	-1.1	-2.5	17.5	C25 H17 N O3 S Cl	420.1	16.737	0.00	25	17	1	3	1	1	
	446.0596	1.1	2.5	8.5	C19 H22 N O5 S Cl2	414.1	10.712	0.00	19	22	1	5	1	2	
	446.0595	1.2	2.7	7.5	C16 H22 N5 O3 Cl Br	420.7	17.288	0.00	16	22	5	3		1	1
	446.0594	1.3	2.9	13.5	C22 H19 N3 O Cl3	419.5	16.136	0.00	22	19	3	1		3	
	446.0587	2.0	4.5	4.5	C14 H23 N5 O3 S Cl3	419.2	15.832	0.00	14	23	5	3	1	3	

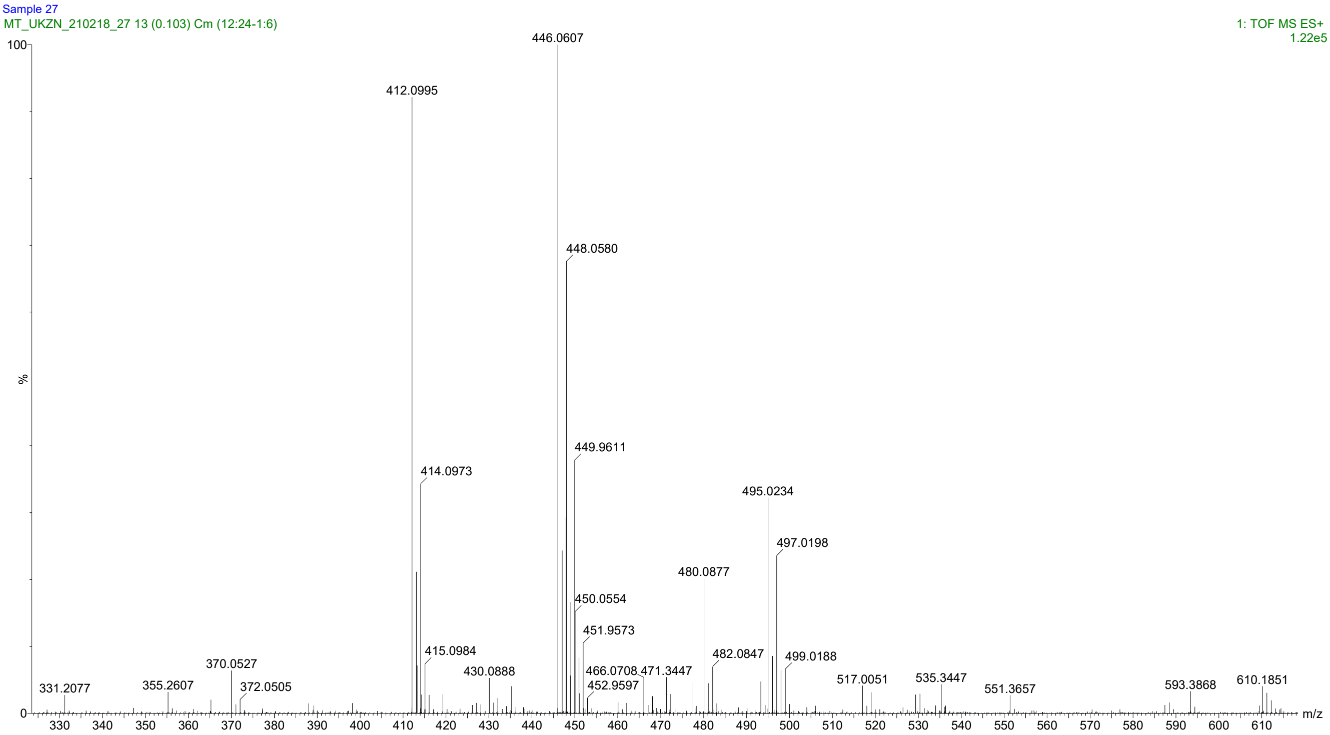

Figure S30: HRMS (ES+) for 1-(1-(7-chloroquinolin-4-ylamino)propan-2-yl)-3-(6-(trifluoromethyl)benzo[d]thiazol-2-yl)urea (**6j**).

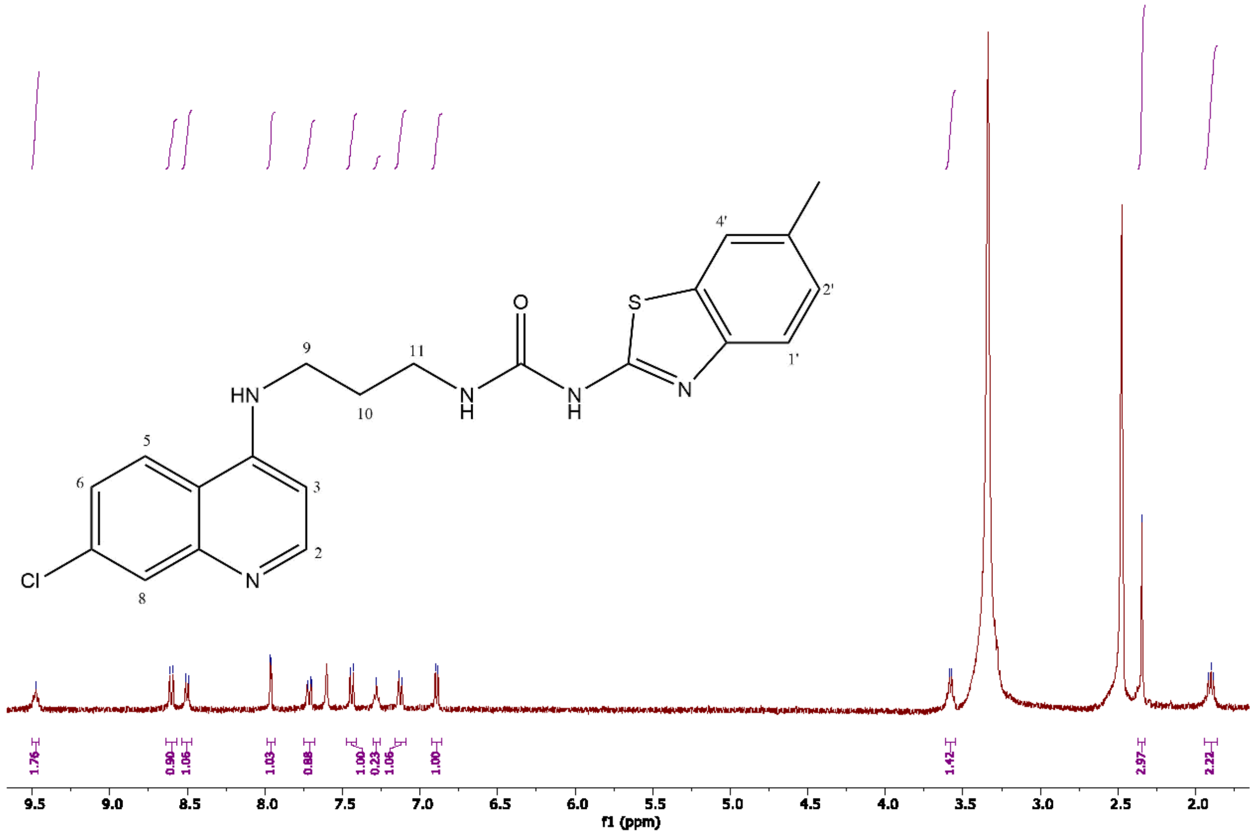

Figure S31: 400 MHz ^{1}H-NMR of *1-(3-((7-chloroquinolin-4-yl)amino)propyl)-3-(6-methylbenzo[d]thiazol-2-yl)urea* in DMSO-d6 *(**6k**).*

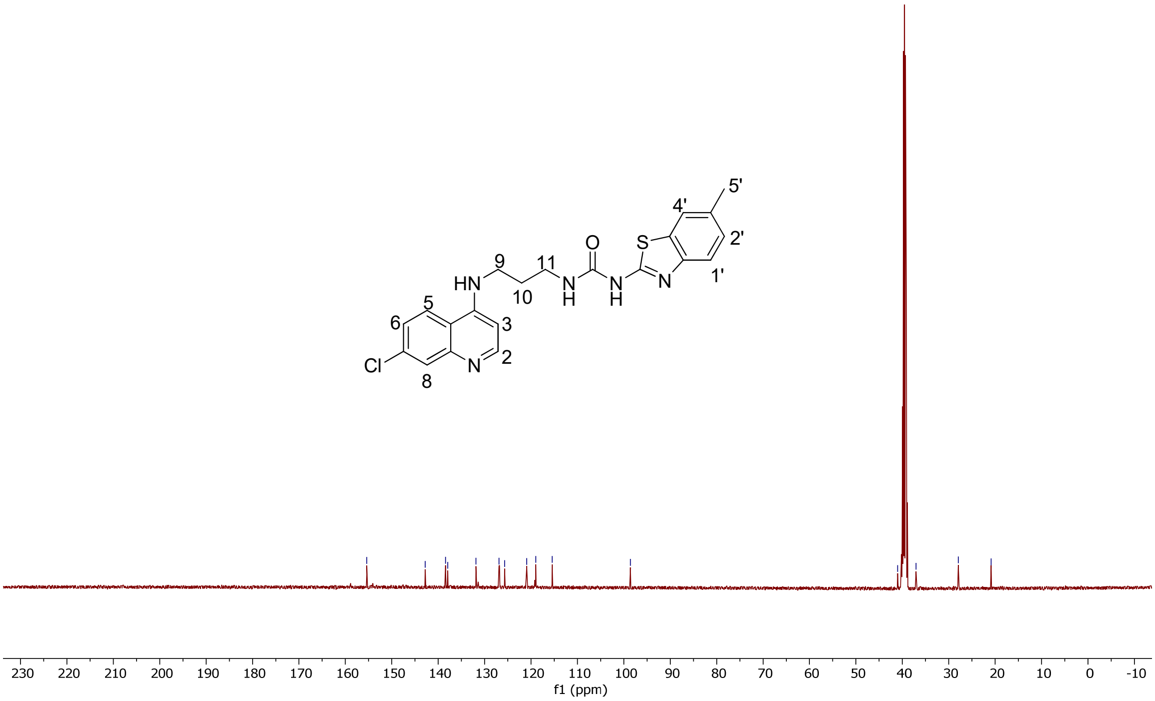

Figure S32: 101 MHz ^{13}C-NMR of *1-(3-((7-chloroquinolin-4-yl)amino)propyl)-3-(6-methylbenzo[d]thiazol-2-yl)urea* in DMSO-d6 *(**6k**).*

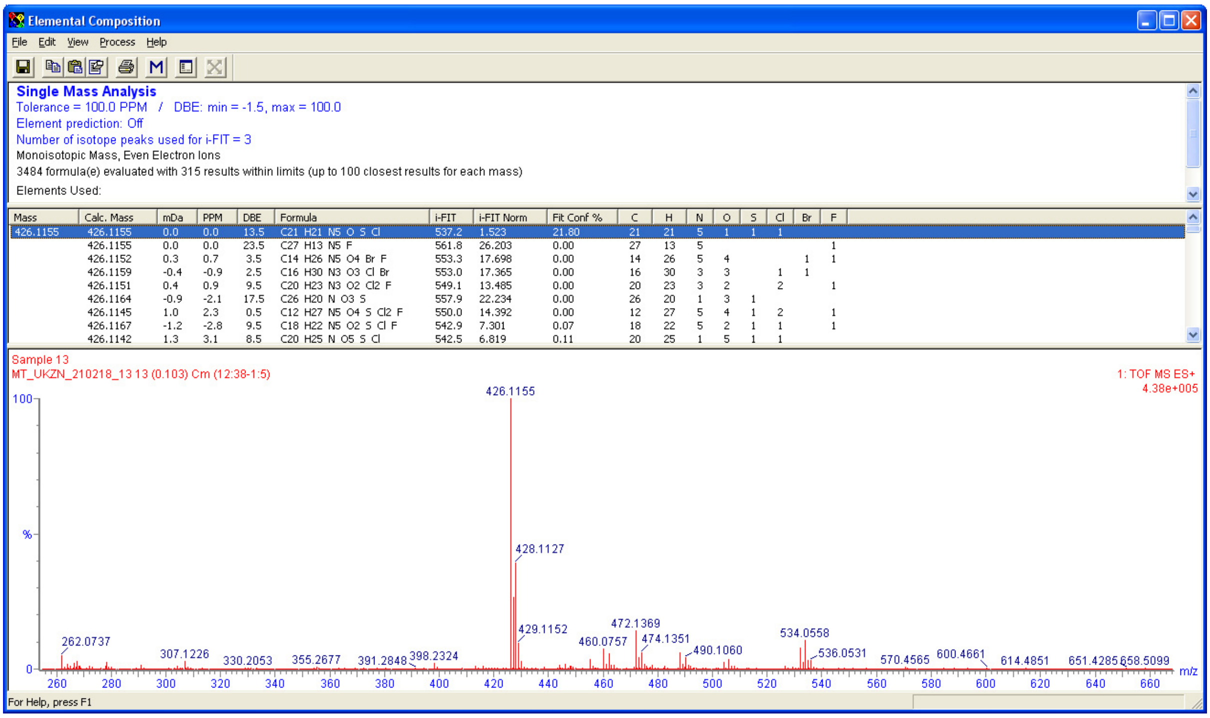

Single Mass Analysis
Tolerance = 100.0 PPM / DBE: min = -1.5, max = 100.0
Element prediction: Off
Number of isotope peaks used for i-FIT = 3
Monoisotopic Mass, Even Electron Ions
3484 formula(e) evaluated with 315 results within limits (up to 100 closest results for each mass)
Elements Used:

Mass	Calc. Mass	mDa	PPM	DBE	Formula	i-FIT	i-FIT Norm	Fit Conf %	C	H	N	O	S	Cl	Br	F
426.1155	426.1155	0.0	0.0	13.5	C21 H21 N5 O S Cl	537.2	1.523	21.80	21	21	5	1	1	1		
	426.1155	0.0	0.0	23.5	C27 H13 N5 F	561.8	26.203	0.00	27	13	5					1
	426.1152	0.3	0.7	3.5	C14 H26 N5 O4 Br F	553.3	17.698	0.00	14	26	5	4			1	1
	426.1159	-0.4	-0.9	2.5	C16 H30 N3 O3 Cl Br	553.0	17.365	0.00	16	30	3	3		1	1	
	426.1151	0.4	0.9	9.5	C20 H23 N3 O2 Cl2 F	549.1	13.485	0.00	20	23	3	2		2		1
	426.1164	-0.9	-2.1	17.5	C26 H20 N O3 S	557.9	22.234	0.00	26	20	1	3	1			
	426.1145	1.0	2.3	0.5	C12 H27 N5 O4 S Cl2 F	550.0	14.392	0.00	12	27	5	4	1	2		1
	426.1167	-1.2	-2.8	9.5	C18 H22 N5 O2 S Cl F	542.9	7.301	0.07	18	22	5	2	1	1		1
	426.1142	1.3	3.1	8.5	C20 H25 N O5 S Cl	542.5	6.819	0.11	20	25	1	5	1	1		

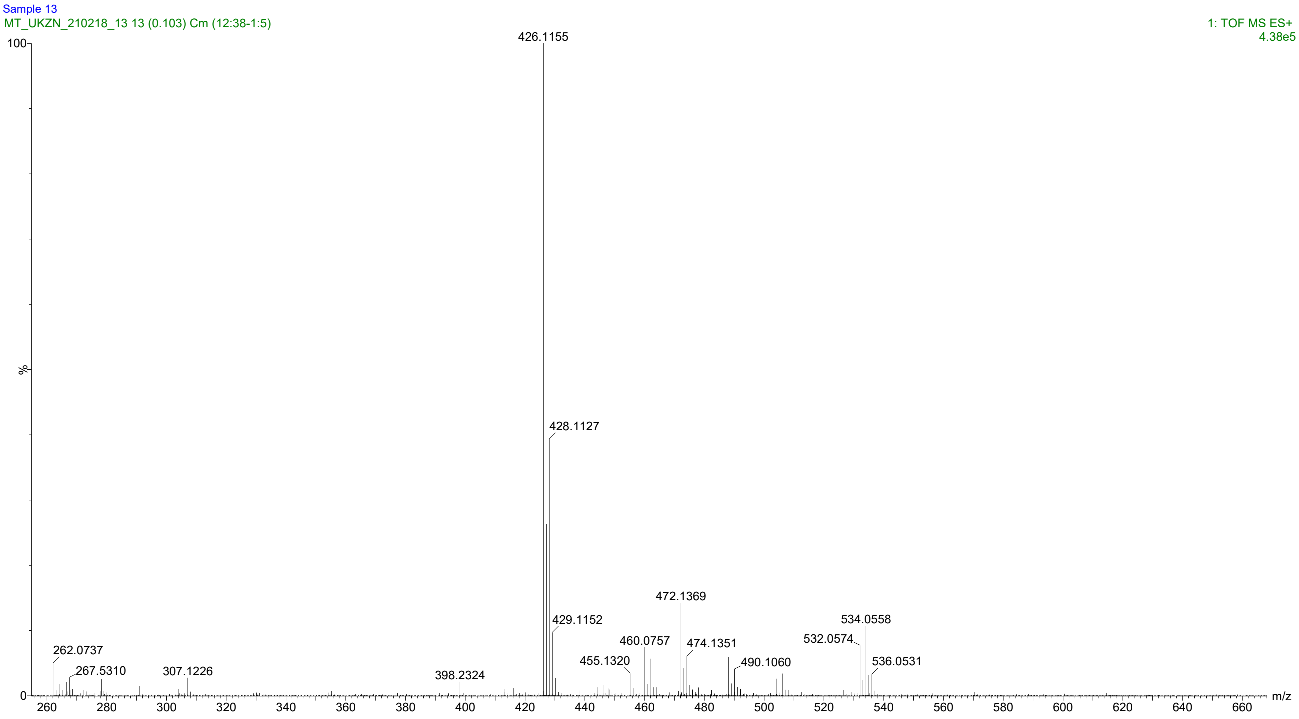

Figure S33: HRMS (ES$^+$) for *1-(3-((7-chloroquinolin-4-yl)amino)propyl)-3-(6-methylbenzo[d]thiazol-2-yl)urea* (**6k**).

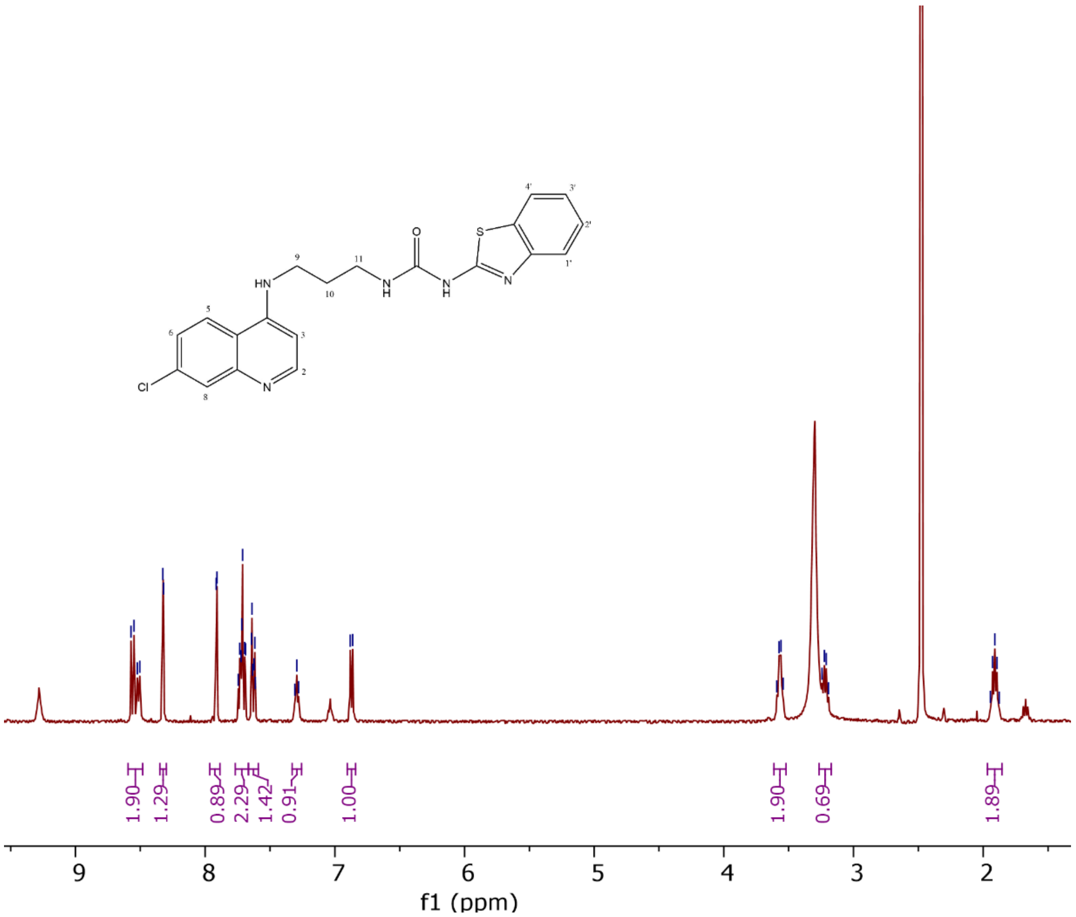

Figure S34: 400 MHz ^{1}H-NMR of *1-(3-((7-chloroquinolin-4-yl)amino)propyl)-3-(benzo[d]thiazol-2-yl)urea* in DMSO-d6 (***6l***).

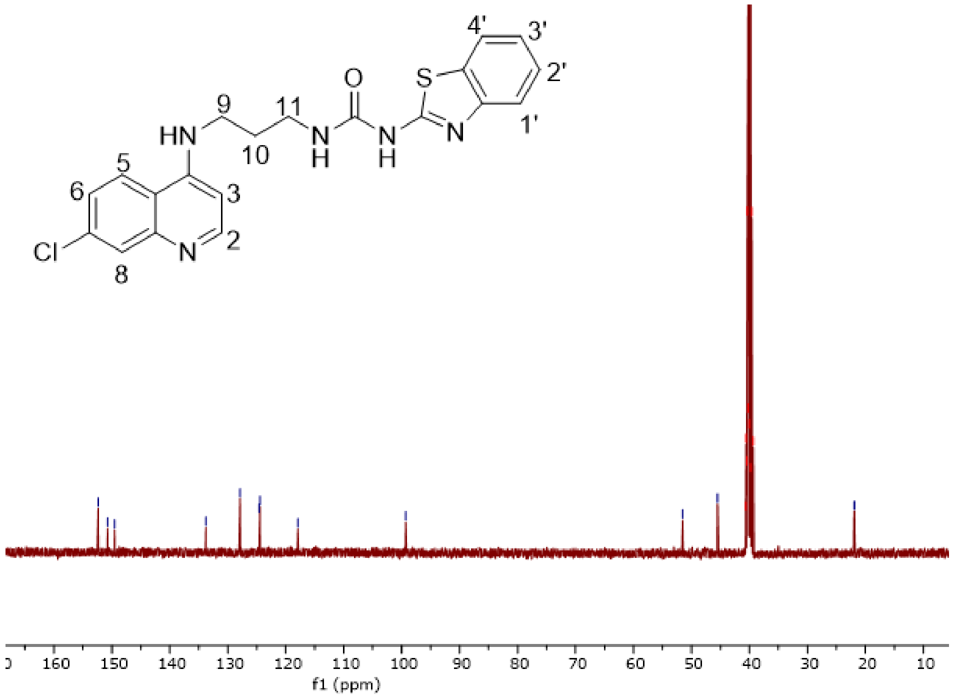

Figure S35: 101 MHz ^{13}C-NMR of *1-(3-((7-chloroquinolin-4-yl)amino)propyl)-3-(benzo[d]thiazol-2-yl)urea* in DMSO-d6 (***6l***).

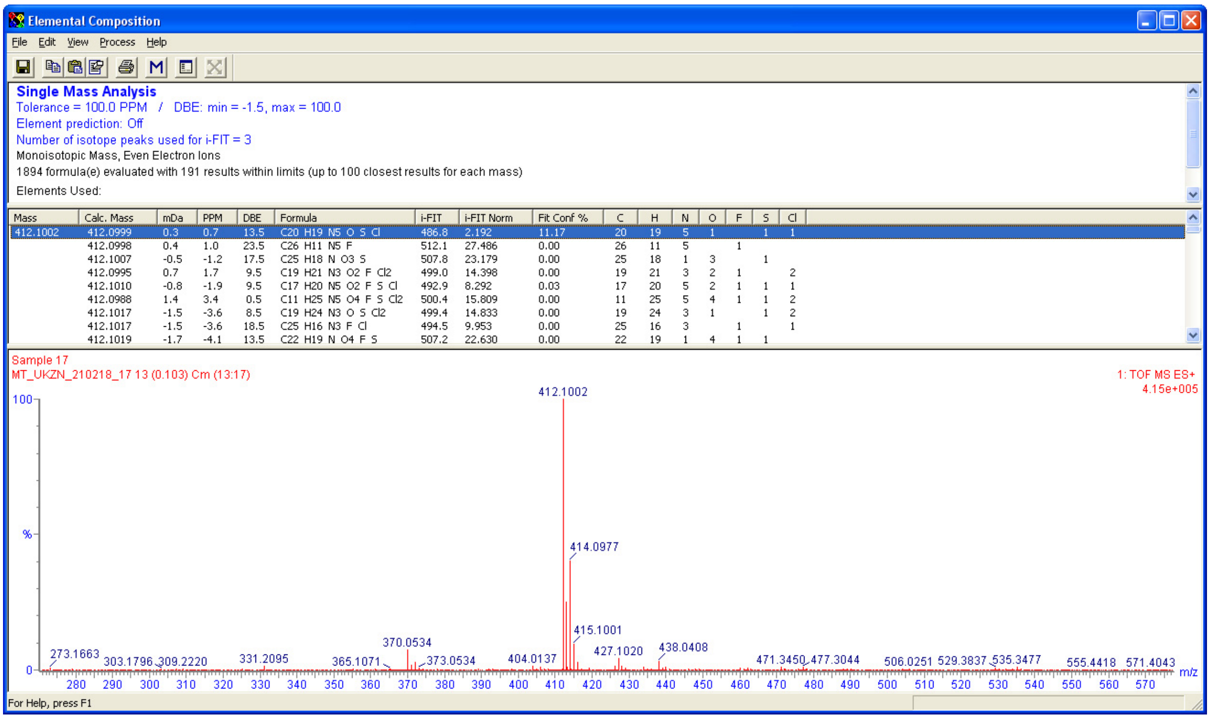

Mass	Calc. Mass	mDa	PPM	DBE	Formula	i-FIT	i-FIT Norm	Fit Conf %	C	H	N	O	F	S	Cl
412.1002	412.0999	0.3	0.7	13.5	C20 H19 N5 O S Cl	486.8	2.192	11.17	20	19	5	1		1	1
	412.0998	0.4	1.0	23.5	C26 H11 N5 F	512.1	27.486	0.00	26	11	5		1		
	412.1007	-0.5	-1.2	17.5	C25 H18 N O3 S	507.8	23.179	0.00	25	18	1	3		1	
	412.0995	0.7	1.7	9.5	C19 H21 N3 O2 F Cl2	499.0	14.398	0.00	19	21	3	2	1		2
	412.1010	-0.8	-1.9	9.5	C17 H20 N5 O2 F S Cl	492.9	8.292	0.03	17	20	5	2	1	1	1
	412.0988	1.4	3.4	0.5	C11 H25 N5 O4 F S Cl2	500.4	15.809	0.00	11	25	5	4	1	1	2
	412.1017	-1.5	-3.6	8.5	C19 H24 N3 O S Cl2	499.4	14.833	0.00	19	24	3	1		1	2
	412.1017	-1.5	-3.6	18.5	C25 H16 N3 F Cl	494.5	9.953	0.00	25	16	3		1		1
	412.1019	-1.7	-4.1	13.5	C22 H19 N O4 F S	507.2	22.630	0.00	22	19	1	4	1	1	

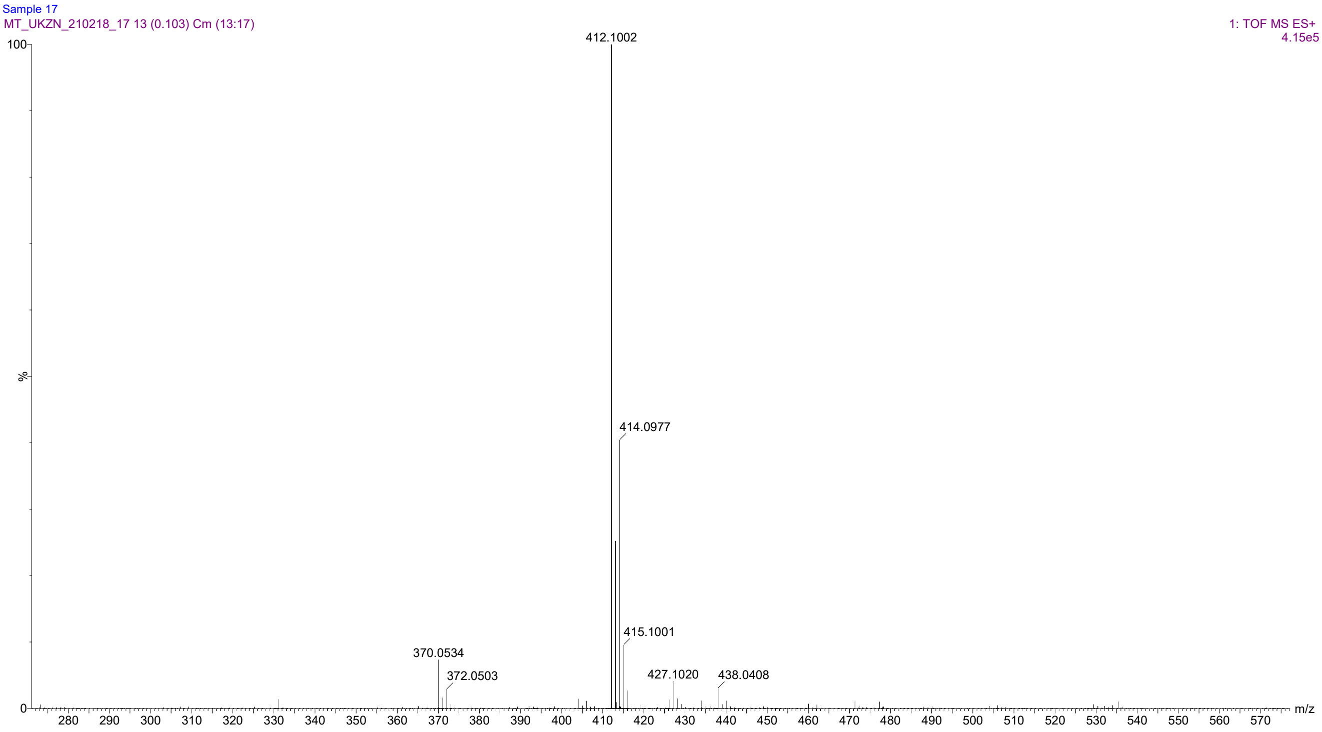

Figure S36: HRMS (ES$^+$) for *1-(3-((7-chloroquinolin-4-yl)amino)propyl)-3-(benzo[d]thiazol-2-yl)urea* (***6l***).

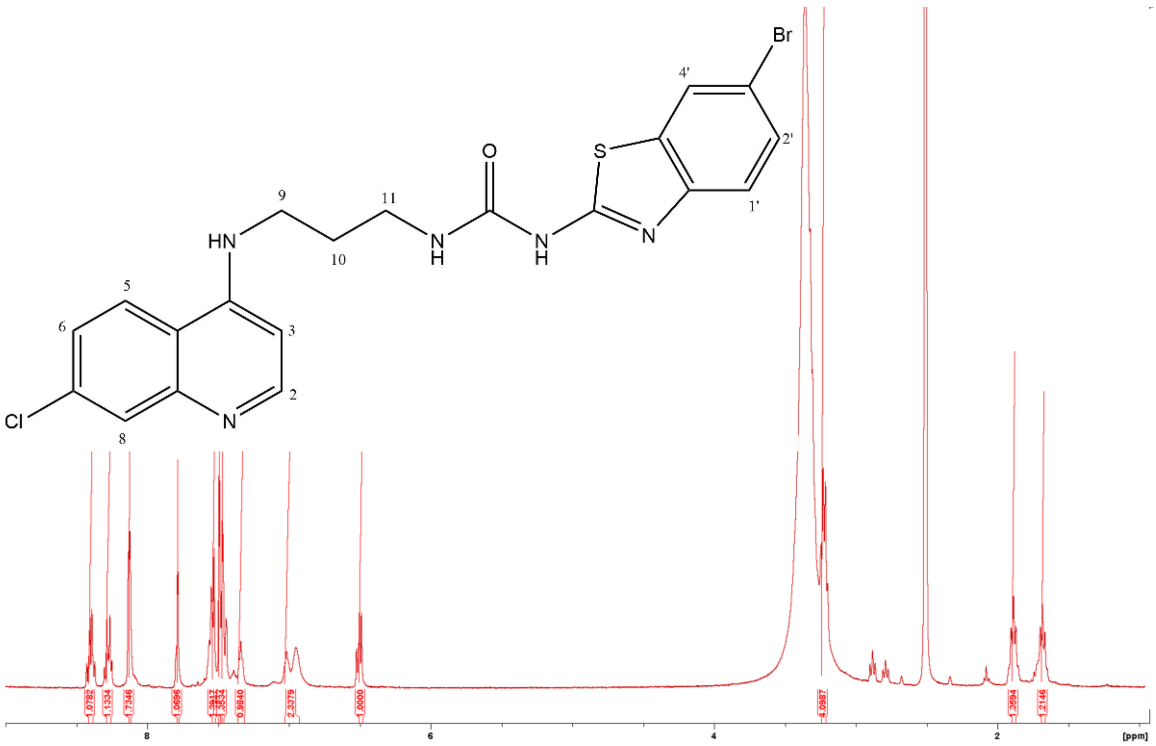

Figure S37: 400 MHz ^{1}H-NMR of *1-(6-bromobenzo[d]thiazol-2-yl)-3-(3-((7-chloroquinolin-4-yl)amino)propyl)urea* in DMSO-d6 ***(6m)***.

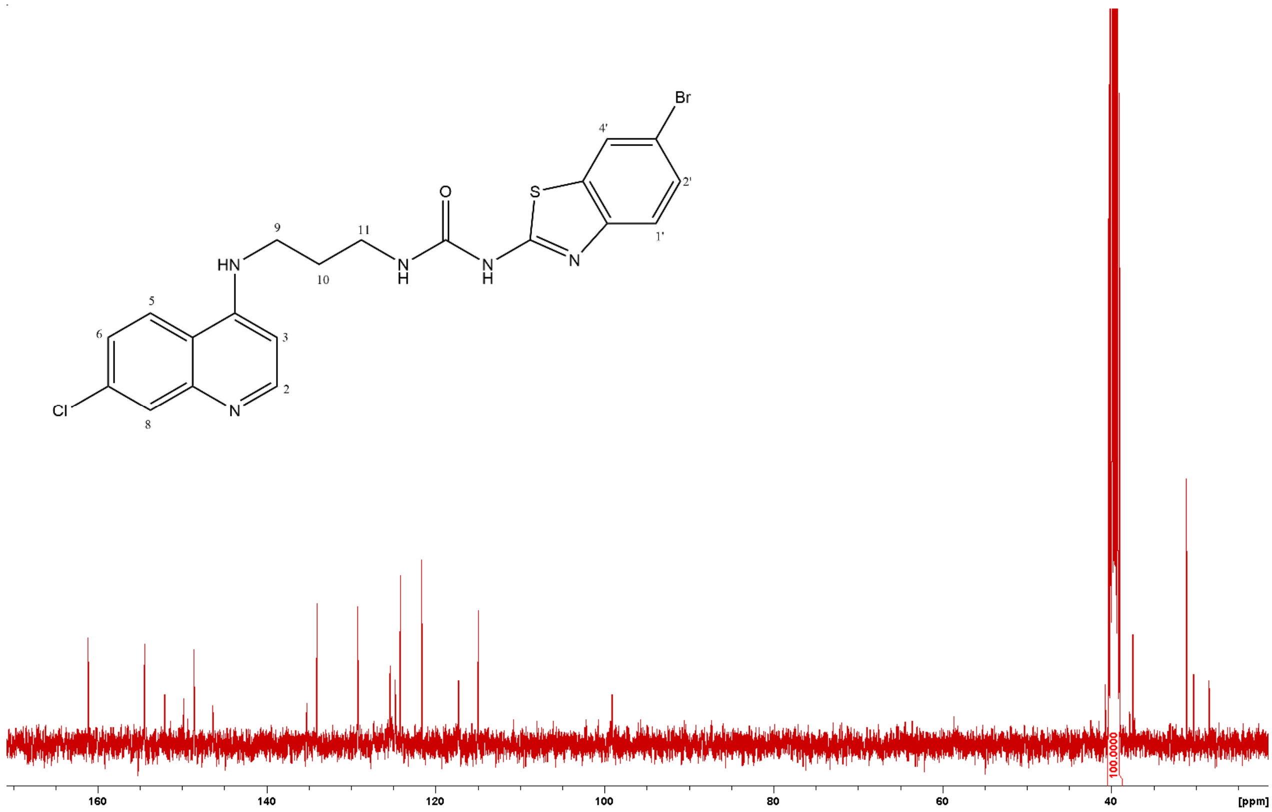

Figure S38: 101 MHz ^{13}C-NMR of *1-(6-bromobenzo[d]thiazol-2-yl)-3-(3-((7-chloroquinolin-4-yl)amino)propyl)urea* in DMSO-d6 ***(6m)***.

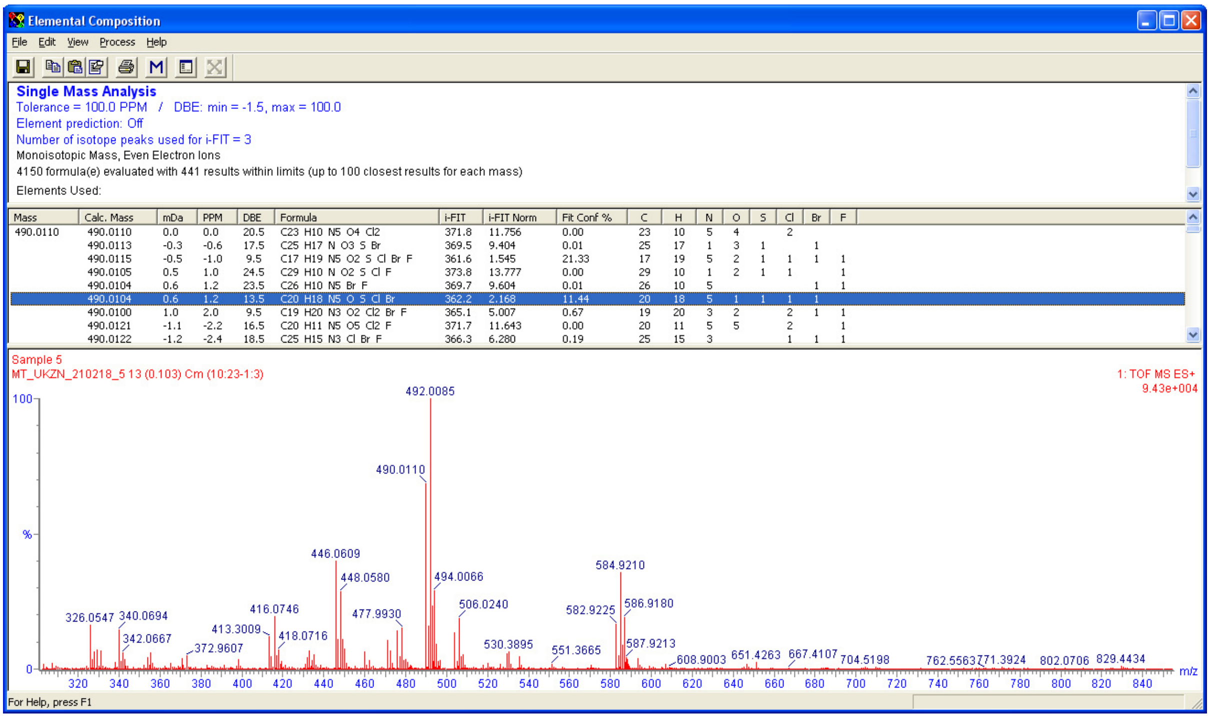

Mass	Calc. Mass	mDa	PPM	DBE	Formula	i-FIT	i-FIT Norm	Fit Conf %	C	H	N	O	S	Cl	Br	F
490.0110	490.0110	0.0	0.0	20.5	C23 H10 N5 O4 Cl2	371.8	11.756	0.00	23	10	5	4		2		
	490.0113	-0.3	-0.6	17.5	C25 H17 N O3 S Br	369.5	9.404	0.01	25	17	1	3	1		1	
	490.0115	-0.5	-1.0	9.5	C17 H19 N5 O2 S Cl Br F	361.6	1.545	21.33	17	19	5	2	1	1	1	1
	490.0105	0.5	1.0	24.5	C29 H10 N O2 S Cl F	373.8	13.777	0.00	29	10	1	2	1	1		1
	490.0104	0.6	1.2	23.5	C26 H10 N5 Br F	369.7	9.604	0.01	26	10	5				1	1
	490.0104	0.6	1.2	13.5	C20 H18 N5 O S Cl Br	362.2	2.168	11.44	20	18	5	1	1	1	1	
	490.0100	1.0	2.0	9.5	C19 H20 N3 O2 Cl2 Br F	365.1	5.007	0.67	19	20	3	2		2	1	1
	490.0121	-1.1	-2.2	16.5	C20 H11 N5 O5 Cl2 F	371.7	11.643	0.00	20	11	5	5		2		1
	490.0122	-1.2	-2.4	18.5	C25 H15 N3 Cl Br F	366.3	6.280	0.19	25	15	3			1	1	1

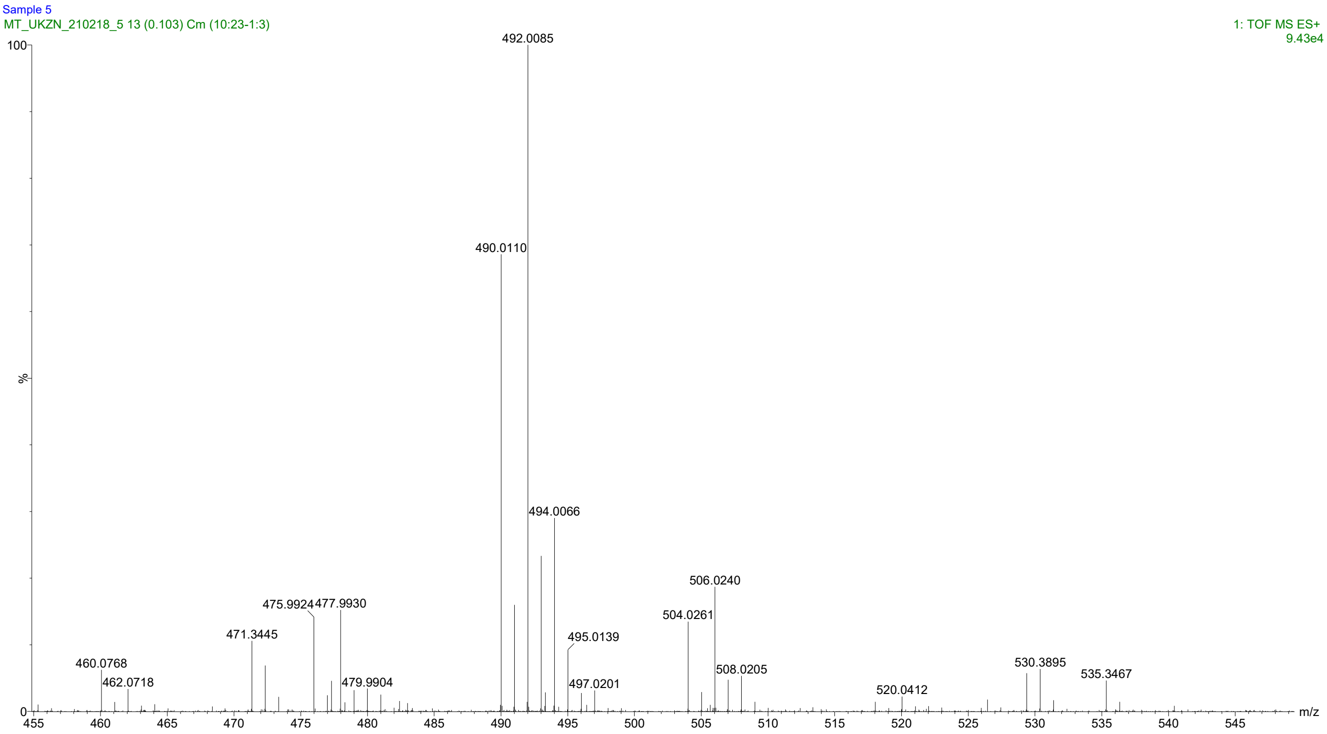

Figure S39: HRMS (ES$^+$) for *1-(6-bromobenzo[d]thiazol-2-yl)-3-(3-((7-chloroquinolin-4-yl)amino)propyl)urea* (**6m**).

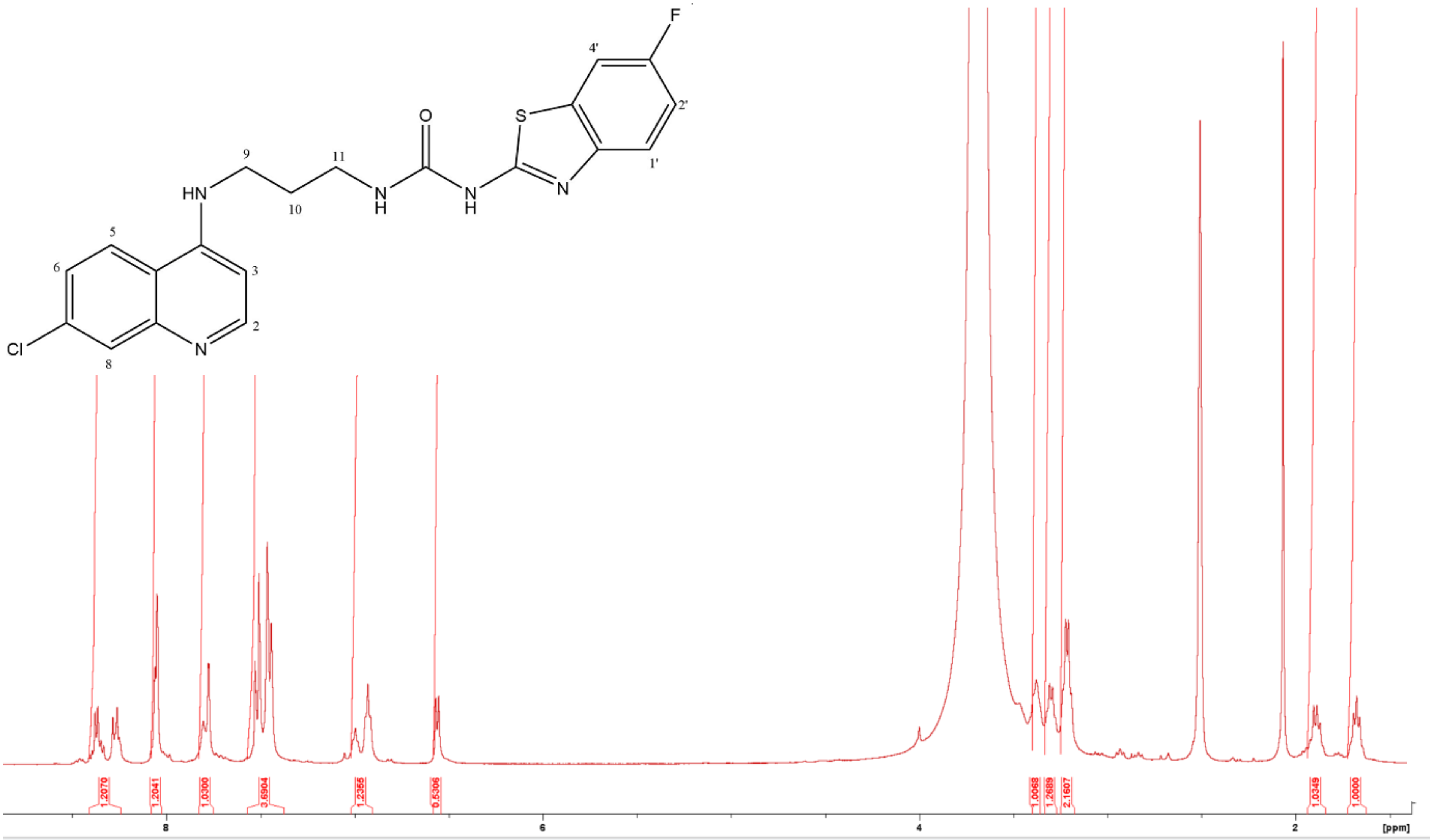

Figure S40: 400 MHz ^{1}H-NMR of *1-(6-flourobenzo[d]thiazol-2-yl)-3-(3-((7-chloroquinolin-4-yl)amino)propyl)urea in DMSO-d6 (**6n**).*

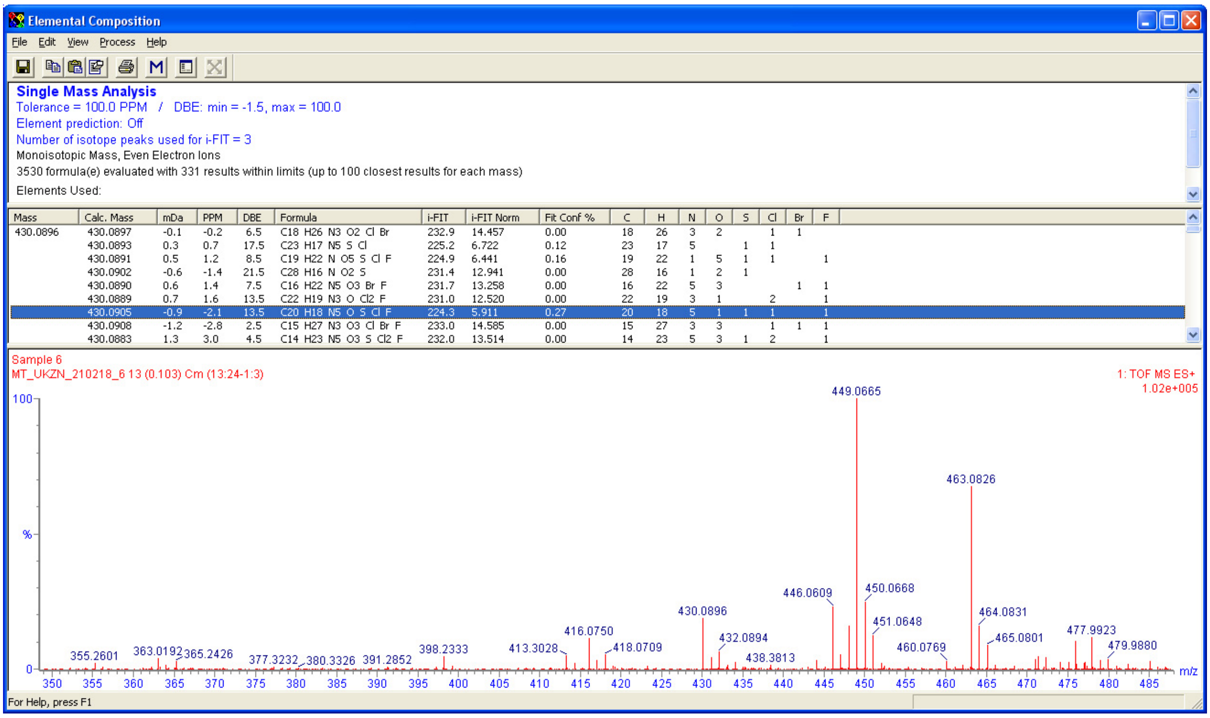

Mass	Calc. Mass	mDa	PPM	DBE	Formula	i-FIT	i-FIT Norm	Fit Conf %	C	H	N	O	S	Cl	Br	F
430.0896	430.0897	-0.1	-0.2	6.5	C18 H26 N3 O2 Cl Br	232.9	14.457	0.00	18	26	3	2		1	1	
	430.0893	0.3	0.7	17.5	C23 H17 N5 S Cl	225.2	6.722	0.12	23	17	5		1	1		
	430.0891	0.5	1.2	8.5	C19 H22 N O5 S Cl F	224.9	6.441	0.16	19	22	1	5	1	1		1
	430.0902	-0.6	-1.4	21.5	C28 H16 N O2 S	231.4	12.941	0.00	28	16	1	2	1			
	430.0890	0.6	1.4	7.5	C16 H22 N5 O3 Br F	231.7	13.258	0.00	16	22	5	3			1	1
	430.0889	0.7	1.6	13.5	C22 H19 N3 O Cl2 F	231.0	12.520	0.00	22	19	3	1		2		1
	430.0905	-0.9	-2.1	13.5	C20 H18 N5 O S Cl F	224.3	5.911	0.27	20	18	5	1	1	1		1
	430.0908	-1.2	-2.8	2.5	C15 H27 N3 O3 Cl Br F	233.0	14.585	0.00	15	27	3	3		1	1	1
	430.0883	1.3	3.0	4.5	C14 H23 N5 O3 S Cl2 F	232.0	13.514	0.00	14	23	5	3	1	2		1

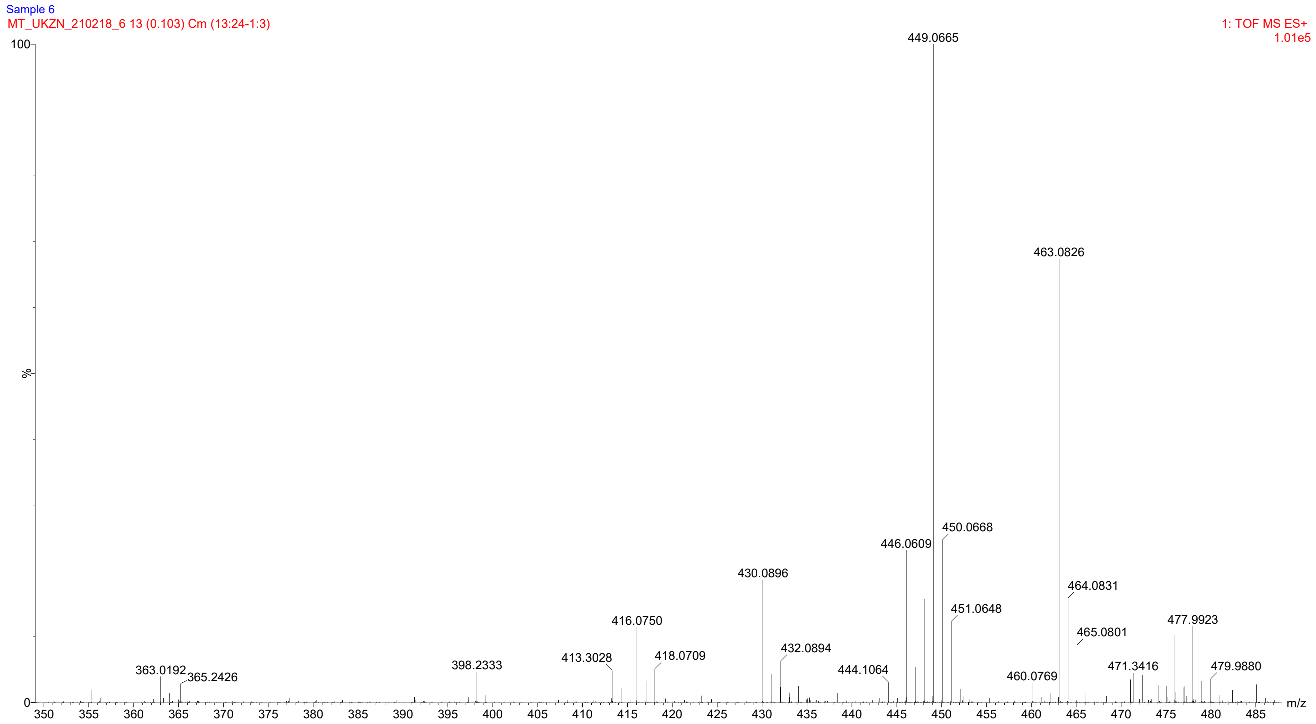

Figure S41: HRMS (ES+) for *1-(6-fluorobenzo[d]thiazol-2-yl)-3-(3-((7-chloroquinolin-4-yl)amino)propyl)urea* (***6n***).

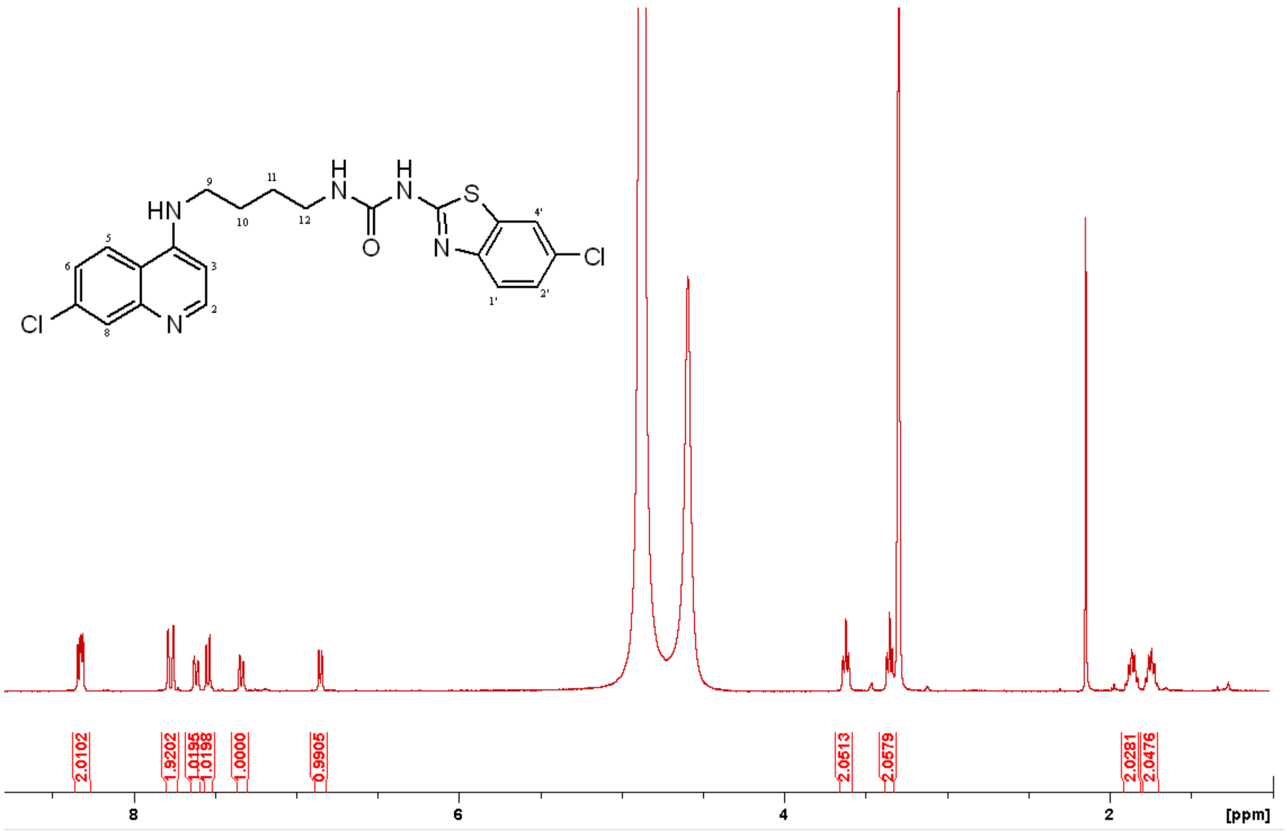

Figure S42: 400 MHz ^{1}H NMR for *1-(6-chlorobenzo[d]thiazol-2-yl)-3-(4-((7-chloroquinolin-4-yl)amino)butyl)urea* in MeOH-d4 (***6o***).

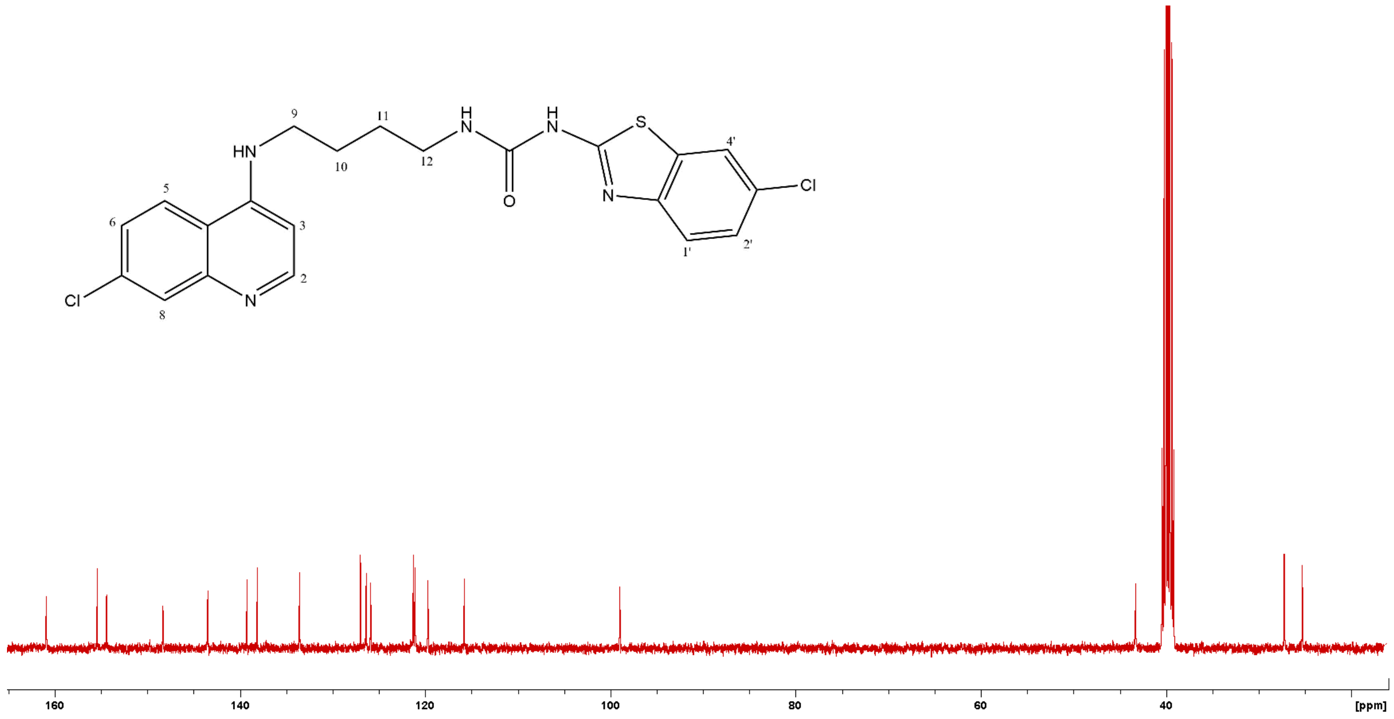

Figure S43: 101 MHz ^{13}C NMR for *1-(6-chlorobenzo[d]thiazol-2-yl)-3-(4-((7-chloroquinolin-4-yl)amino)butyl)urea* in DMSO-d6 (***6o***).

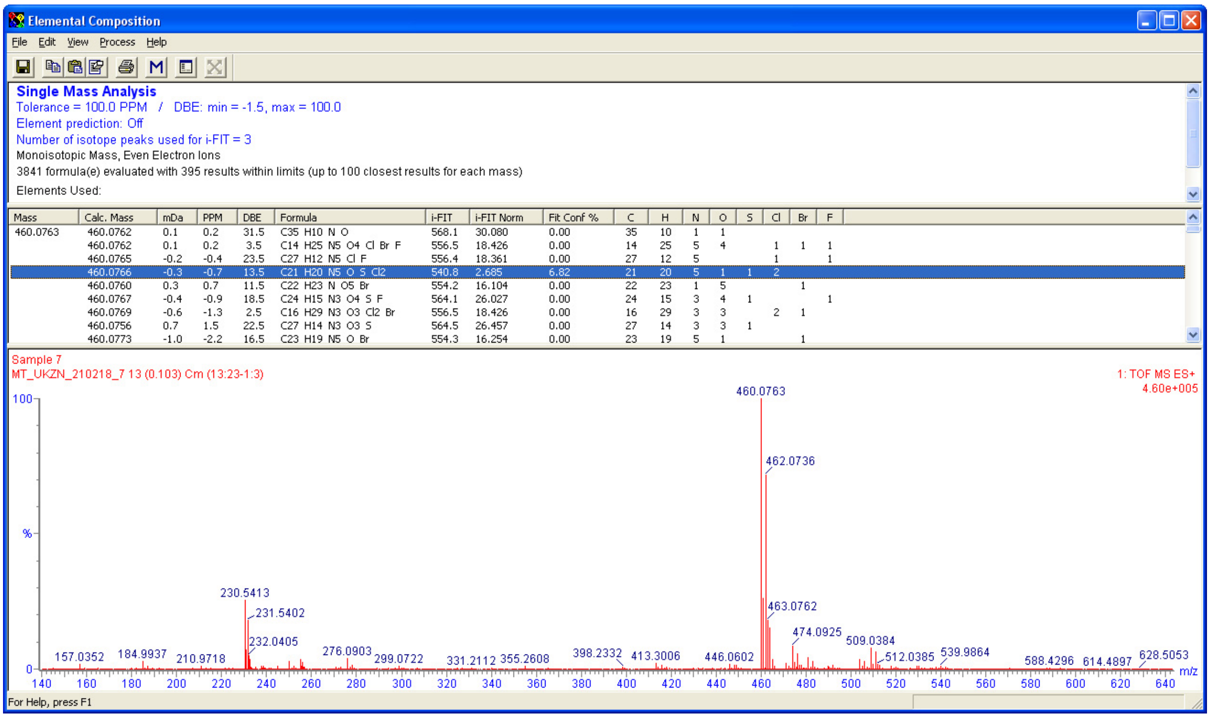

Elemental Composition

File Edit View Process Help

Single Mass Analysis

Tolerance = 100.0 PPM / DBE: min = -1.5, max = 100.0

Element prediction: Off

Number of isotope peaks used for i-FIT = 3

Monoisotopic Mass, Even Electron Ions

3841 formula(e) evaluated with 395 results within limits (up to 100 closest results for each mass)

Elements Used:

Mass	Calc. Mass	mDa	PPM	DBE	Formula	i-FIT	i-FIT Norm	Fit Conf %	C	H	N	O	S	Cl	Br	F
460.0763	460.0762	0.1	0.2	31.5	C35 H10 N O	568.1	30.080	0.00	35	10	1	1				
	460.0762	0.1	0.2	3.5	C14 H25 N5 O4 Cl Br F	556.5	18.426	0.00	14	25	5	4		1	1	1
	460.0765	-0.2	-0.4	23.5	C27 H12 N5 Cl F	556.4	18.361	0.00	27	12	5			1		1
	460.0766	-0.3	-0.7	13.5	C21 H20 N5 O S Cl2	540.8	2.685	6.82	21	20	5	1	1	2		
	460.0760	0.3	0.7	11.5	C22 H23 N O5 Br	554.2	16.104	0.00	22	23	1	5			1	
	460.0767	-0.4	-0.9	18.5	C24 H15 N3 O4 S F	564.1	26.027	0.00	24	15	3	4	1			1
	460.0769	-0.6	-1.3	2.5	C16 H29 N3 O3 Cl2 Br	556.5	18.426	0.00	16	29	3	3		2	1	
	460.0756	0.7	1.5	22.5	C27 H14 N3 O3 S	564.5	26.457	0.00	27	14	3	3	1			
	460.0773	-1.0	-2.2	16.5	C23 H19 N5 O Br	554.3	16.254	0.00	23	19	5	1			1	

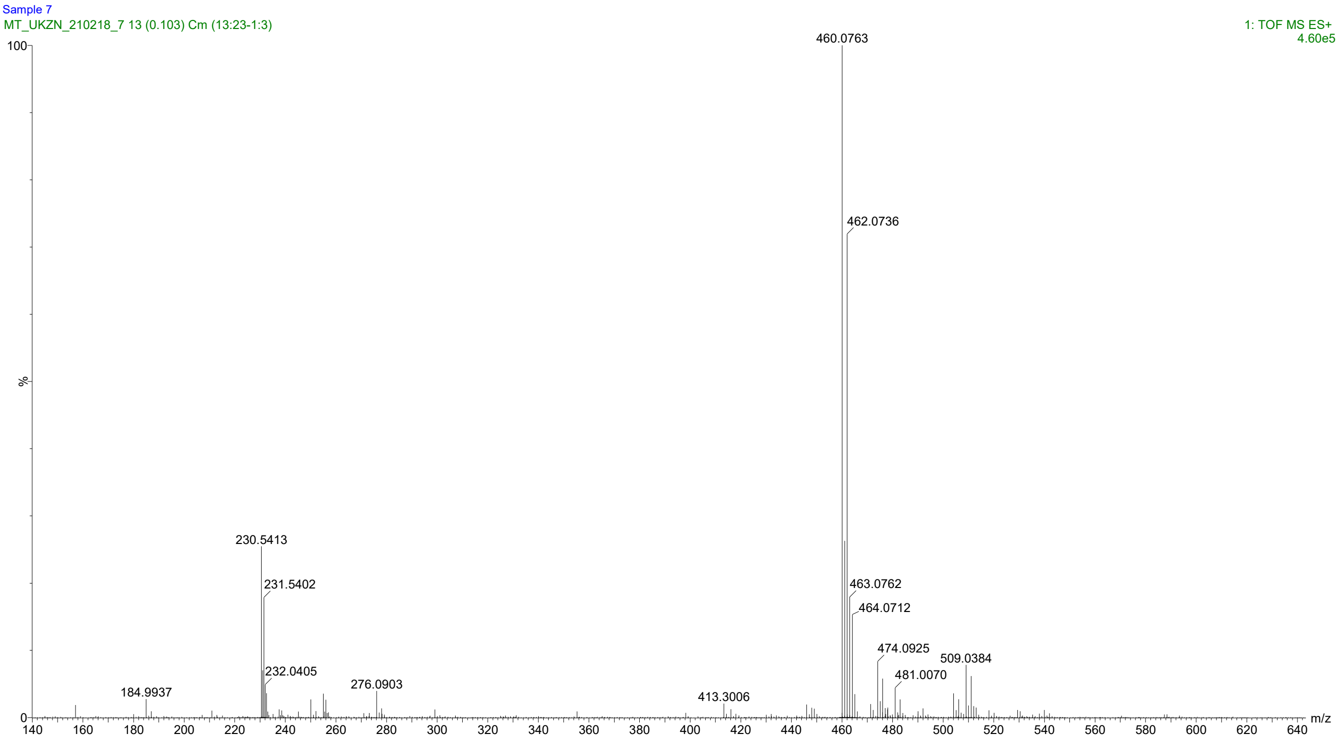

Figure S44: HRMS (ES+) for *1-(6-chlorobenzo[d]thiazol-2-yl)-3-(4-((7-chloroquinolin-4-yl)amino)butyl)urea* (***6o***).

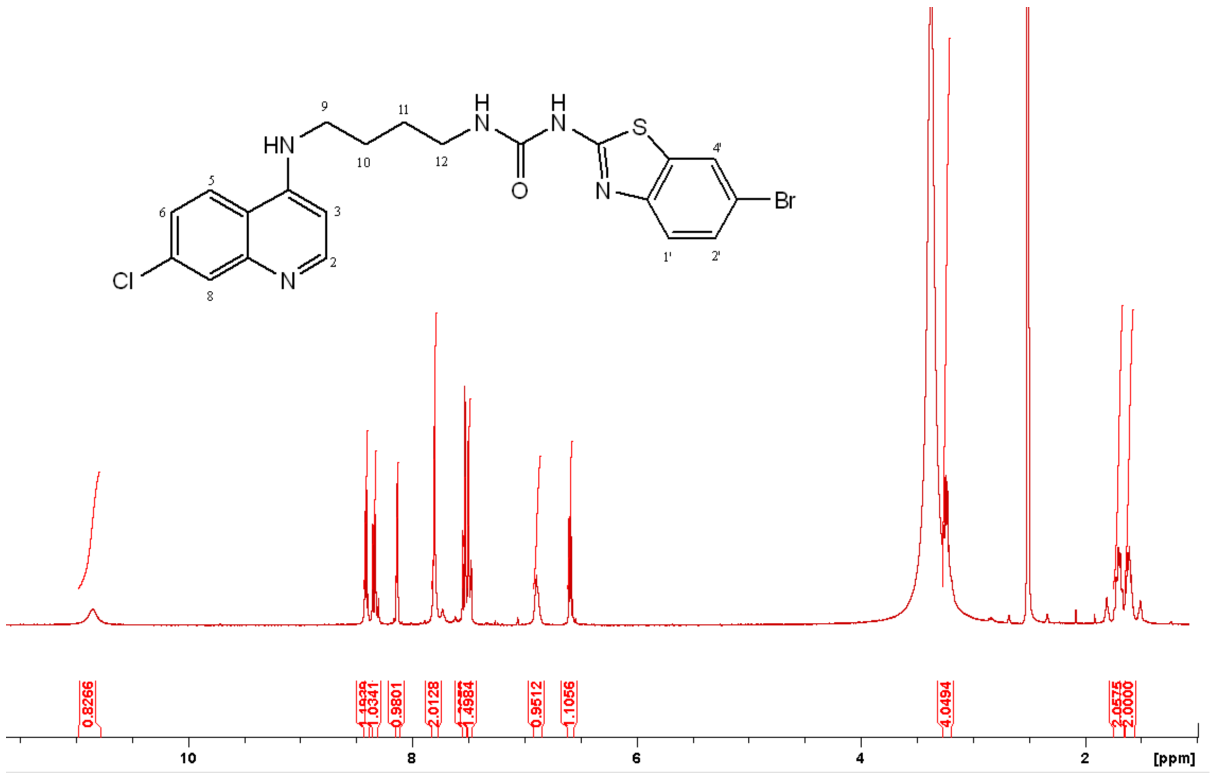

Figure S45: 400 MHz ^{1}H NMR for *1-(6-bromobenzo[d]thiazol-2-yl)-3-(4-((7-chloroquinolin-4-yl)amino)butyl)urea)* in DMSO-d6 (***6p***).

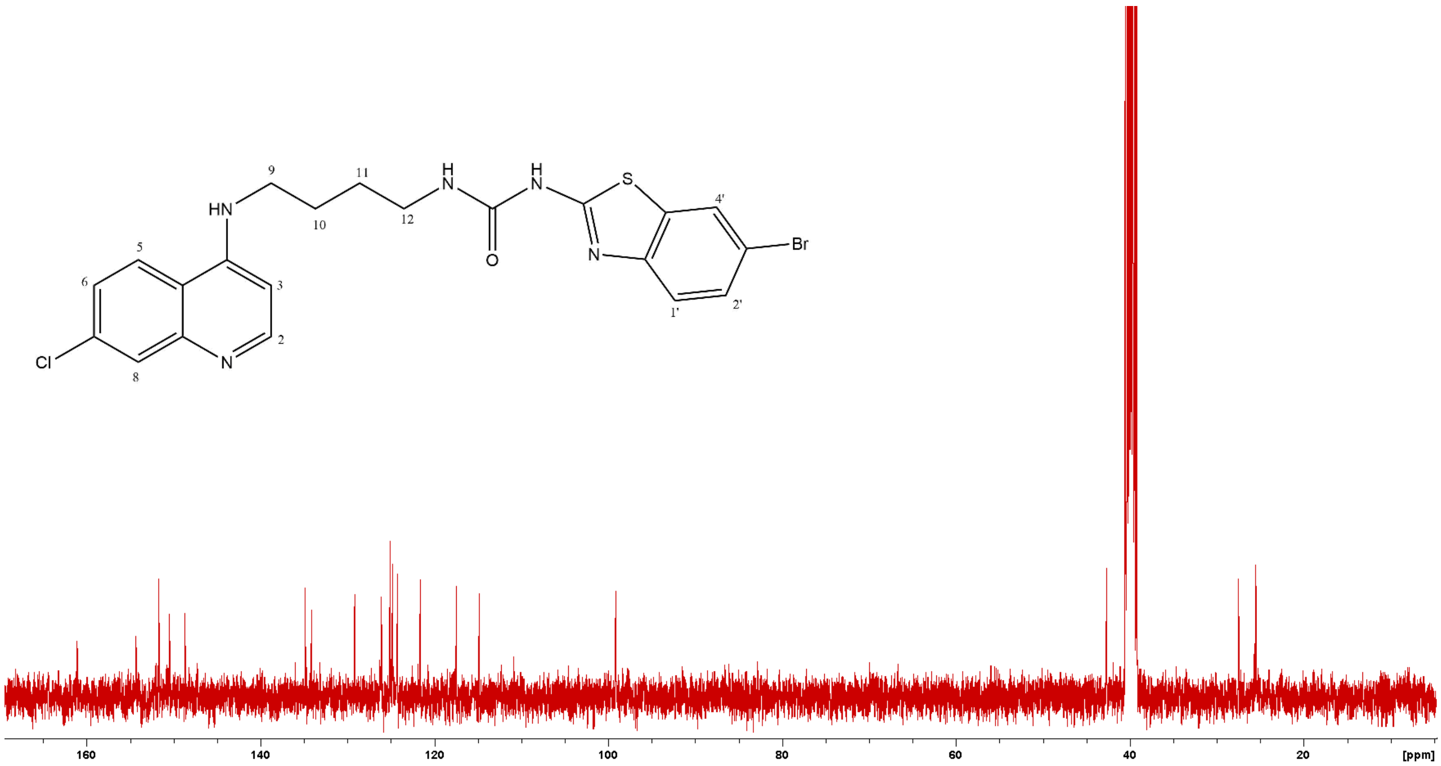

Figure S46: 101 MHz ^{13}C NMR for *1-(6-bromobenzo[d]thiazol-2-yl)-3-(4-((7-chloroquinolin-4-yl)amino)butyl)urea)* in DMSO-d6 (***6p***).

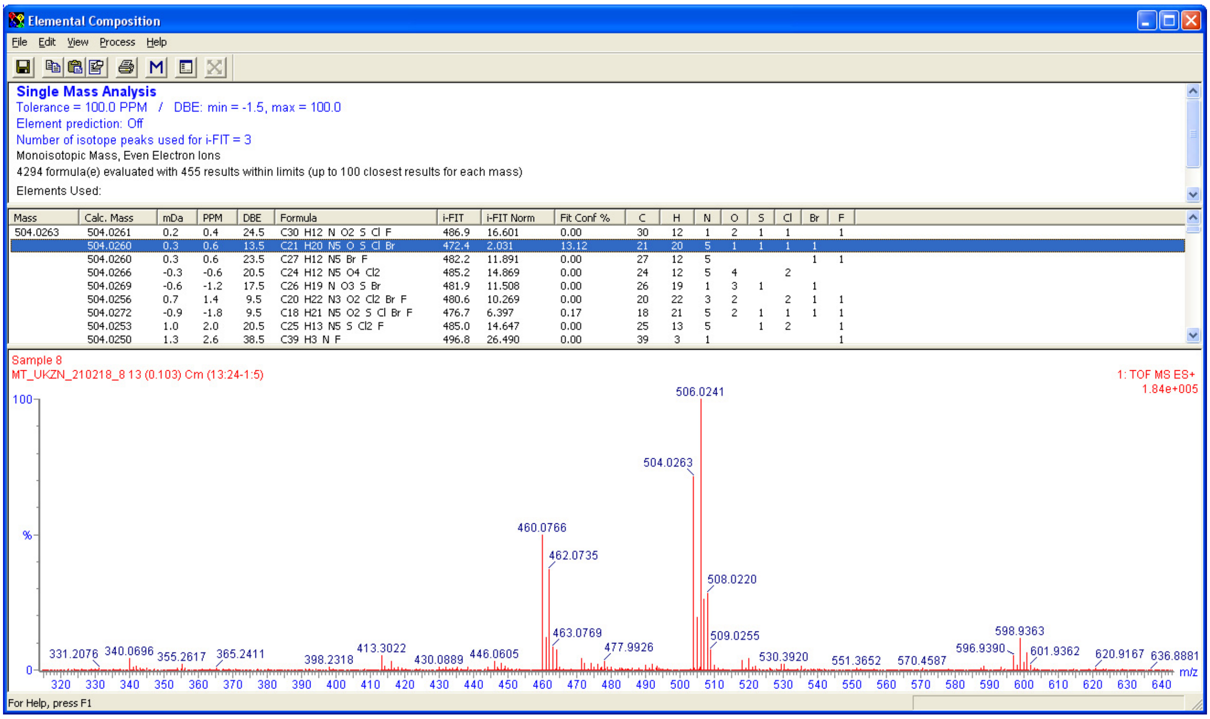
Elemental Composition

File Edit View Process Help

Single Mass Analysis
Tolerance = 100.0 PPM / DBE: min = -1.5, max = 100.0
Element prediction: Off
Number of isotope peaks used for i-FIT = 3
Monoisotopic Mass, Even Electron Ions
4294 formula(e) evaluated with 455 results within limits (up to 100 closest results for each mass)
Elements Used:

Mass	Calc. Mass	mDa	PPM	DBE	Formula	i-FIT	i-FIT Norm	Fit Conf %	C	H	N	O	S	Cl	Br	F
504.0263	504.0261	0.2	0.4	24.5	C30 H12 N O2 S Cl F	486.9	16.601	0.00	30	12	1	2	1	1		1
	504.0260	0.3	0.6	13.5	C21 H20 N5 O S Cl Br	472.4	2.031	13.12	21	20	5	1	1	1	1	
	504.0260	0.3	0.6	23.5	C27 H12 N5 Br F	482.2	11.891	0.00	27	12	5				1	1
	504.0266	-0.3	-0.6	20.5	C24 H12 N5 O4 Cl2	485.2	14.869	0.00	24	12	5	4		2		
	504.0269	-0.6	-1.2	17.5	C26 H19 N O3 S Br	481.9	11.508	0.00	26	19	1	3	1		1	
	504.0256	0.7	1.4	9.5	C20 H22 N3 O2 Cl2 Br F	480.6	10.269	0.00	20	22	3	2		2	1	1
	504.0272	-0.9	-1.8	9.5	C18 H21 N5 O2 S Cl Br F	476.7	6.397	0.17	18	21	5	2	1	1	1	1
	504.0253	1.0	2.0	20.5	C25 H13 N5 S Cl2 F	485.0	14.647	0.00	25	13	5		1	2		1
	504.0250	1.3	2.6	38.5	C39 H3 N F	496.8	26.490	0.00	39	3	1					1

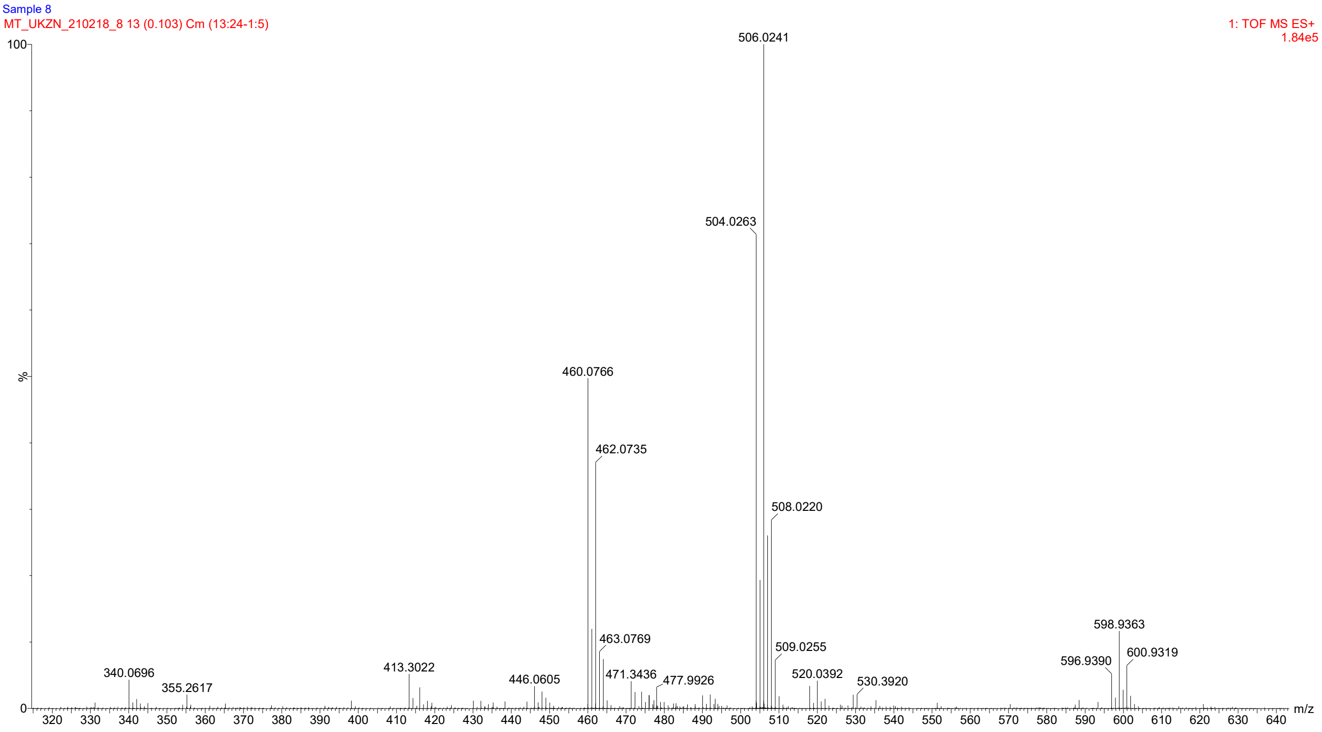

Figure S47: HRMS (ES+) for *1-(6-bromobenzo[d]thiazol-2-yl)-3-(4-((7-chloroquinolin-4-yl)amino)butyl)urea)* (***6p***).

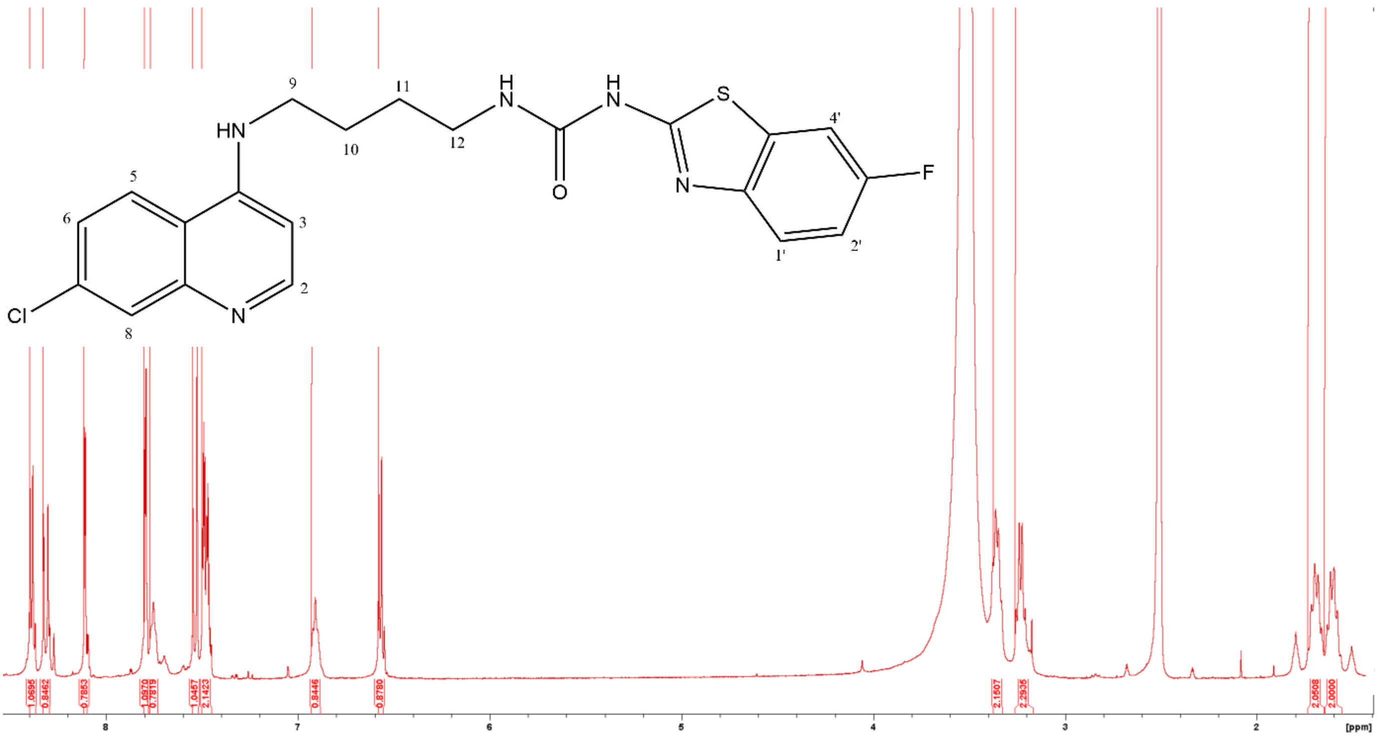

Figure S48: 400 MHz ¹H NMR for *1-(6-fluorobenzo[d]thiazol-2-yl)-3-(4-((7-chloroquinolin-4-yl)amino)butyl)urea* in DMSO-d6 (**6q**).

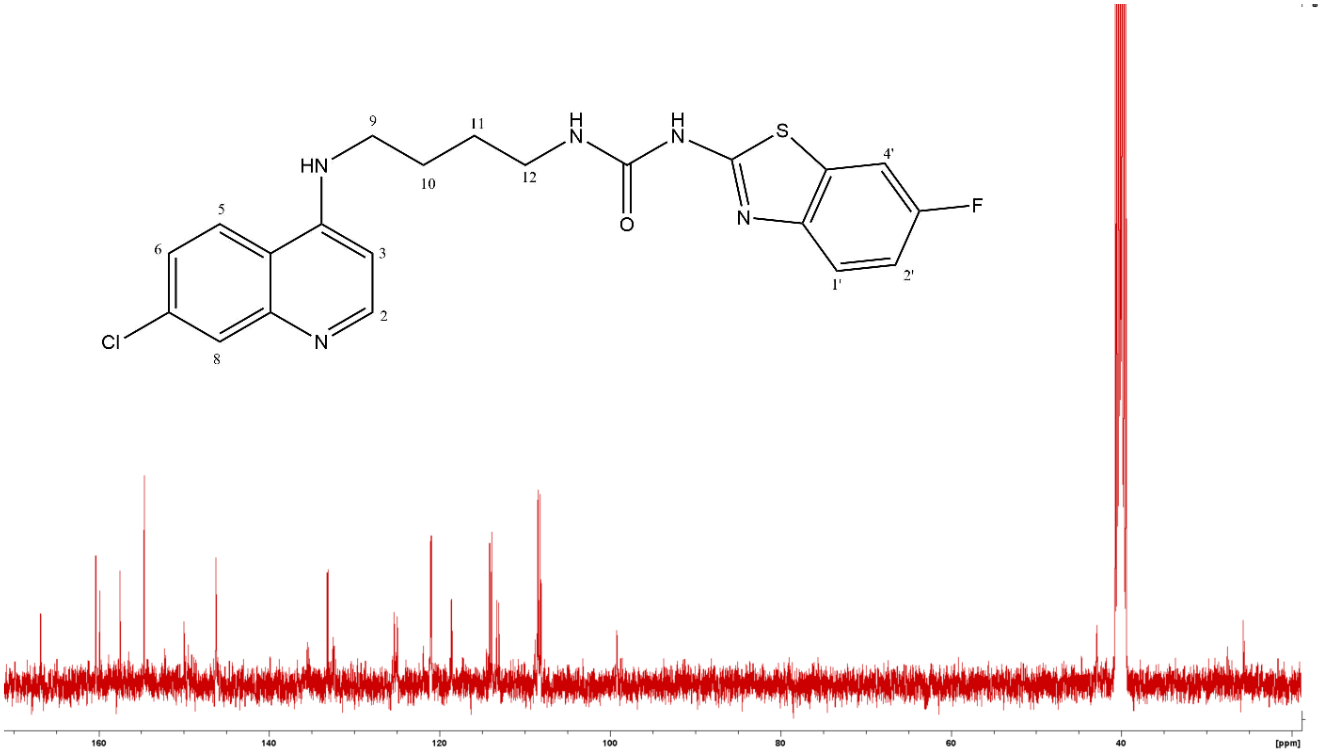

Figure S49: 101 MHz ¹³C NMR for *1-(6-fluorobenzo[d]thiazol-2-yl)-3-(4-((7-chloroquinolin-4-yl)amino)butyl)urea* in DMSO-d6 (**6q**).

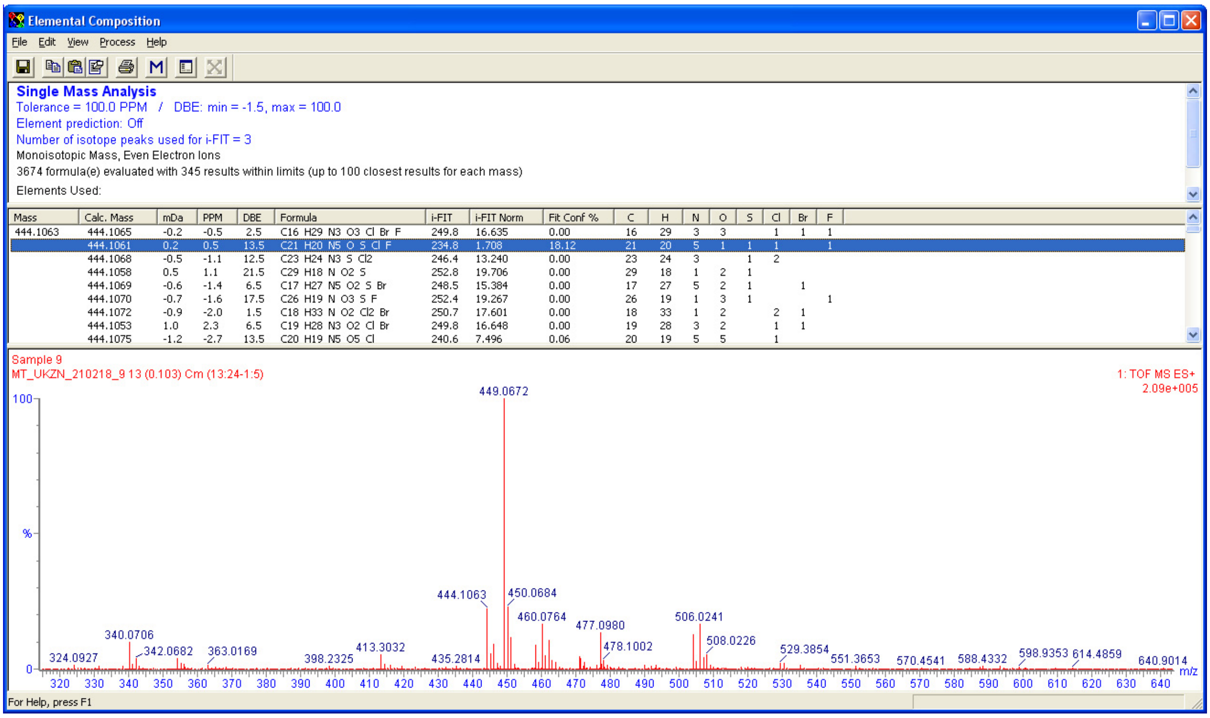

Mass	Calc. Mass	mDa	PPM	DBE	Formula	i-FIT	i-FIT Norm	Fit Conf %	C	H	N	O	S	Cl	Br	F
444.1063	444.1065	-0.2	-0.5	2.5	C16 H29 N3 O3 Cl Br F	249.8	16.635	0.00	16	29	3	3		1	1	1
	444.1061	0.2	0.5	13.5	C21 H20 N5 O S Cl F	234.8	1.708	18.12	21	20	5	1	1	1		1
	444.1068	-0.5	-1.1	12.5	C23 H24 N3 S Cl2	246.4	13.240	0.00	23	24	3		1	2		
	444.1058	0.5	1.1	21.5	C29 H18 N O2 S	252.8	19.706	0.00	29	18	1	2	1			
	444.1069	-0.6	-1.4	6.5	C17 H27 N5 O2 S Br	248.5	15.384	0.00	17	27	5	2	1		1	
	444.1070	-0.7	-1.6	17.5	C26 H19 N O3 S F	252.4	19.267	0.00	26	19	1	3	1			1
	444.1072	-0.9	-2.0	1.5	C18 H33 N O2 Cl2 Br	250.7	17.601	0.00	18	33	1	2		2	1	
	444.1053	1.0	2.3	6.5	C19 H28 N3 O2 Cl Br	249.8	16.648	0.00	19	28	3	2		1	1	
	444.1075	-1.2	-2.7	13.5	C20 H19 N5 O5 Cl	240.6	7.496	0.06	20	19	5	5		1		

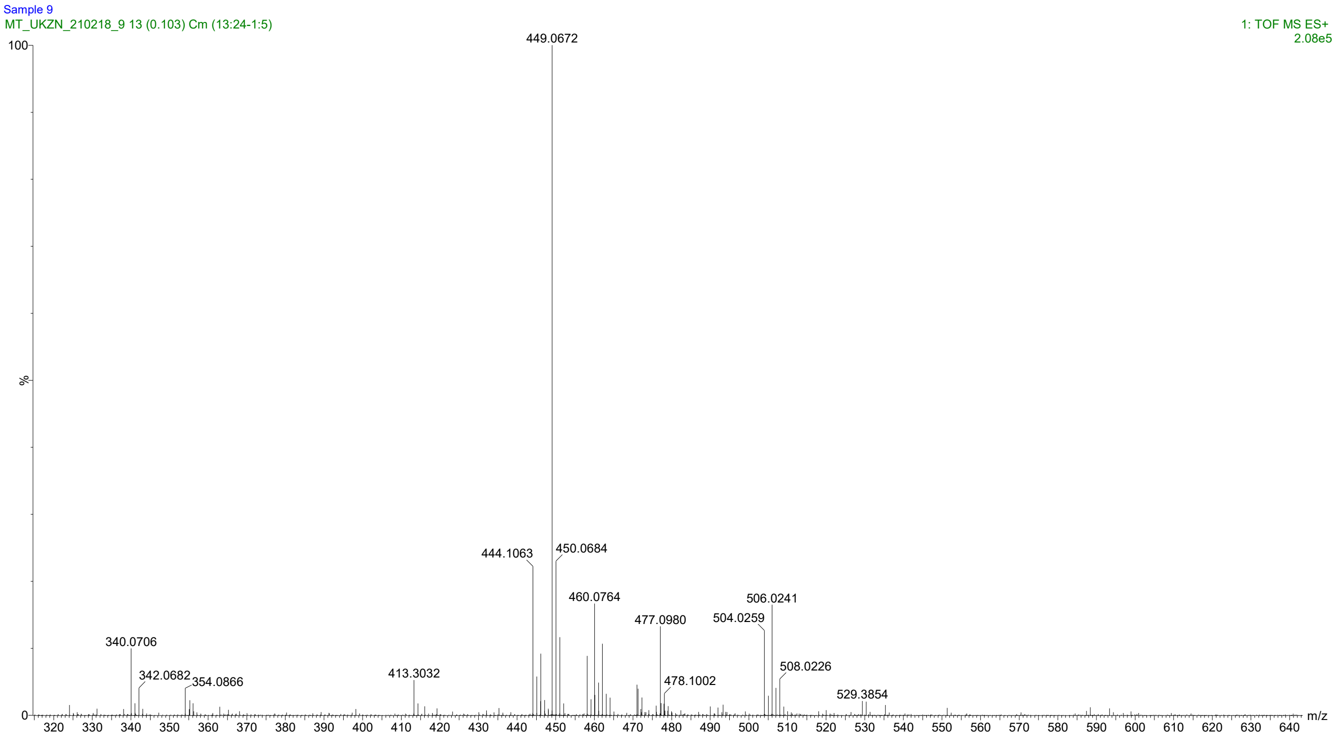

Figure S50: HRMS (ES+) for *1-(6-fluorobenzo[d]thiazol-2-yl)-3-(4-((7-chloroquinolin-4-yl)amino)butyl)urea* (**6q**).

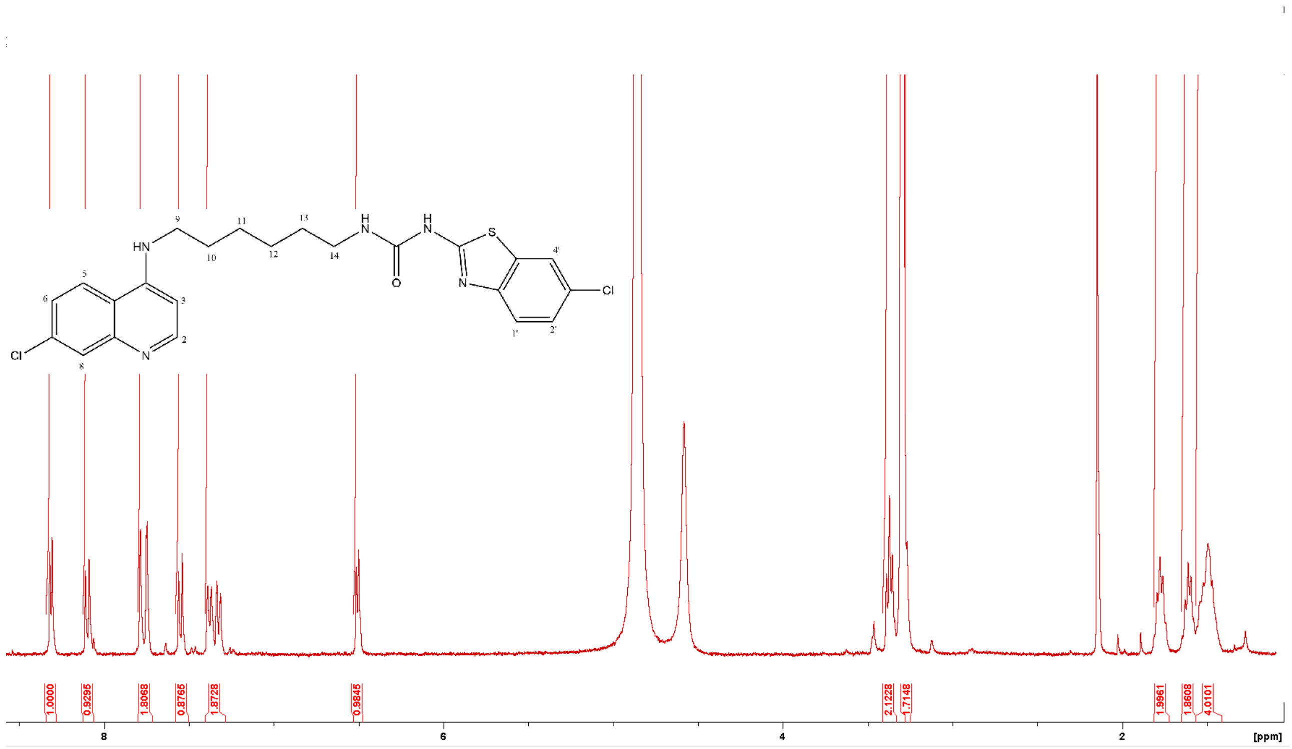

Figure S51: 400 MHz ^{1}H-NMR of *1-(6-chlorobenzo[d]thiazol-2-yl)-3-(6-((7-chloroquinolin-4-yl)amino)hexyl)urea in MeOH-d4* (***6r***).

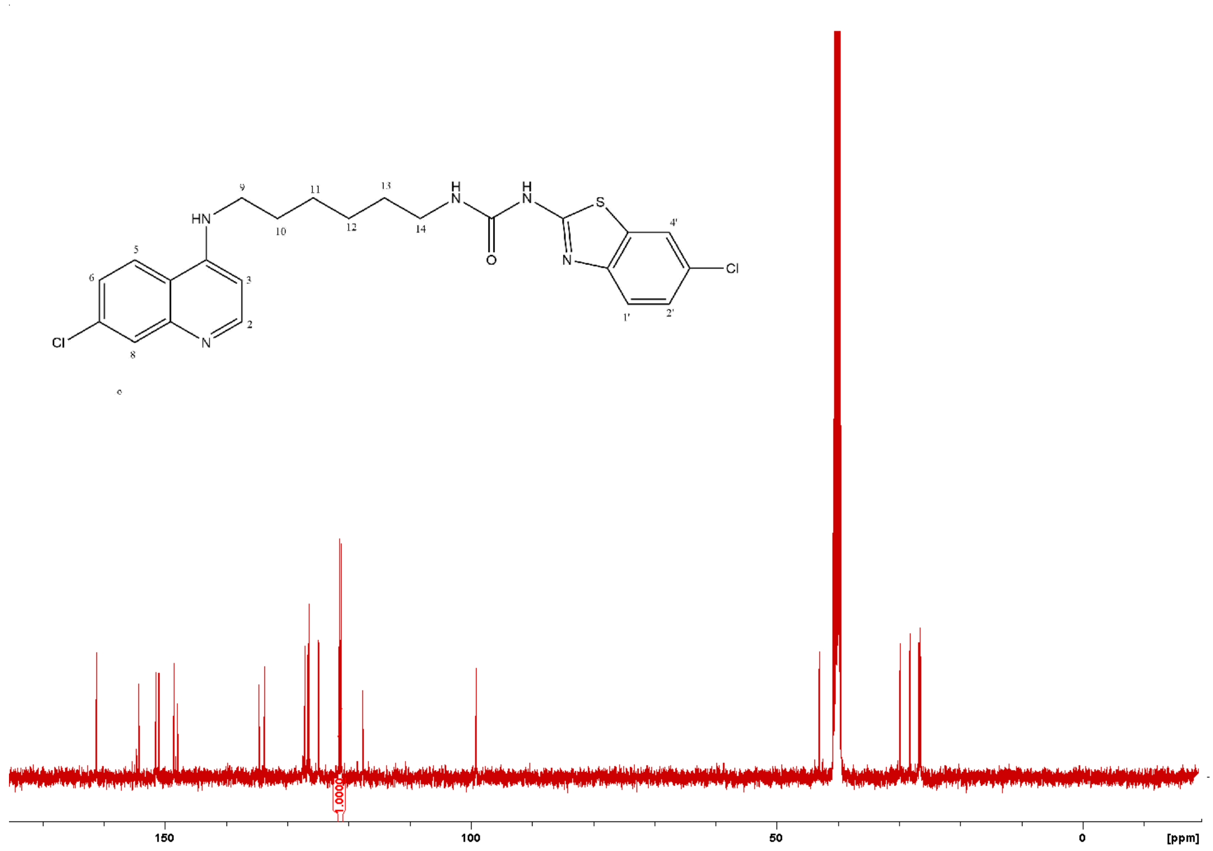

Figure S52: 101 MHz ^{13}C-NMR of *1-(6-chlorobenzo[d]thiazol-2-yl)-3-(6-((7-chloroquinolin-4-yl)amino)hexyl)urea* in DMSO-d6 (***6r***).

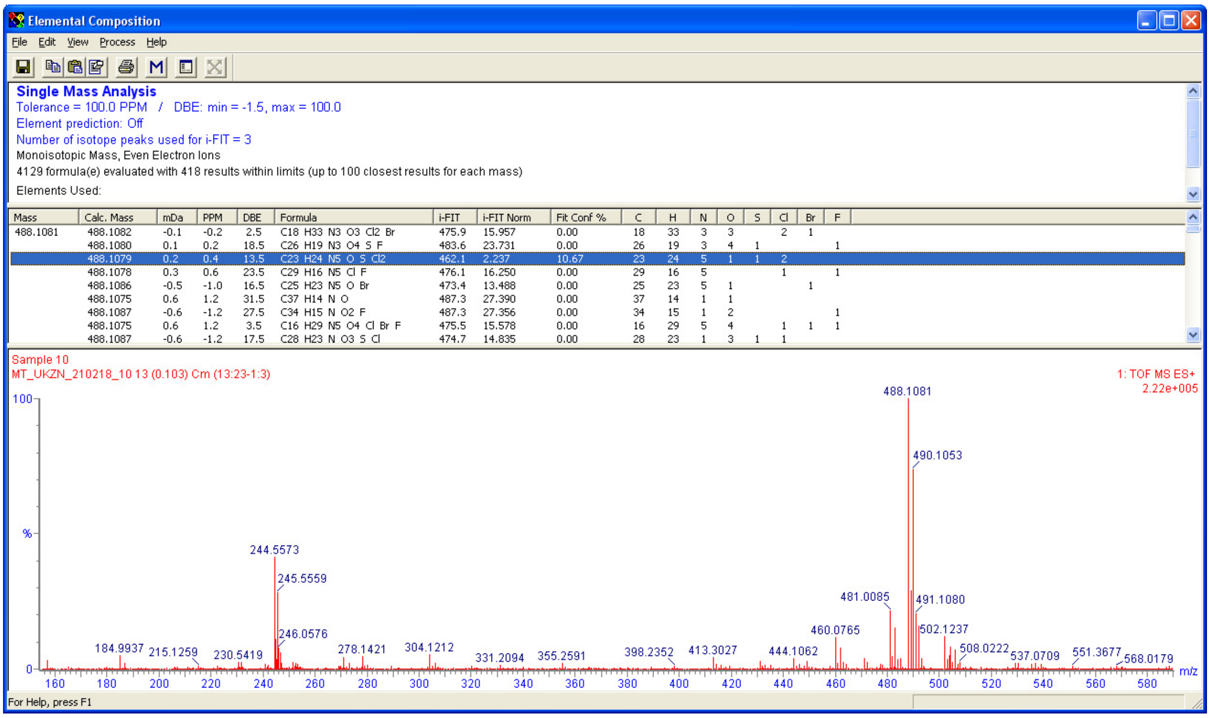

Elemental Composition

File Edit View Process Help

Single Mass Analysis
Tolerance = 100.0 PPM / DBE: min = -1.5, max = 100.0
Element prediction: Off
Number of isotope peaks used for i-FIT = 3
Monoisotopic Mass, Even Electron Ions
4129 formula(e) evaluated with 418 results within limits (up to 100 closest results for each mass)
Elements Used:

Mass	Calc. Mass	mDa	PPM	DBE	Formula	i-FIT	i-FIT Norm	Fit Conf %	C	H	N	O	S	Cl	Br	F
488.1081	488.1082	-0.1	-0.2	2.5	C18 H33 N3 O3 Cl2 Br	475.9	15.957	0.00	18	33	3	3		2	1	
	488.1080	0.1	0.2	18.5	C26 H19 N3 O4 S F	483.6	23.731	0.00	26	19	3	4	1			1
	488.1079	0.2	0.4	13.5	C23 H24 N5 O S Cl2	462.1	2.237	10.67	23	24	5	1	1	2		
	488.1078	0.3	0.6	23.5	C29 H16 N5 Cl F	476.1	16.250	0.00	29	16	5			1		1
	488.1086	-0.5	-1.0	16.5	C25 H23 N5 O Br	473.4	13.488	0.00	25	23	5	1			1	
	488.1075	0.6	1.2	31.5	C37 H14 N O	487.3	27.390	0.00	37	14	1	1				
	488.1087	-0.6	-1.2	27.5	C34 H15 N O2 F	487.3	27.356	0.00	34	15	1	2				1
	488.1075	0.6	1.2	3.5	C16 H29 N5 O4 Cl Br F	475.5	15.578	0.00	16	29	5	4		1	1	1
	488.1087	-0.6	-1.2	17.5	C28 H23 N O3 S Cl	474.7	14.835	0.00	28	23	1	3	1	1		

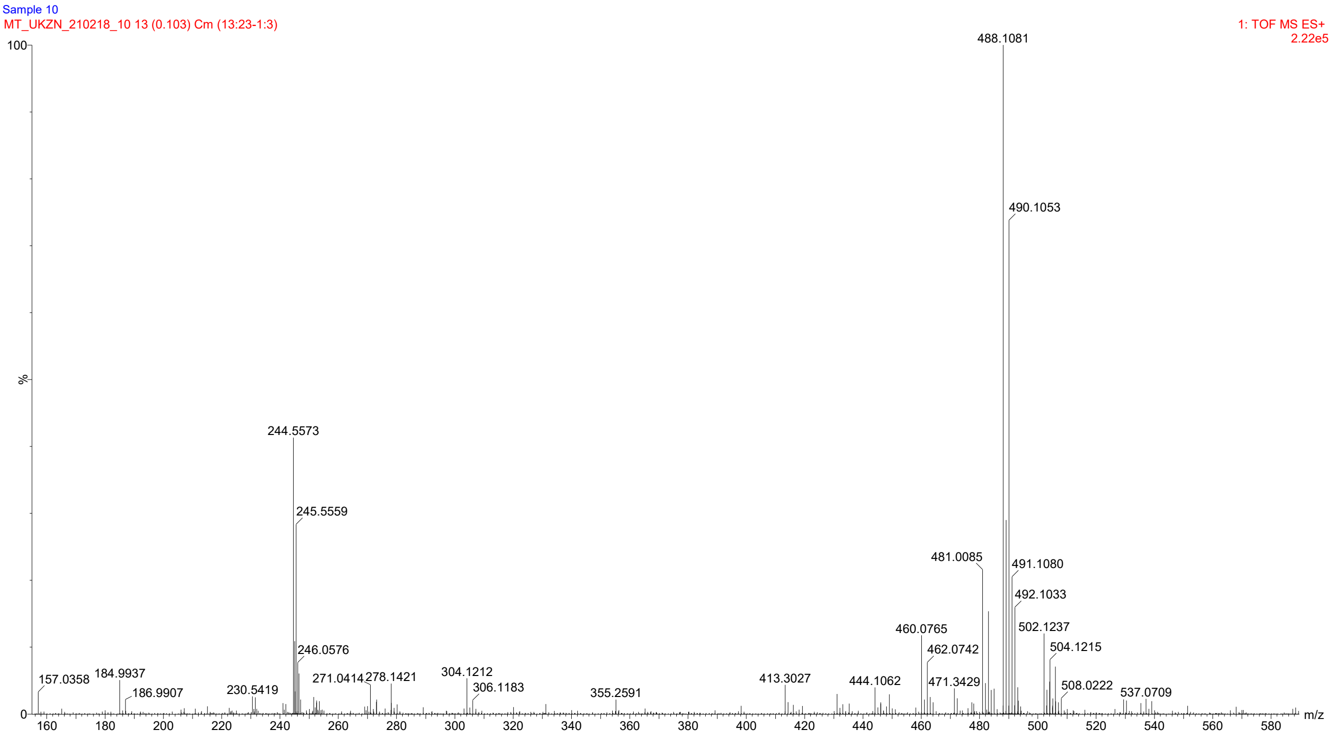

Figure S53: HRMS (ES+) for *1-(6-chlorobenzo[d]thiazol-2-yl)-3-(6-((7-chloroquinolin-4-yl)amino)hexyl)urea* (**6r**).

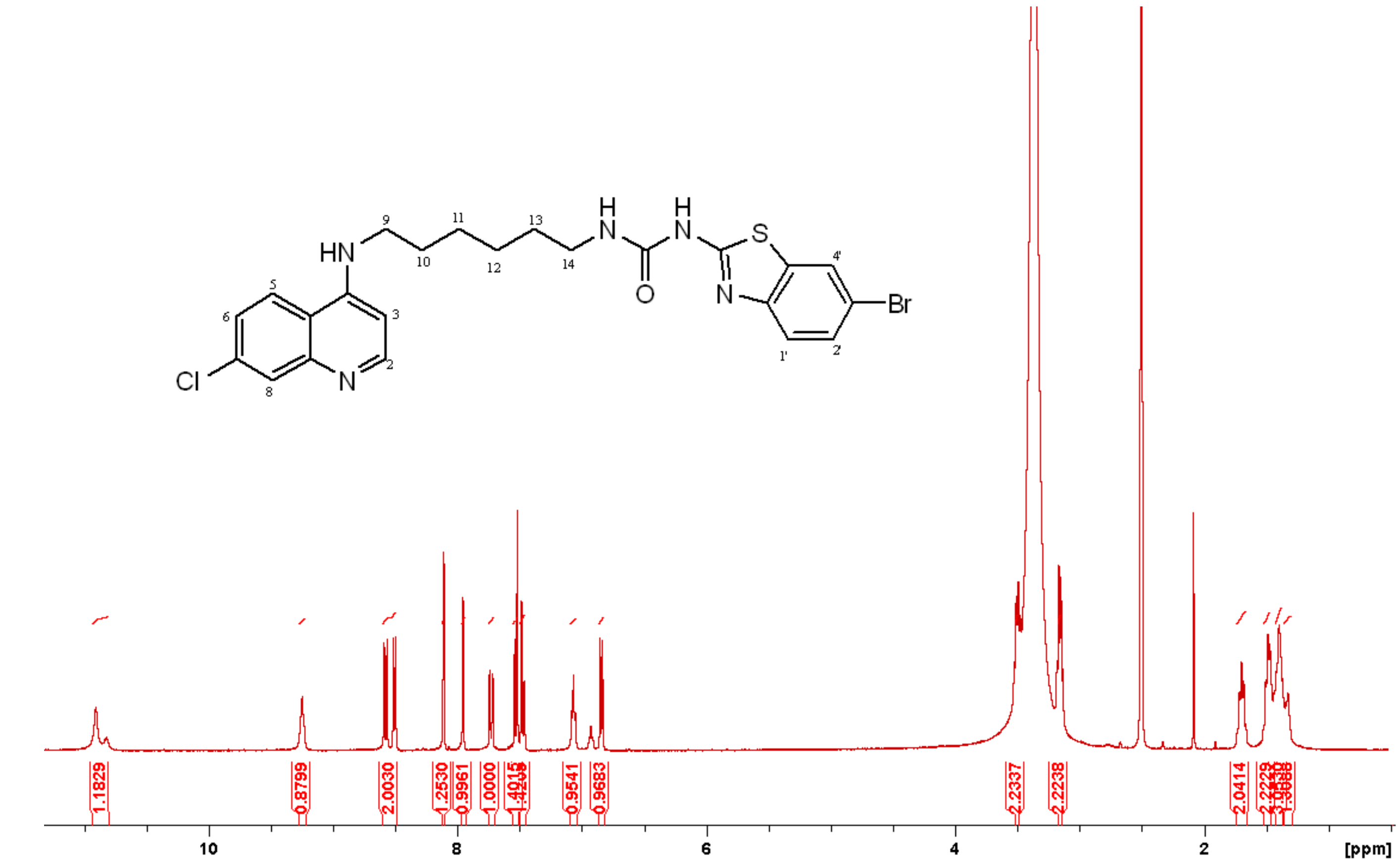

Figure S54: 400 MHz ^{1}H-NMR of *1-(6-bromobenzo[d]thiazol-2-yl)-3-(6-((7-chloroquinolin-4-yl)amino)hexyl)urea in DMSO-d6 (**6s**).*

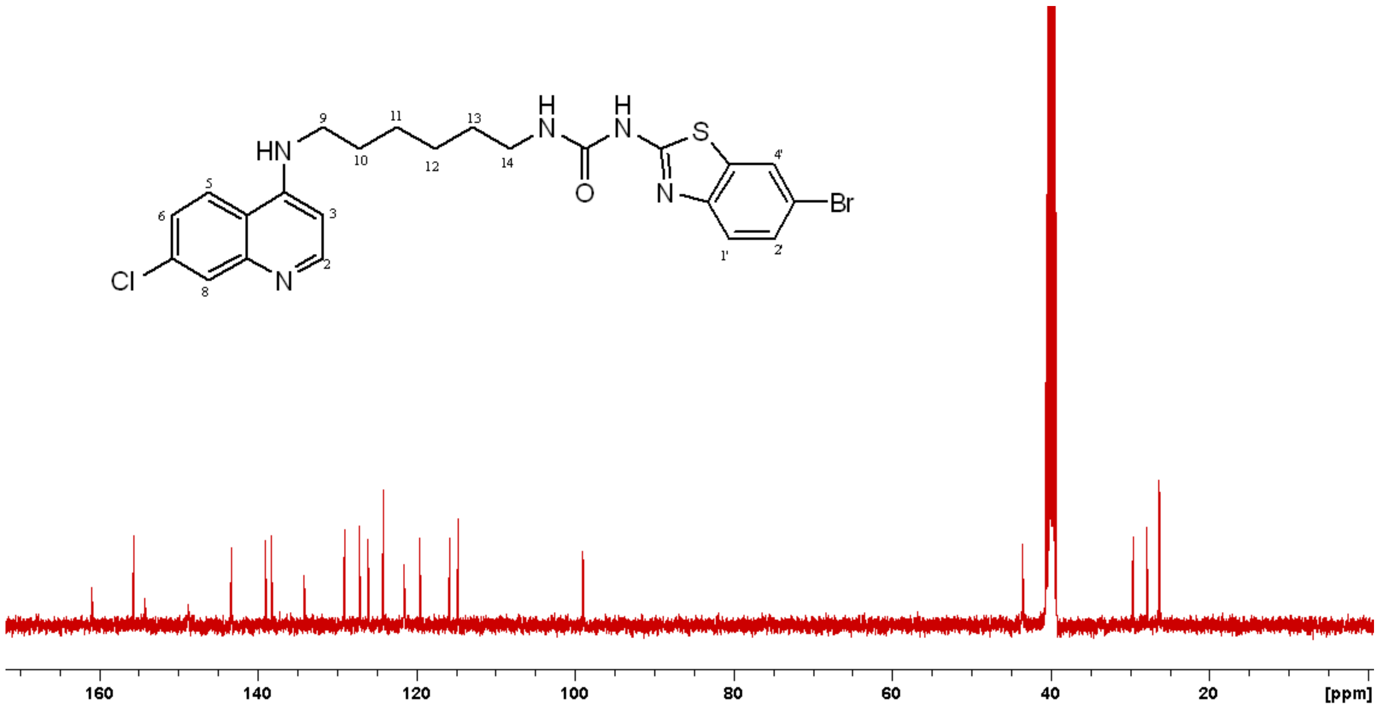

Figure S55: 101 MHz ^{13}C-NMR of *1-(6-bromobenzo[d]thiazol-2-yl)-3-(6-((7-chloroquinolin-4-yl)amino)hexyl)urea (**6s**).*

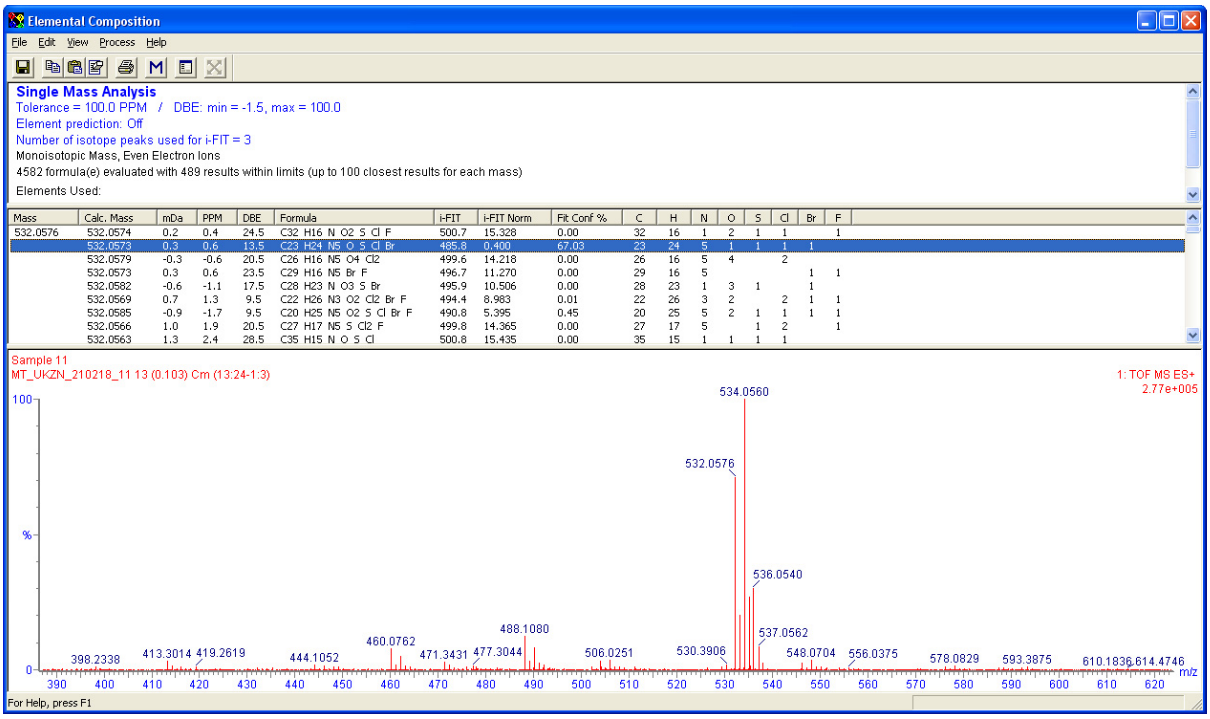

Mass	Calc. Mass	mDa	PPM	DBE	Formula	i-FIT	i-FIT Norm	Fit Conf %	C	H	N	O	S	Cl	Br	F
532.0576	532.0574	0.2	0.4	24.5	C32 H16 N O2 S Cl F	500.7	15.328	0.00	32	16	1	2	1	1		1
	532.0573	0.3	0.6	13.5	C23 H24 N5 O S Cl Br	485.8	0.400	67.03	23	24	5	1	1	1	1	
	532.0579	-0.3	-0.6	20.5	C26 H16 N5 O4 Cl2	499.6	14.218	0.00	26	16	5	4		2		
	532.0573	0.3	0.6	23.5	C29 H16 N5 Br F	496.7	11.270	0.00	29	16	5				1	1
	532.0582	-0.6	-1.1	17.5	C28 H23 N O3 S Br	495.9	10.506	0.00	28	23	1	3	1		1	
	532.0569	0.7	1.3	9.5	C22 H26 N3 O2 Cl2 Br F	494.4	8.983	0.01	22	26	3	2		2	1	1
	532.0585	-0.9	-1.7	9.5	C20 H25 N5 O2 S Cl Br F	490.8	5.395	0.45	20	25	5	2	1	1	1	1
	532.0566	1.0	1.9	20.5	C27 H17 N5 S Cl2 F	499.8	14.365	0.00	27	17	5		1	2		1
	532.0563	1.3	2.4	28.5	C35 H15 N O S Cl	500.8	15.435	0.00	35	15	1	1	1	1		

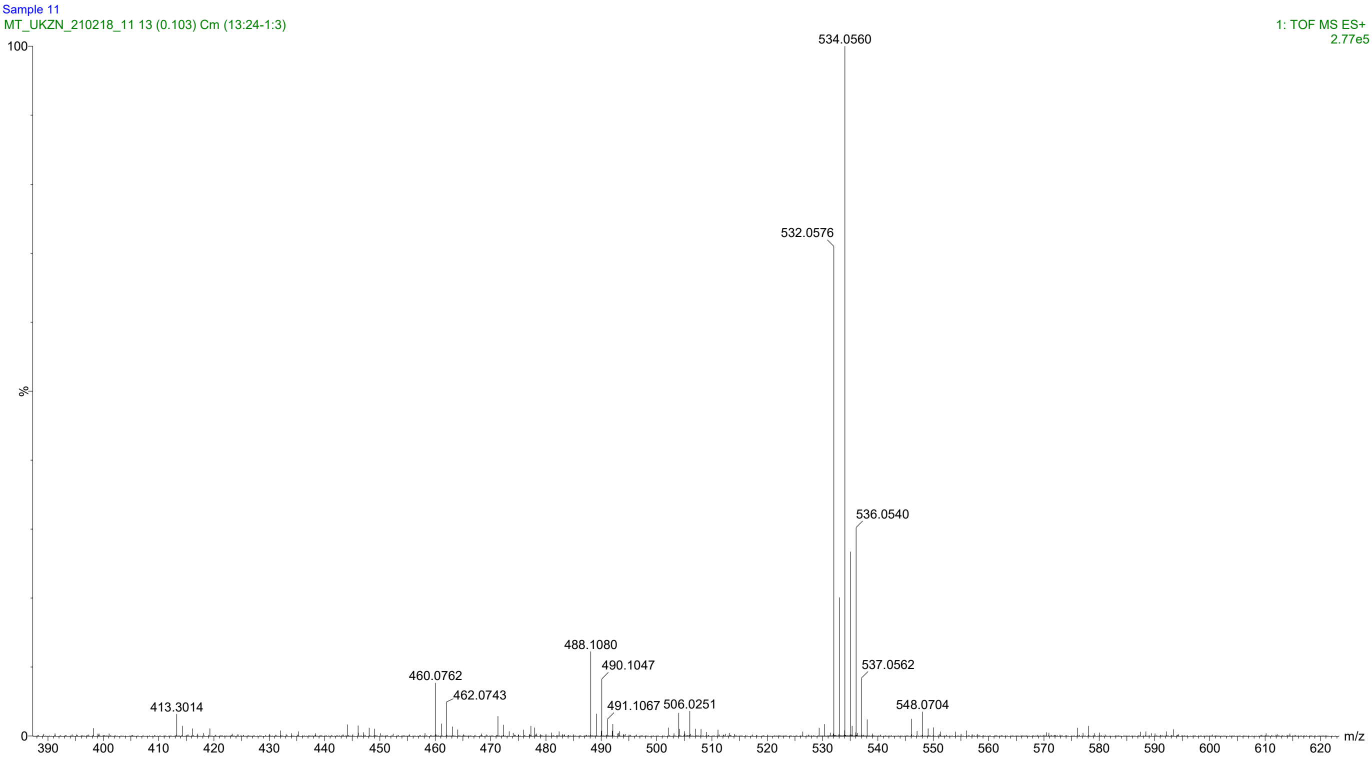

Figure S56: HRMS (ES$^+$) for *1-(6-bromobenzo[d]thiazol-2-yl)-3-(6-((7-chloroquinolin-4-yl)amino)hexyl)urea (**6s**).*

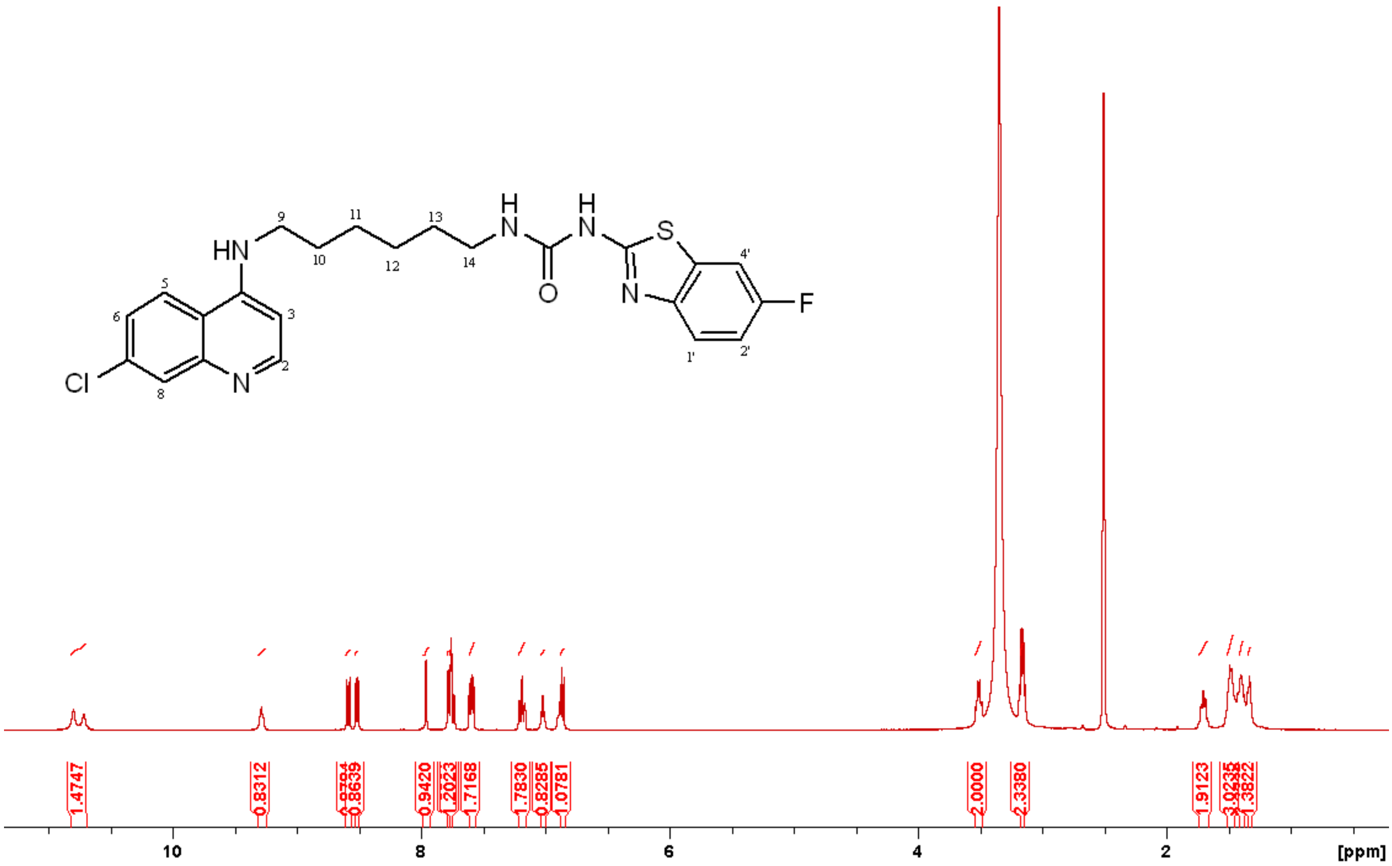

Figure S57: 400 MHz ¹H-NMR of *1-(6-fluorobenzo[d]thiazol-2-yl)-3-(6-((7-chloroquinolin-4-yl)amino)hexyl)urea* in DMSO-d6 (***6t***).

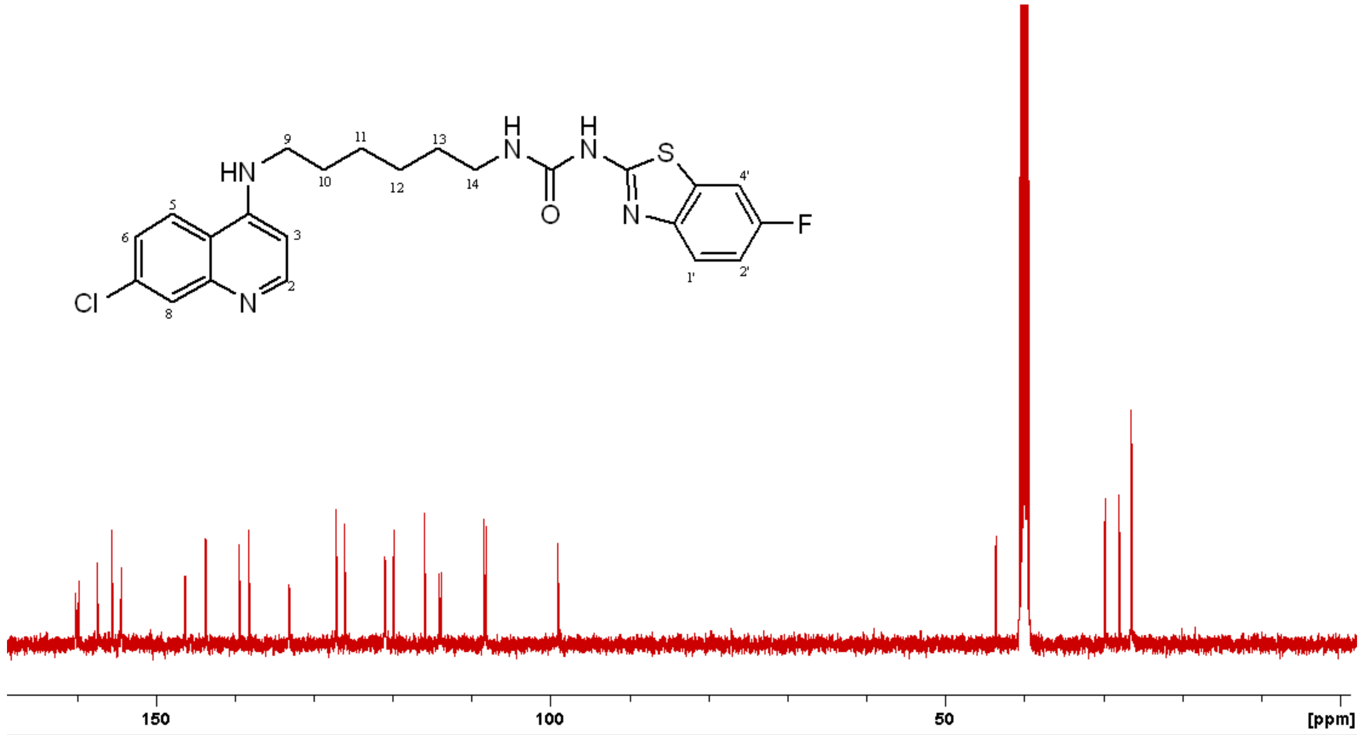

Figure S58: 101 MHz ¹³C-NMR of *1-(6-chlorobenzo[d]thiazol-2-yl)-3-(6-((7-chloroquinolin-4-yl)amino)hexyl)urea* in DMSO-d6 (***6t***).

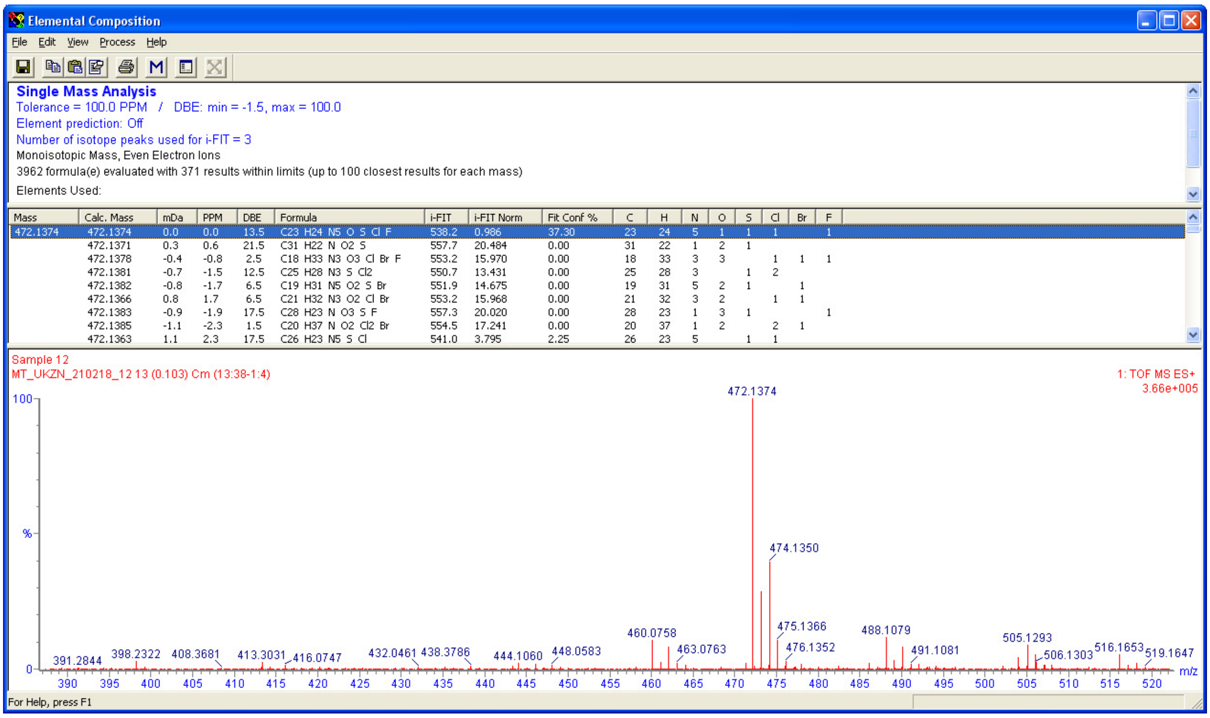
Elemental Composition

File Edit View Process Help

Single Mass Analysis

Tolerance = 100.0 PPM / DBE: min = -1.5, max = 100.0

Element prediction: Off

Number of isotope peaks used for i-FIT = 3

Monoisotopic Mass, Even Electron Ions

3962 formula(e) evaluated with 371 results within limits (up to 100 closest results for each mass)

Elements Used:

Mass	Calc. Mass	mDa	PPM	DBE	Formula	i-FIT	i-FIT Norm	Fit Conf %	C	H	N	O	S	Cl	Br	F
472.1374	472.1374	0.0	0.0	13.5	C23 H24 N5 O S Cl F	538.2	0.986	37.30	23	24	5	1	1	1		1
	472.1371	0.3	0.6	21.5	C31 H22 N O2 S	557.7	20.484	0.00	31	22	1	2	1			
	472.1378	-0.4	-0.8	2.5	C18 H33 N3 O3 Cl Br F	553.2	15.970	0.00	18	33	3	3		1	1	1
	472.1381	-0.7	-1.5	12.5	C25 H28 N3 S Cl2	550.7	13.431	0.00	25	28	3		1	2		
	472.1382	-0.8	-1.7	6.5	C19 H31 N5 O2 S Br	551.9	14.675	0.00	19	31	5	2	1		1	
	472.1366	0.8	1.7	6.5	C21 H32 N3 O2 Cl Br	553.2	15.968	0.00	21	32	3	2		1	1	
	472.1383	-0.9	-1.9	17.5	C28 H23 N O3 S F	557.3	20.020	0.00	28	23	1	3	1			1
	472.1385	-1.1	-2.3	1.5	C20 H37 N O2 Cl2 Br	554.5	17.241	0.00	20	37	1	2		2	1	
	472.1363	1.1	2.3	17.5	C26 H23 N5 S Cl	541.0	3.795	2.25	26	23	5		1	1		

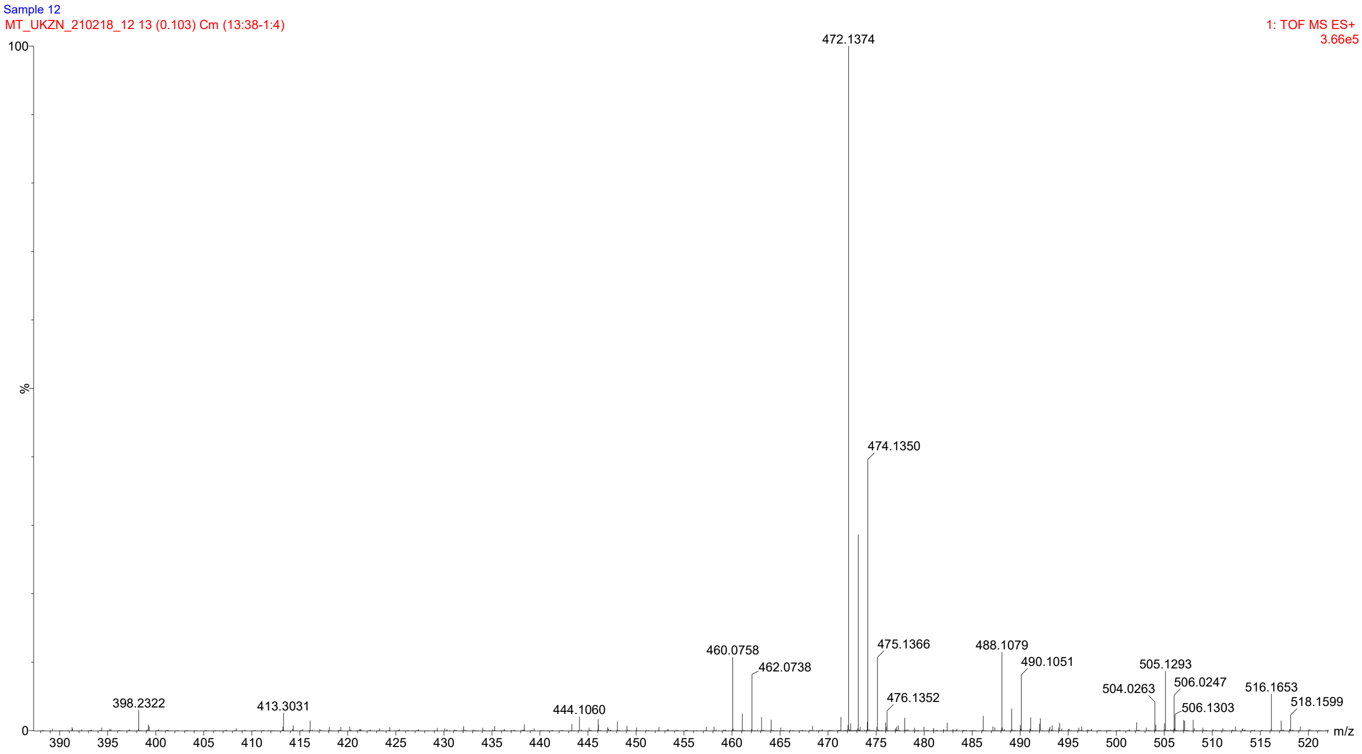

Figure S59: HRMS (ES^{+}) for *1-(6-((7-chloroquinolin-4-yl)amino)hexyl)-3-(6-fluorobenzo[d]thiazol-2-yl)urea* (***6t***).

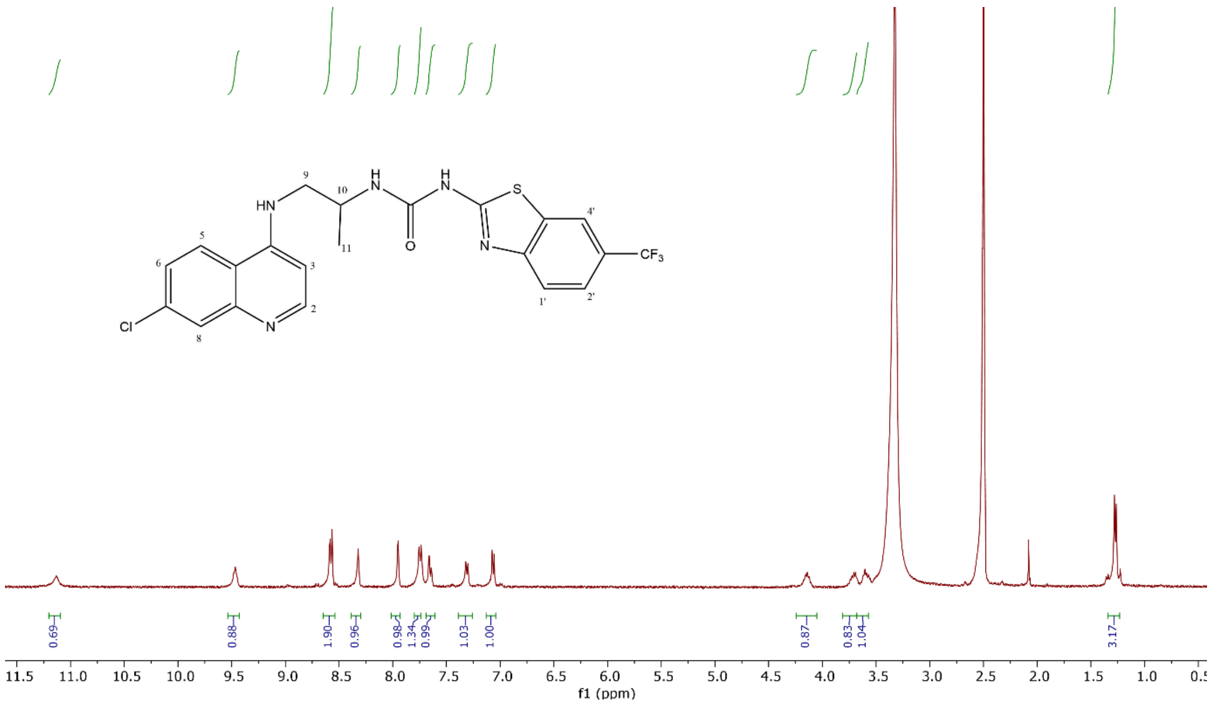

Figure S60: 400 MHz ^{1}H-NMR of *1-(1-((7-chloroquinolin-4-yl)amino)propan-2-yl)-3-(6-(trifluoromethyl)benzo[d]thiazol-2-yl)urea* in DMSO-d6 (***6u***).

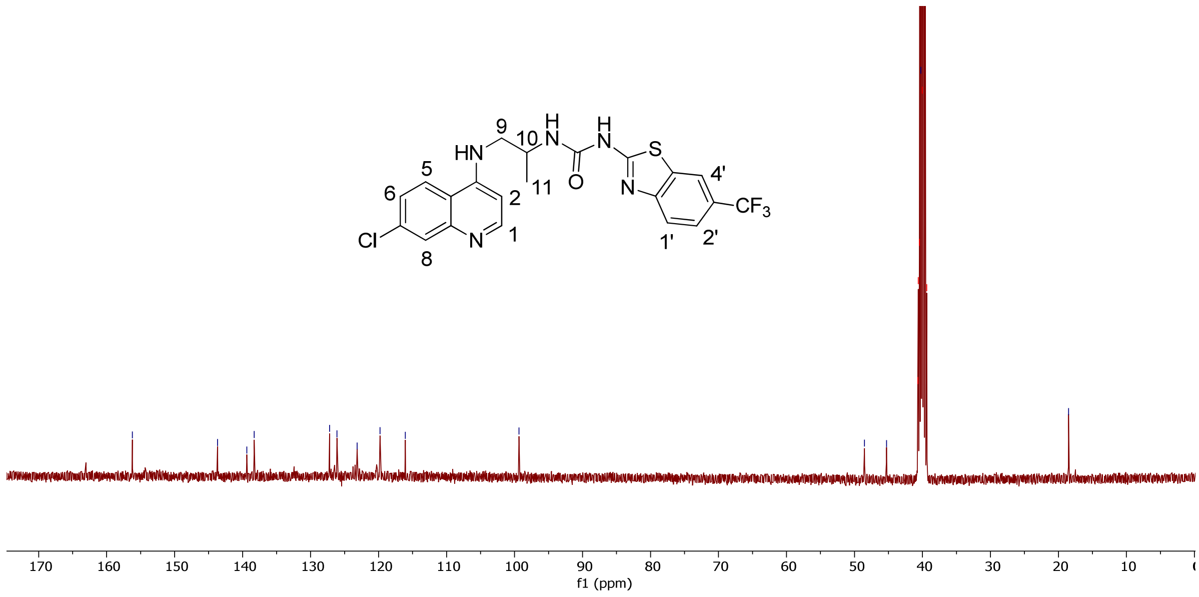

Figure S61: 101 MHz ^{13}C-NMR of *1-(1-((7-chloroquinolin-4-yl)amino)propan-2-yl)-3-(6-(trifluoromethyl)benzo[d]thiazol-2-yl)urea* in DMSO-d6 (***6u***).

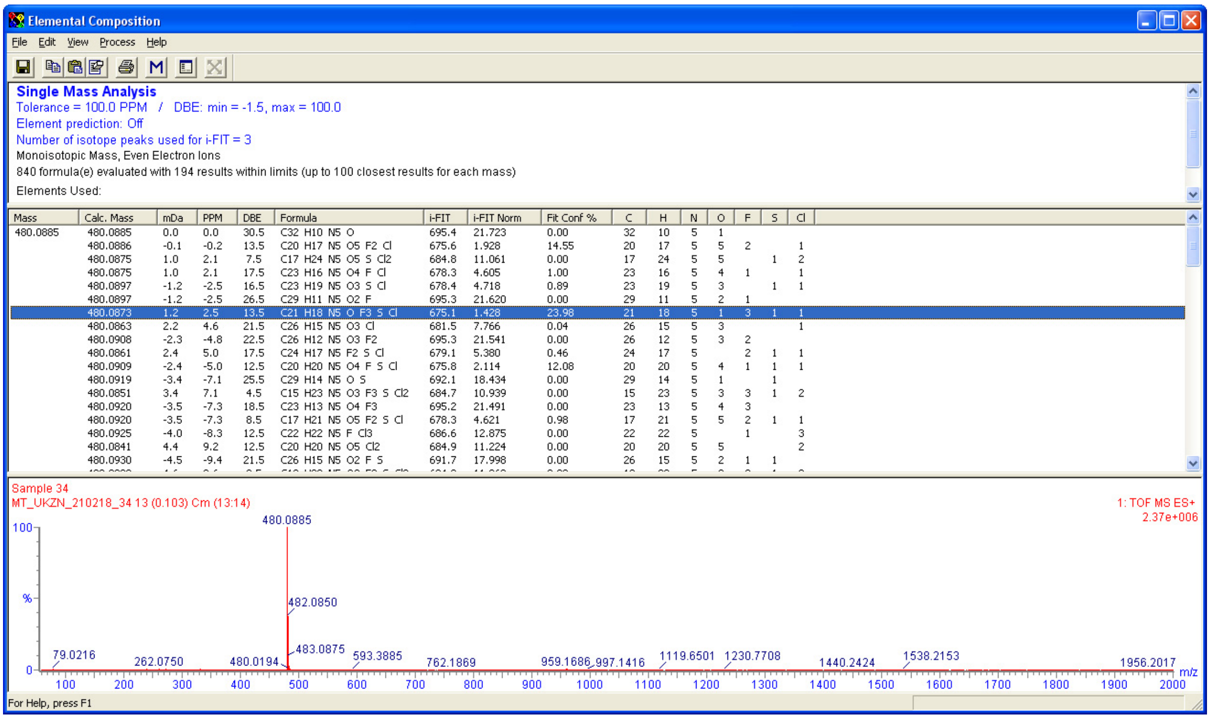
Elemental Composition

File Edit View Process Help

Single Mass Analysis

Tolerance = 100.0 PPM / DBE: min = -1.5, max = 100.0

Element prediction: Off

Number of isotope peaks used for i-FIT = 3

Monoisotopic Mass, Even Electron Ions

840 formula(e) evaluated with 194 results within limits (up to 100 closest results for each mass)

Elements Used:

Mass	Calc. Mass	mDa	PPM	DBE	Formula	i-FIT	i-FIT Norm	Fit Conf %	C	H	N	O	F	S	Cl
480.0885	480.0885	0.0	0.0	30.5	C32 H10 N5 O	695.4	21.723	0.00	32	10	5	1			
	480.0886	-0.1	-0.2	13.5	C20 H17 N5 O5 F2 Cl	675.6	1.928	14.55	20	17	5	5	2		1
	480.0875	1.0	2.1	7.5	C17 H24 N5 O5 S Cl2	684.8	11.061	0.00	17	24	5	5		1	2
	480.0875	1.0	2.1	17.5	C23 H16 N5 O4 F Cl	678.3	4.605	1.00	23	16	5	4	1		1
	480.0897	-1.2	-2.5	16.5	C23 H19 N5 O3 S Cl	678.4	4.718	0.89	23	19	5	3		1	1
	480.0897	-1.2	-2.5	26.5	C29 H11 N5 O2 F	695.3	21.620	0.00	29	11	5	2	1		
	480.0873	1.2	2.5	13.5	C21 H18 N5 O F3 S Cl	675.1	1.428	23.98	21	18	5	1	3	1	1
	480.0863	2.2	4.6	21.5	C26 H15 N5 O3 Cl	681.5	7.766	0.04	26	15	5	3			1
	480.0908	-2.3	-4.8	22.5	C26 H12 N5 O3 F2	695.3	21.541	0.00	26	12	5	3	2		
	480.0861	2.4	5.0	17.5	C24 H17 N5 F2 S Cl	679.1	5.380	0.46	24	17	5		2	1	1
	480.0909	-2.4	-5.0	12.5	C20 H20 N5 O4 F S Cl	675.8	2.114	12.08	20	20	5	4	1	1	1
	480.0919	-3.4	-7.1	25.5	C29 H14 N5 O S	692.1	18.434	0.00	29	14	5	1		1	
	480.0851	3.4	7.1	4.5	C15 H23 N5 O3 F3 S Cl2	684.7	10.939	0.00	15	23	5	3	3	1	2
	480.0920	-3.5	-7.3	18.5	C23 H13 N5 O4 F3	695.2	21.491	0.00	23	13	5	4	3		
	480.0920	-3.5	-7.3	8.5	C17 H21 N5 O5 F2 S Cl	678.3	4.621	0.98	17	21	5	5	2	1	1
	480.0925	-4.0	-8.3	12.5	C22 H22 N5 F Cl3	686.6	12.875	0.00	22	22	5		1		3
	480.0841	4.4	9.2	12.5	C20 H20 N5 O5 Cl2	684.9	11.224	0.00	20	20	5	5			2
	480.0930	-4.5	-9.4	21.5	C26 H15 N5 O2 F S	691.7	17.998	0.00	26	15	5	2	1	1	

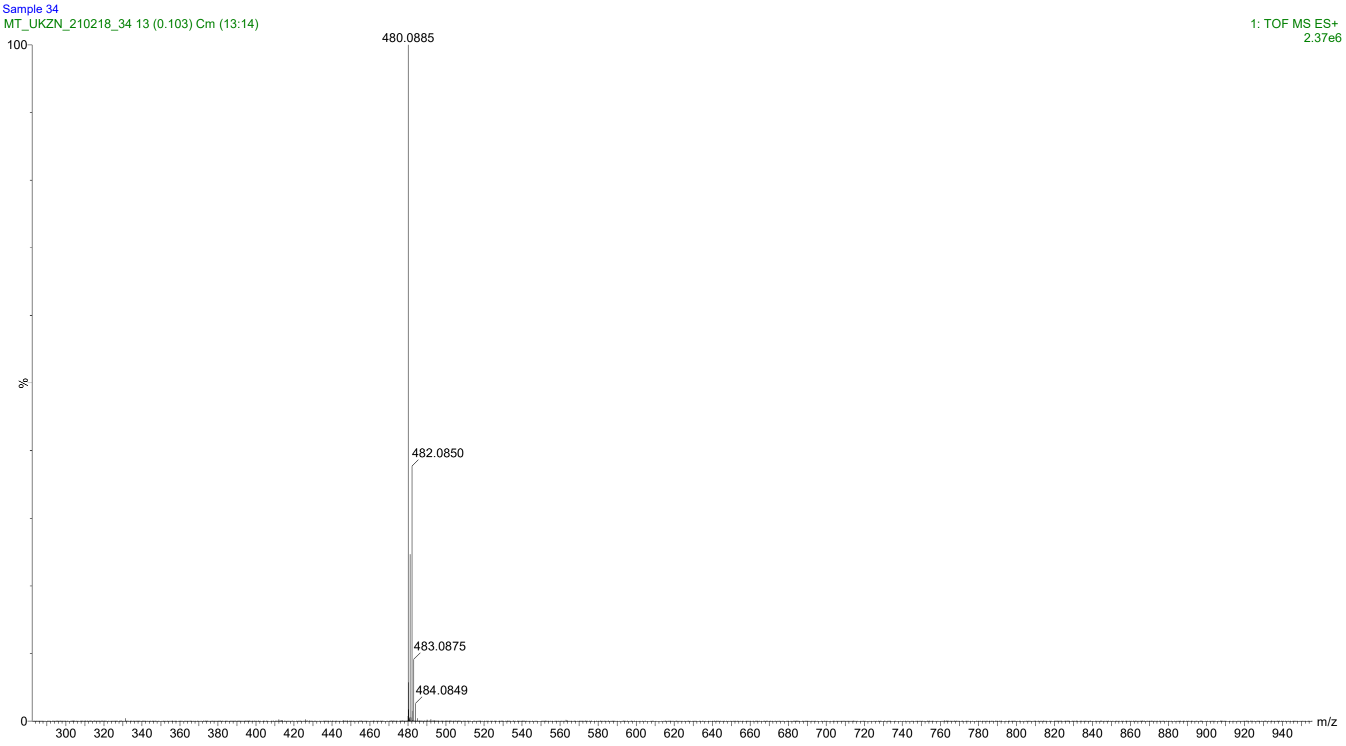

Figure S62: HRMS (ES^+) for *1-(1-((7-chloroquinolin-4-yl)amino)propan-2-yl)-3-(6-(trifluoromethyl)benzo[d]thiazol-2-yl)urea* (**6u**).

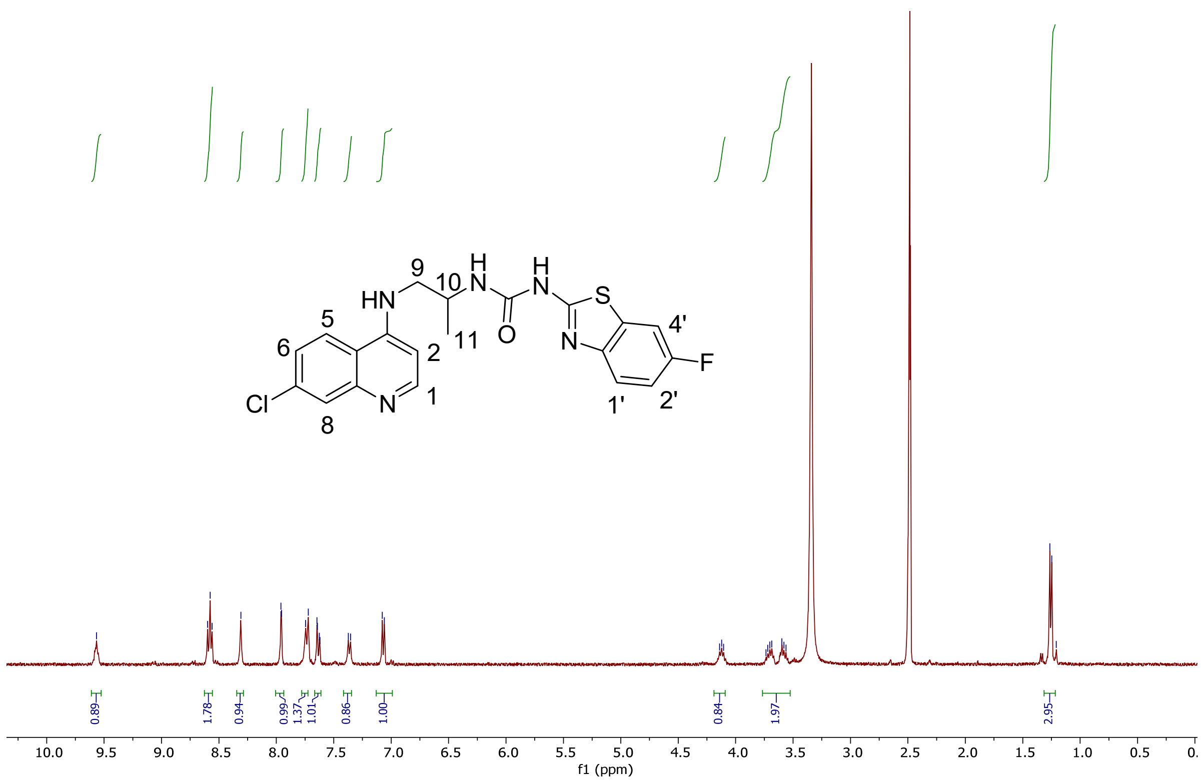

Figure S63: 400 MHz ^{1}H-NMR of *1-(1-((7-chloroquinolin-4-yl)amino)propan-2-yl)-3-(6-fluorobenzo[d]thiazol-2-yl)urea* in DMSO-d6 ***(6v).***

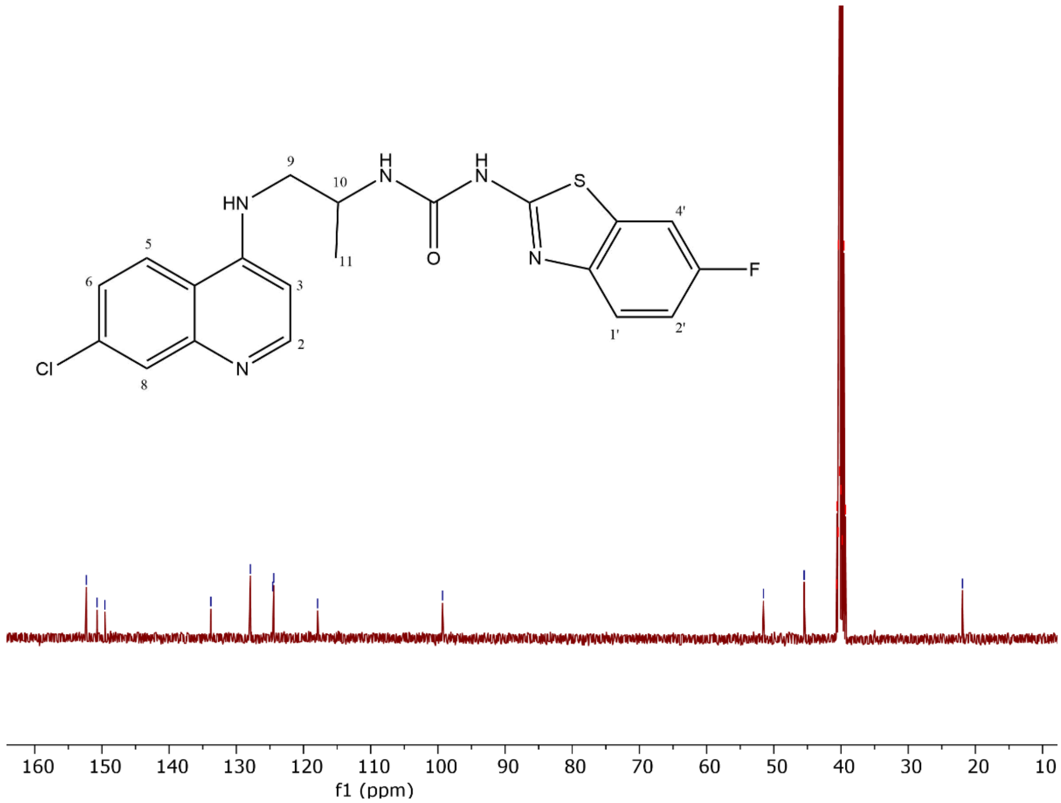

Figure S64: 101 MHz ^{13}C-NMR of *1-(1-((7-chloroquinolin-4-yl)amino)propan-2-yl)-3-(6-fluorobenzo[d]thiazol-2-yl)urea* in DMSO-d6 ***(6v).***

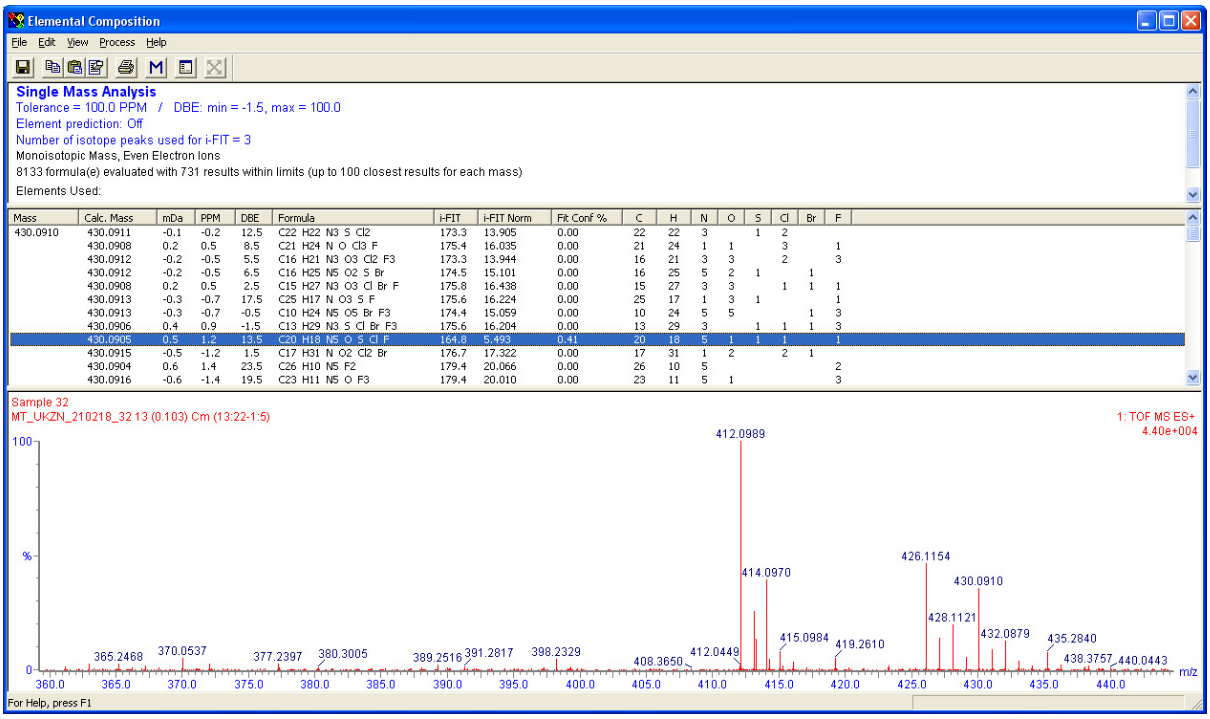

Mass	Calc. Mass	mDa	PPM	DBE	Formula	i-FIT	i-FIT Norm	Fit Conf %	C	H	N	O	S	Cl	Br	F
430.0910	430.0911	-0.1	-0.2	12.5	C22 H22 N3 S Cl2	173.3	13.905	0.00	22	22	3		1	2		
	430.0908	0.2	0.5	8.5	C21 H24 N O Cl3 F	175.4	16.035	0.00	21	24	1	1		3		1
	430.0912	-0.2	-0.5	5.5	C16 H21 N3 O3 Cl2 F3	173.3	13.944	0.00	16	21	3	3		2		3
	430.0912	-0.2	-0.5	6.5	C16 H25 N5 O2 S Br	174.5	15.101	0.00	16	25	5	2	1		1	
	430.0908	0.2	0.5	2.5	C15 H27 N3 O3 Cl Br F	175.8	16.438	0.00	15	27	3	3		1	1	1
	430.0913	-0.3	-0.7	17.5	C25 H17 N O3 S F	175.6	16.224	0.00	25	17	1	3	1			1
	430.0913	-0.3	-0.7	-0.5	C10 H24 N5 O5 Br F3	174.4	15.059	0.00	10	24	5	5			1	3
	430.0906	0.4	0.9	-1.5	C13 H29 N3 S Cl Br F3	175.6	16.204	0.00	13	29	3		1	1	1	3
	430.0905	0.5	1.2	13.5	C20 H18 N5 O S Cl F	164.8	5.493	0.41	20	18	5	1	1	1		1
	430.0915	-0.5	-1.2	1.5	C17 H31 N O2 Cl2 Br	176.7	17.322	0.00	17	31	1	2		2	1	
	430.0904	0.6	1.4	23.5	C26 H10 N5 F2	179.4	20.066	0.00	26	10	5					2
	430.0916	-0.6	-1.4	19.5	C23 H11 N5 O F3	179.4	20.010	0.00	23	11	5	1				3

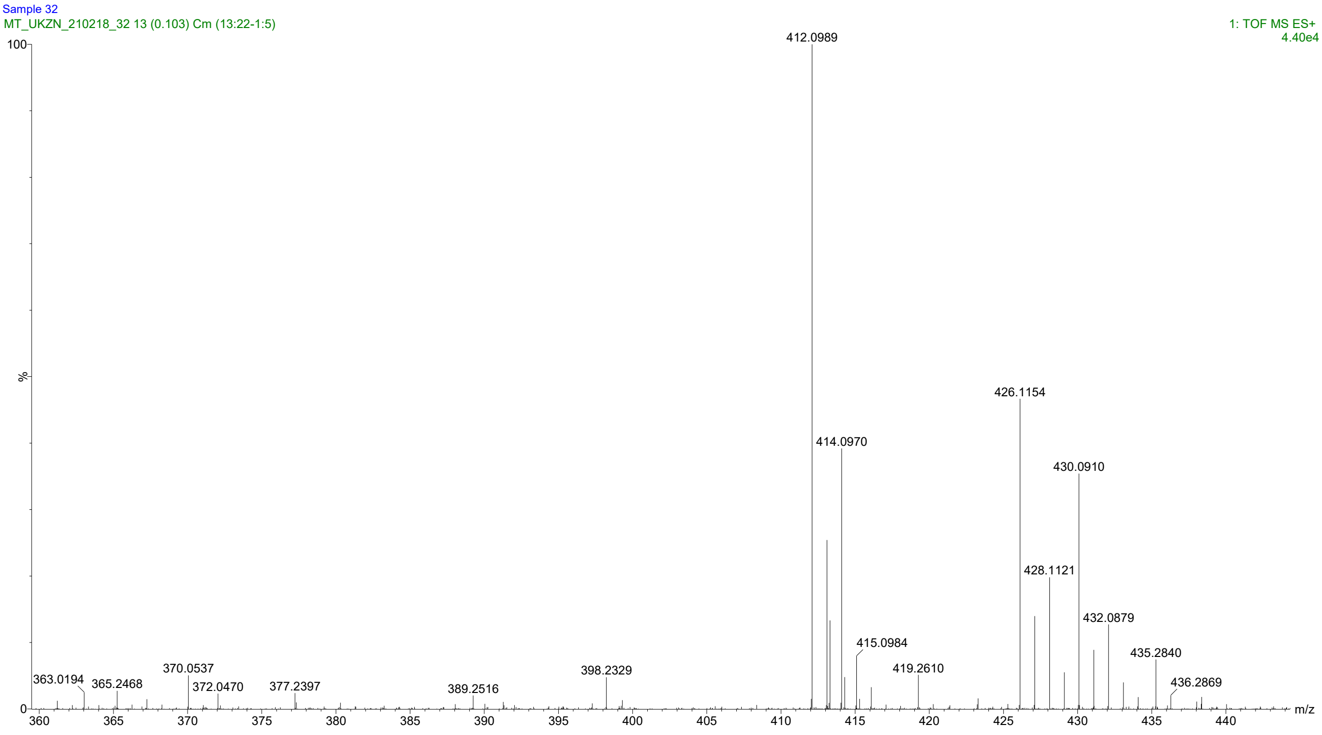

Figure S65: HRMS (ES$^+$) for *1-(1-((7-chloroquinolin-4-yl)amino)propan-2-yl)-3-(6-fluorobenzo[d]thiazol-2-yl)urea* (**6v**).

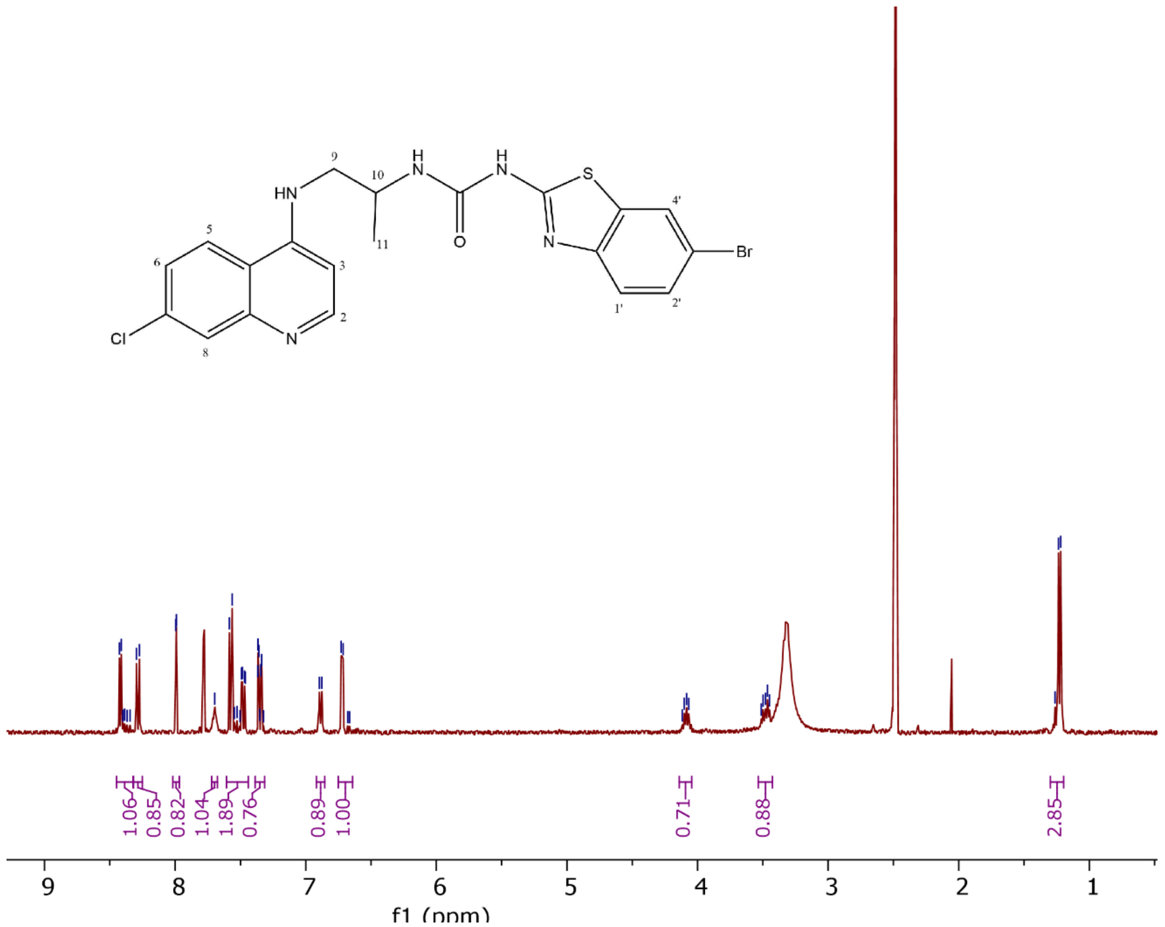

Figure S66: 400 MHz ^{1}H-NMR of *1-(1-((7-chloroquinolin-4-yl)amino)propan-2-yl)-3-(6-bromobenzo[d]thiazol-2-yl)urea* (***6w***).

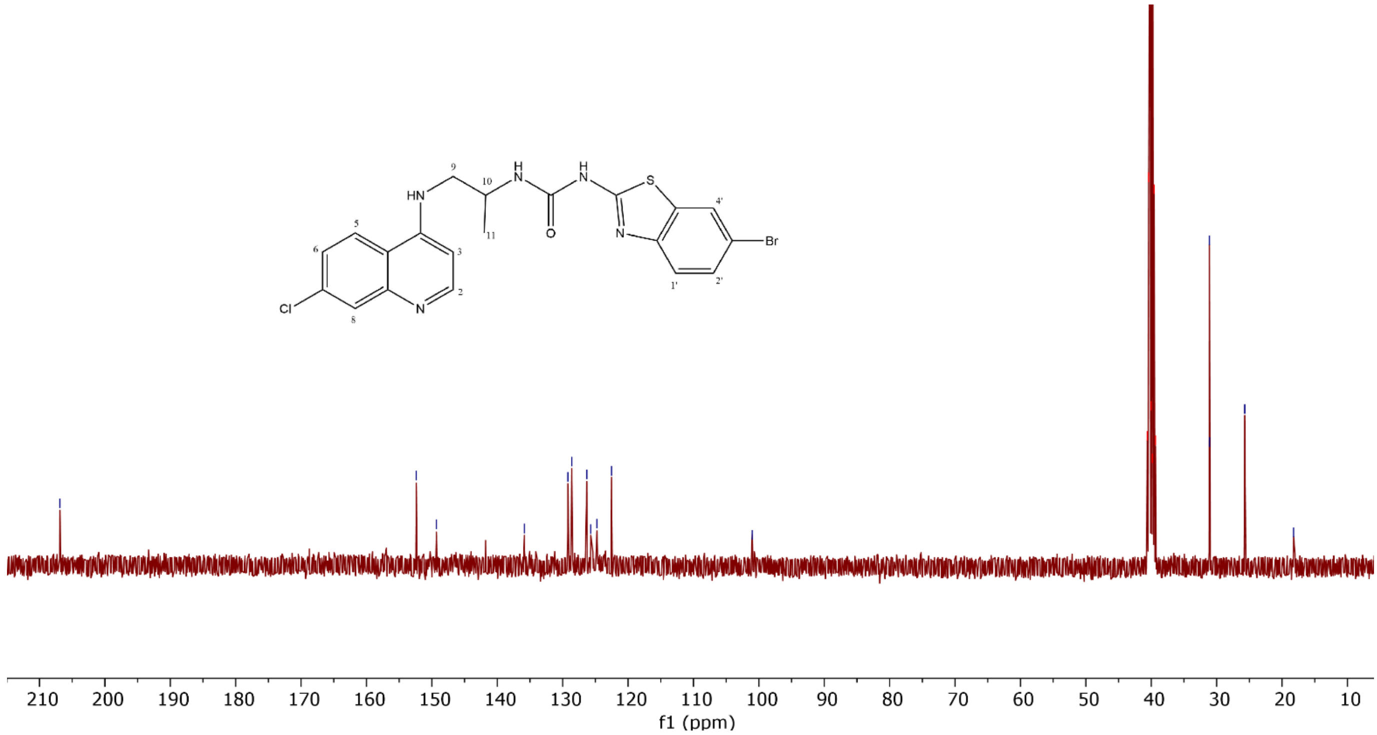

Figure S67: 101 MHz ^{13}C-NMR of *1-(1-((7-chloroquinolin-4-yl)amino)propan-2-yl)-3-(6-bromobenzo[d]thiazol-2-yl)urea* in DMSO-d6 (***6w***).

``````````

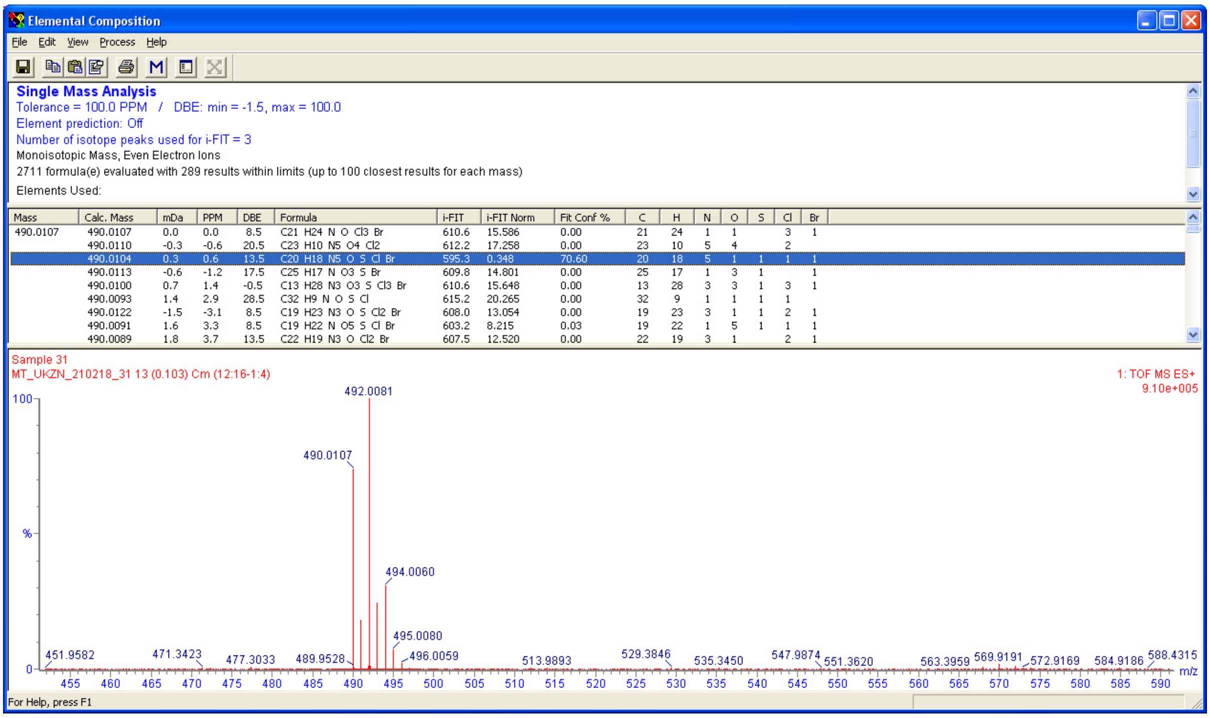


| Mass | Calc. Mass | mDa | PPM | DBE | Formula | i-FIT | i-FIT Norm | Fit Conf % | C | H | N | O | S | Cl | Br |
|---|---|---|---|---|---|---|---|---|---|---|---|---|---|---|---|
| 490.0107 | 490.0107 | 0.0 | 0.0 | 8.5 | C21 H24 N O Cl3 Br | 610.6 | 15.586 | 0.00 | 21 | 24 | 1 | 1 | | 3 | 1 |
| | 490.0110 | -0.3 | -0.6 | 20.5 | C23 H10 N5 O4 Cl2 | 612.2 | 17.258 | 0.00 | 23 | 10 | 5 | 4 | | 2 | |
| | 490.0104 | 0.3 | 0.6 | 13.5 | C20 H18 N5 O S Cl Br | 595.3 | 0.348 | 70.60 | 20 | 18 | 5 | 1 | 1 | 1 | 1 |
| | 490.0113 | -0.6 | -1.2 | 17.5 | C25 H17 N O3 S Br | 609.8 | 14.801 | 0.00 | 25 | 17 | 1 | 3 | 1 | | 1 |
| | 490.0100 | 0.7 | 1.4 | -0.5 | C13 H28 N3 O3 S Cl3 Br | 610.6 | 15.648 | 0.00 | 13 | 28 | 3 | 3 | 1 | 3 | 1 |
| | 490.0093 | 1.4 | 2.9 | 28.5 | C32 H9 N O S Cl | 615.2 | 20.265 | 0.00 | 32 | 9 | 1 | 1 | 1 | 1 | |
| | 490.0122 | -1.5 | -3.1 | 8.5 | C19 H23 N3 O S Cl2 Br | 608.0 | 13.054 | 0.00 | 19 | 23 | 3 | 1 | 1 | 2 | 1 |
| | 490.0091 | 1.6 | 3.3 | 8.5 | C19 H22 N O5 S Cl Br | 603.2 | 8.215 | 0.03 | 19 | 22 | 1 | 5 | 1 | 1 | 1 |
| | 490.0089 | 1.8 | 3.7 | 13.5 | C22 H19 N3 O Cl2 Br | 607.5 | 12.520 | 0.00 | 22 | 19 | 3 | 1 | | 2 | 1 |

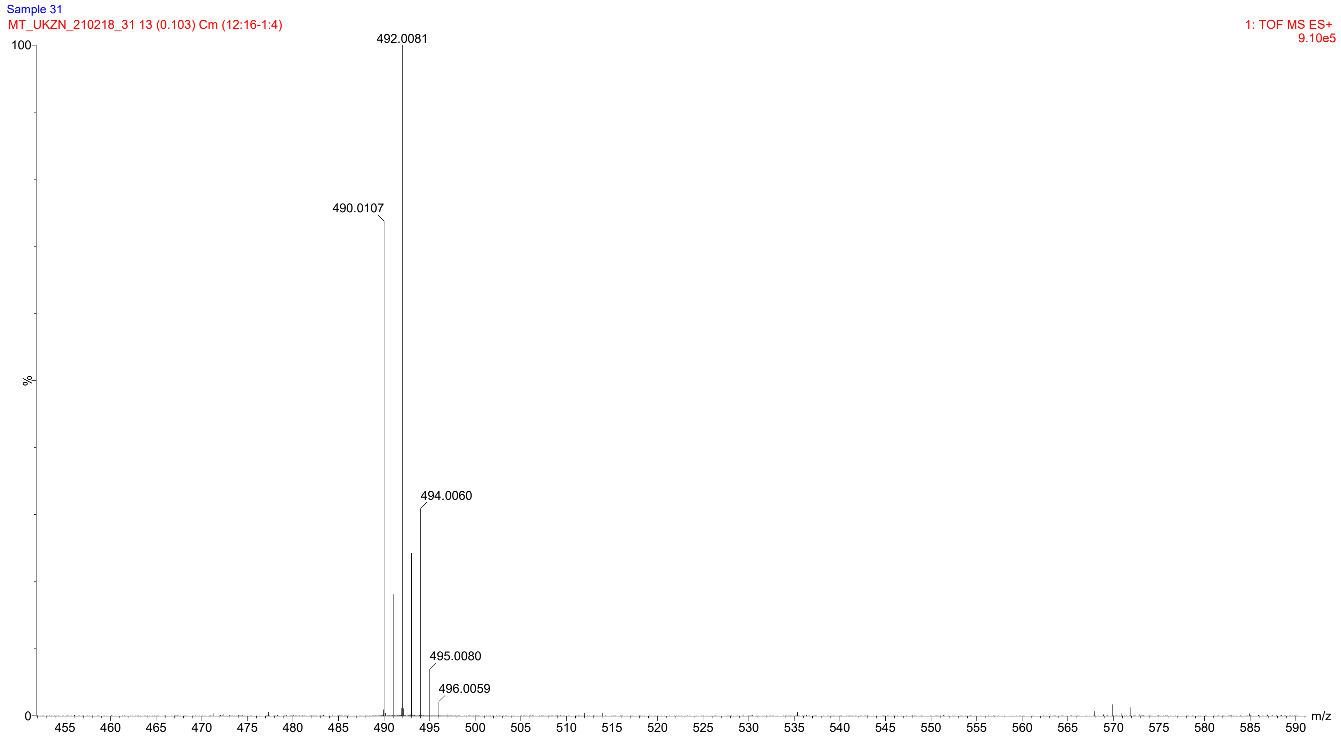


**Figure S68**: HRMS (ES$^+$) for *1-(1-((7-chloroquinolin-4-yl)amino)propan-2-yl)-3-(6-bromobenzo[d]thiazol-2-yl)urea* (**6w**).
``````````

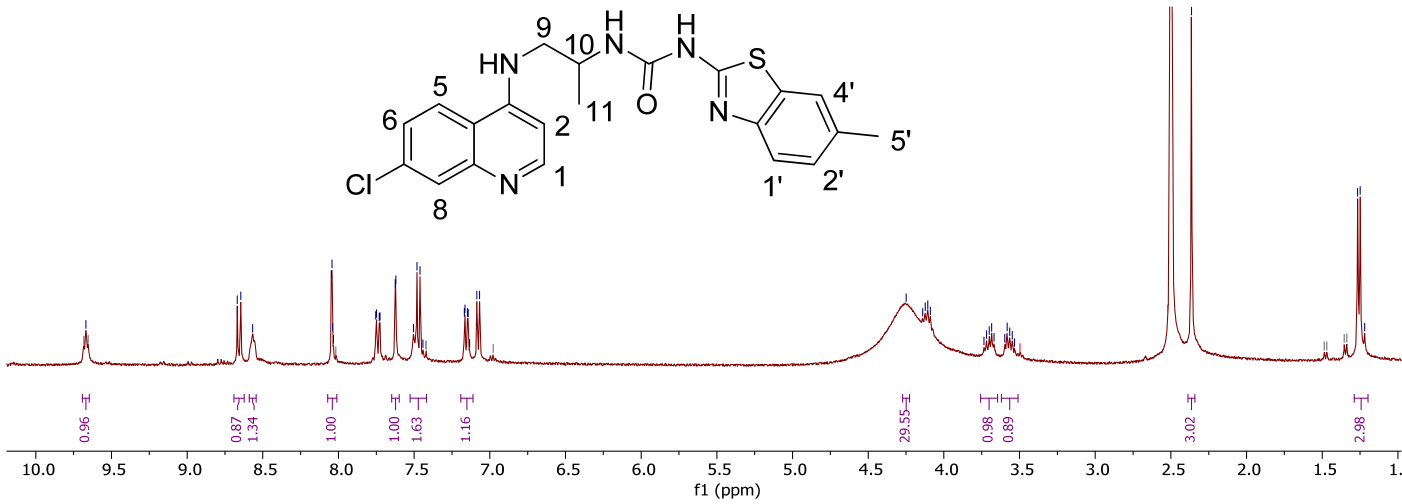

Figure S69: 400 MHz ^{1}H-NMR of *1-(1-((7-chloroquinolin-4-yl)amino)propan-2-yl)-3-(6-methylbenzo[d]thiazol-2-yl)urea* in DMSO-d6 ***(6x)***.

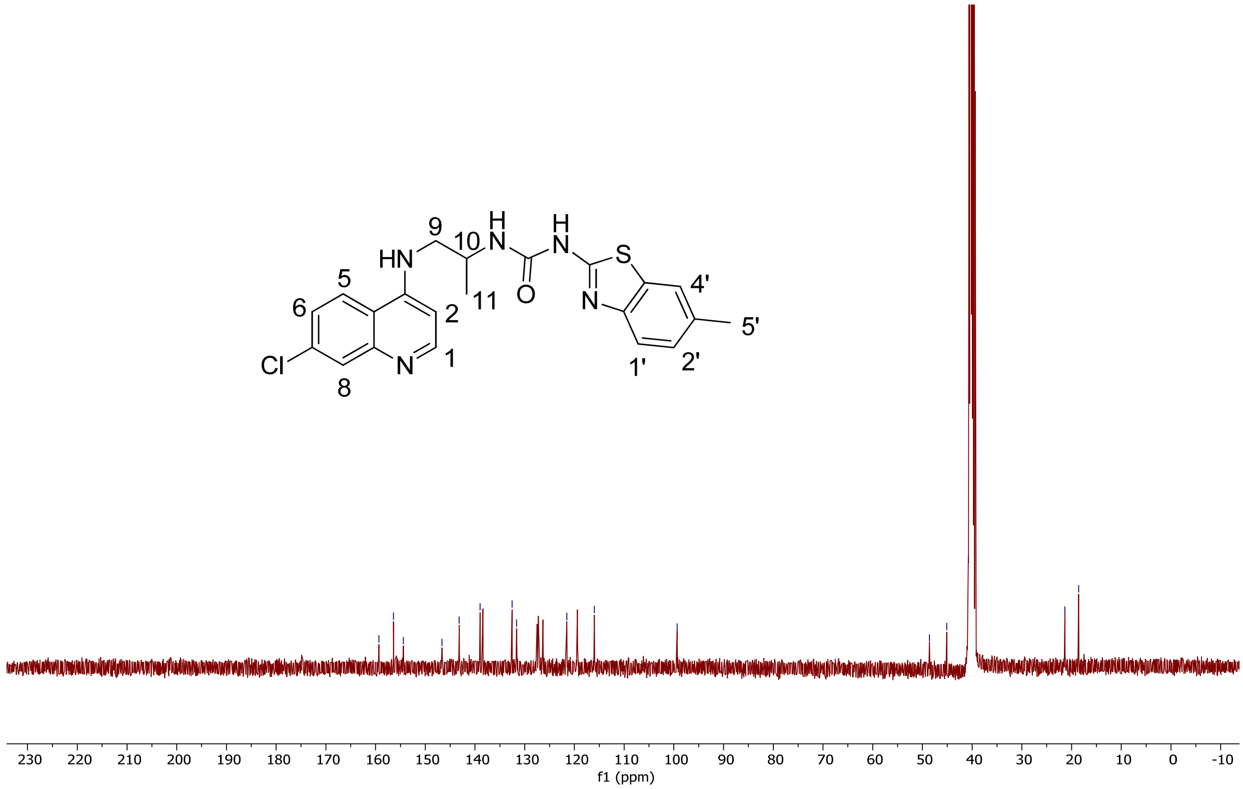

Figure S70: 101 MHz ^{13}C-NMR of *1-(1-((7-chloroquinolin-4-yl)amino)propan-2-yl)-3-(6-methylbenzo[d]thiazol-2-yl)urea* in DMSO-d6 ***(6x)***.

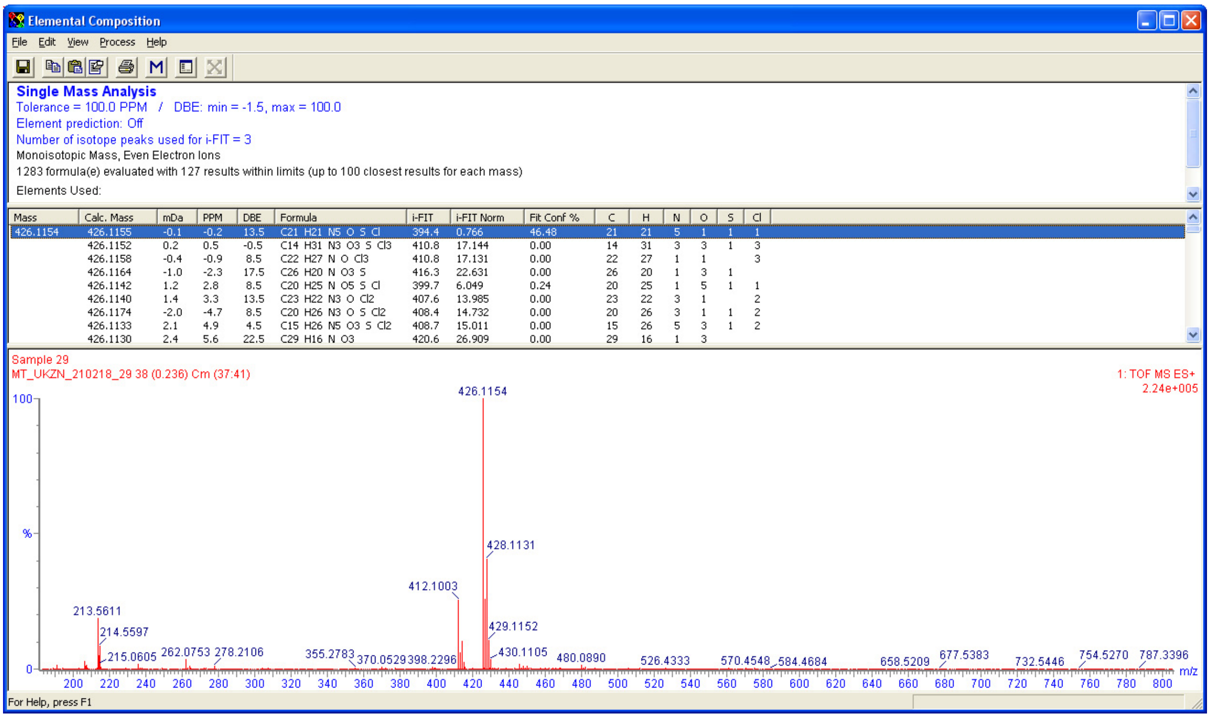

Mass	Calc. Mass	mDa	PPM	DBE	Formula	i-FIT	i-FIT Norm	Fit Conf %	C	H	N	O	S	Cl
426.1154	426.1155	-0.1	-0.2	13.5	C21 H21 N5 O S Cl	394.4	0.766	46.48	21	21	5	1	1	1
	426.1152	0.2	0.5	-0.5	C14 H31 N3 O3 S Cl3	410.8	17.144	0.00	14	31	3	3	1	3
	426.1158	-0.4	-0.9	8.5	C22 H27 N O Cl3	410.8	17.131	0.00	22	27	1	1		3
	426.1164	-1.0	-2.3	17.5	C26 H20 N O3 S	416.3	22.631	0.00	26	20	1	3	1	
	426.1142	1.2	2.8	8.5	C20 H25 N O5 S Cl	399.7	6.049	0.24	20	25	1	5	1	1
	426.1140	1.4	3.3	13.5	C23 H22 N3 O Cl2	407.6	13.985	0.00	23	22	3	1		2
	426.1174	-2.0	-4.7	8.5	C20 H26 N3 O S Cl2	408.4	14.732	0.00	20	26	3	1	1	2
	426.1133	2.1	4.9	4.5	C15 H26 N5 O3 S Cl2	408.7	15.011	0.00	15	26	5	3	1	2
	426.1130	2.4	5.6	22.5	C29 H16 N O3	420.6	26.909	0.00	29	16	1	3		

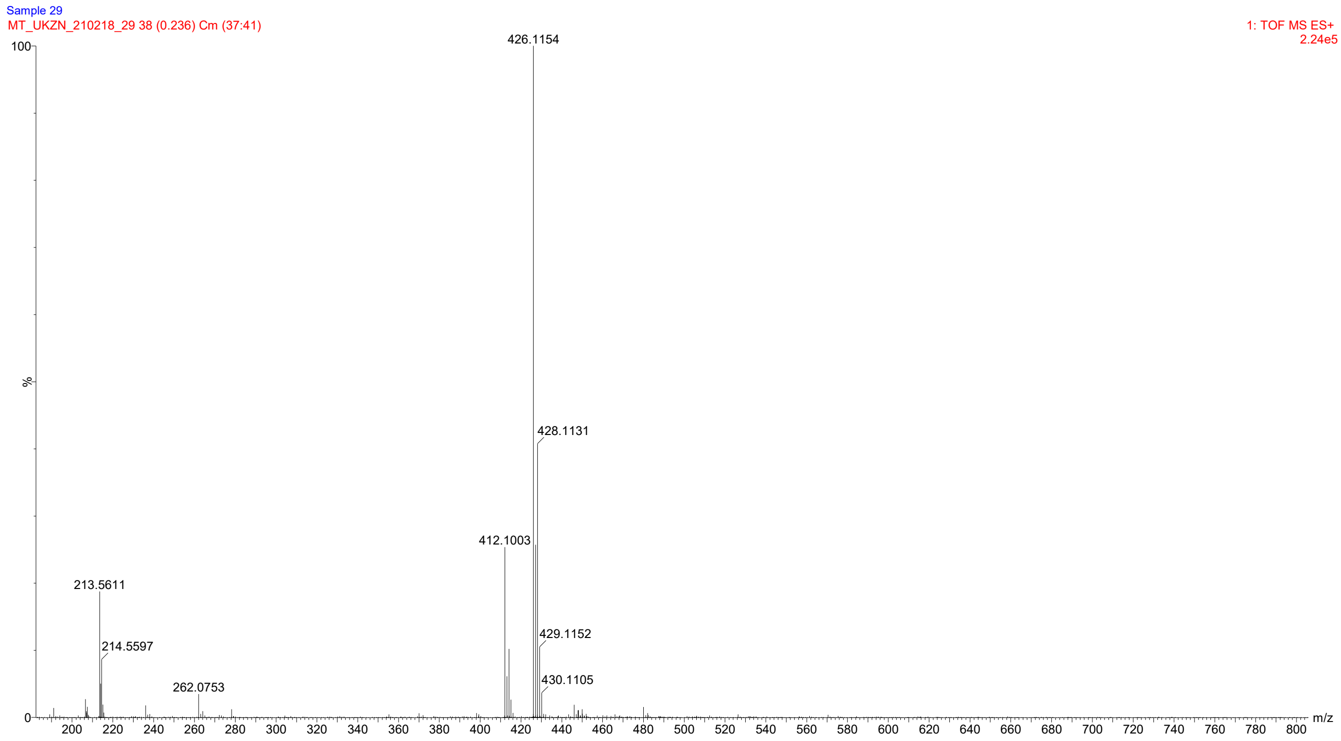

Figure S71: HRMS (ES$^+$) for *1-(1-((7-chloroquinolin-4-yl)amino)propan-2-yl)-3-(6-methylbenzo[d]thiazol-2-yl)urea* (***6x***).

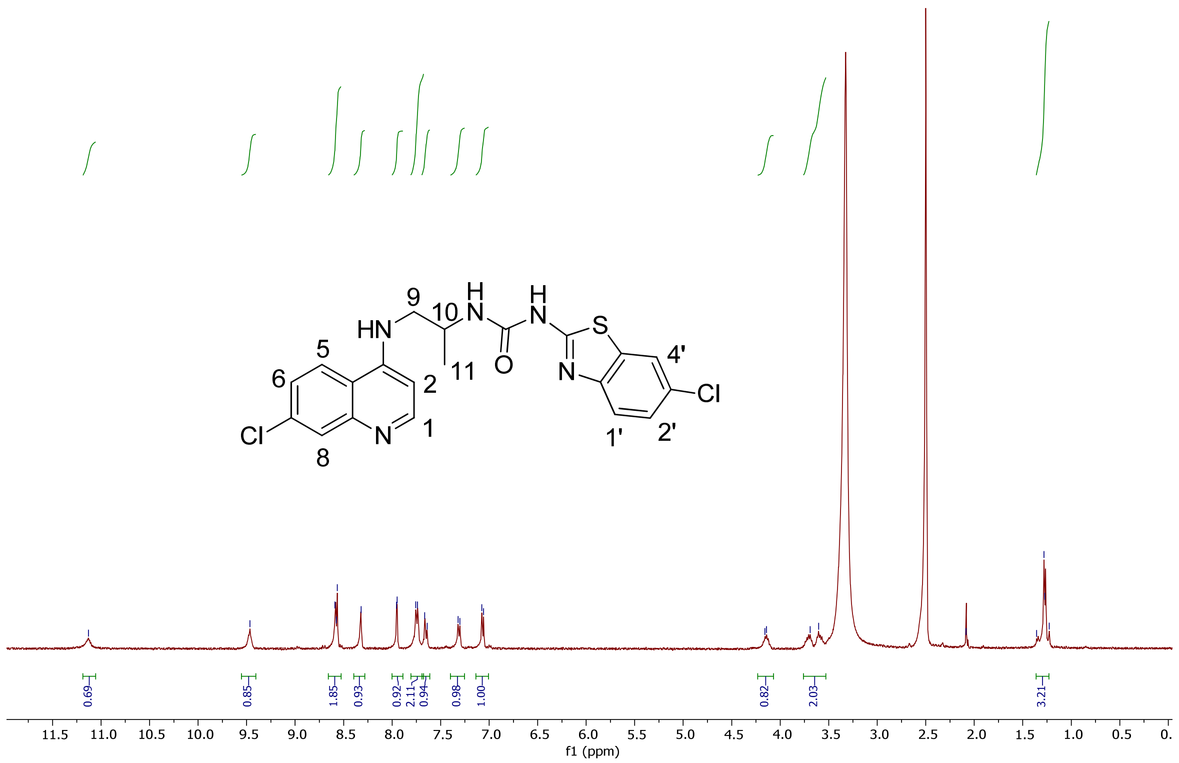

Figure S72: 400 MHz ^{1}H-NMR of *1-(1-((7-chloroquinolin-4-yl)amino)propan-2-yl)-3-(6-chlorobenzo[d]thiazol-2-yl)urea* in DMSO-d6 (**6y**).

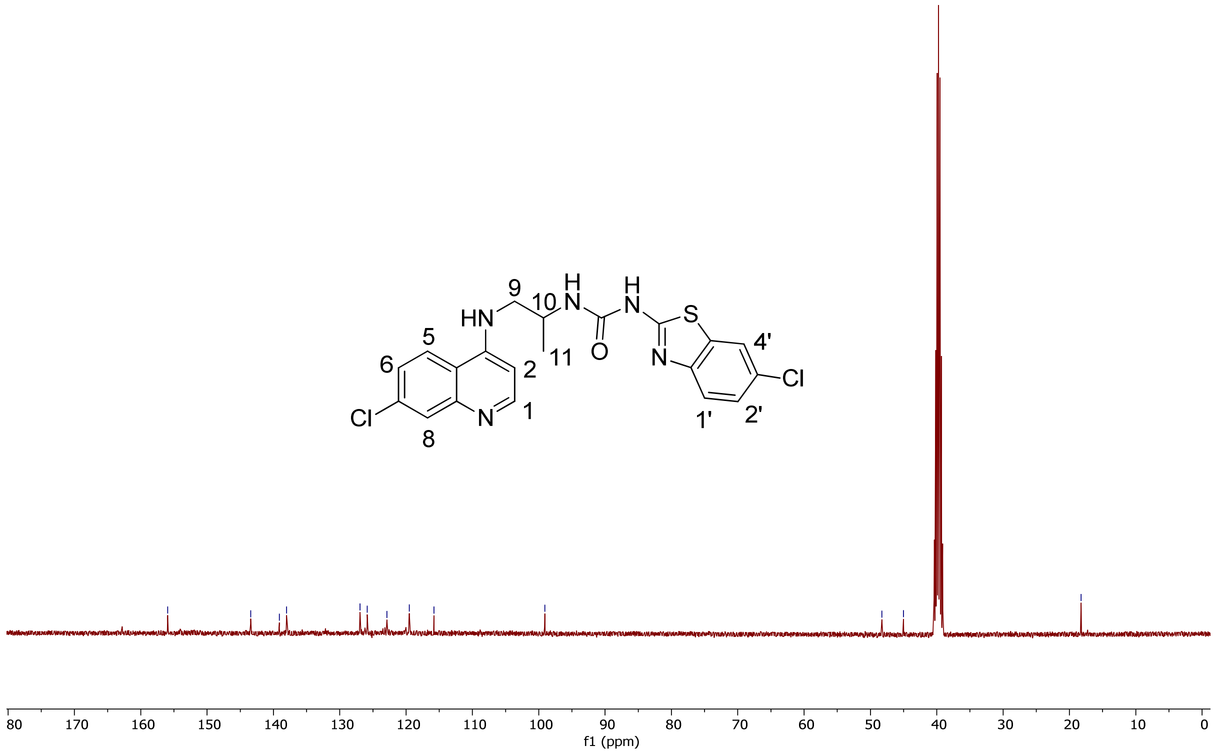

Figure S73: 101 MHz ^{13}C-NMR of *1-(1-((7-chloroquinolin-4-yl)amino)propan-2-yl)-3-(6-chlorobenzo[d]thiazol-2-yl)urea* in DMSO-d6 (**6y**).

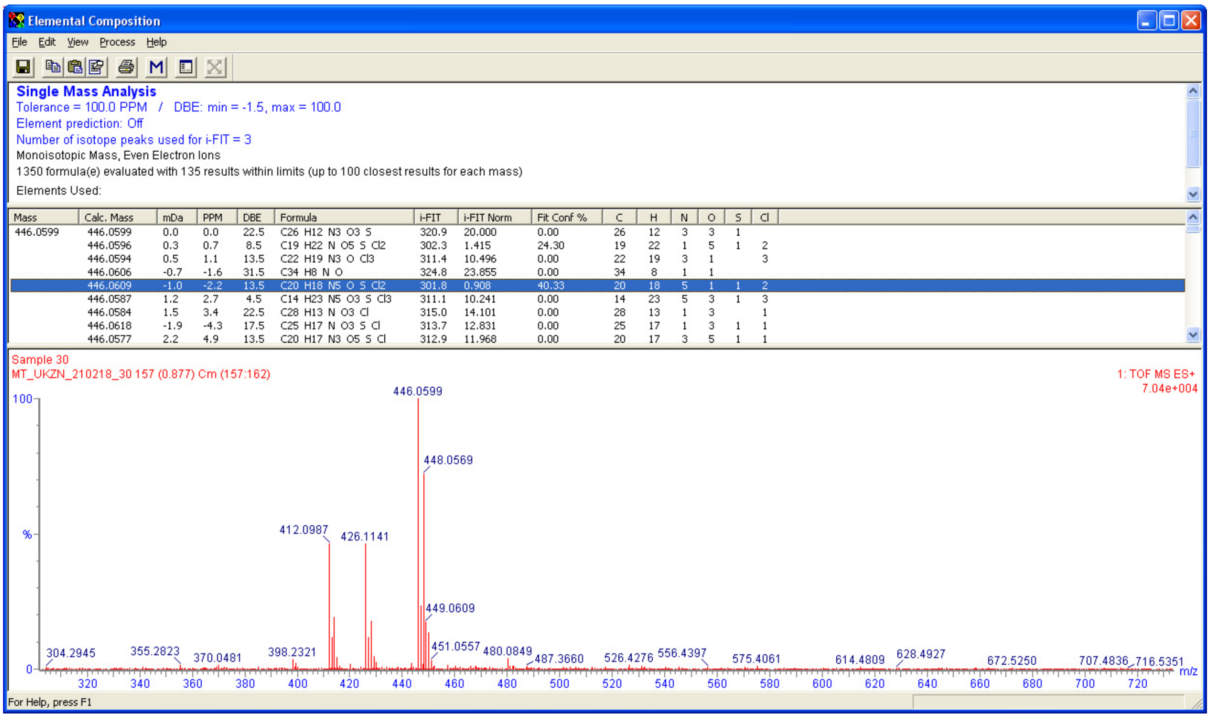

Mass	Calc. Mass	mDa	PPM	DBE	Formula	i-FIT	i-FIT Norm	Fit Conf %	C	H	N	O	S	Cl
446.0599	446.0599	0.0	0.0	22.5	C26 H12 N3 O3 S	320.9	20.000	0.00	26	12	3	3	1	
	446.0596	0.3	0.7	8.5	C19 H22 N O5 S Cl2	302.3	1.415	24.30	19	22	1	5	1	2
	446.0594	0.5	1.1	13.5	C22 H19 N3 O Cl3	311.4	10.496	0.00	22	19	3	1		3
	446.0606	-0.7	-1.6	31.5	C34 H8 N O	324.8	23.855	0.00	34	8	1	1		
	446.0609	-1.0	-2.2	13.5	C20 H18 N5 O S Cl2	301.8	0.908	40.33	20	18	5	1	1	2
	446.0587	1.2	2.7	4.5	C14 H23 N5 O3 S Cl3	311.1	10.241	0.00	14	23	5	3	1	3
	446.0584	1.5	3.4	22.5	C28 H13 N O3 Cl	315.0	14.101	0.00	28	13	1	3		1
	446.0618	-1.9	-4.3	17.5	C25 H17 N O3 S Cl	313.7	12.831	0.00	25	17	1	3	1	1
	446.0577	2.2	4.9	13.5	C20 H17 N3 O5 S Cl	312.9	11.968	0.00	20	17	3	5	1	1

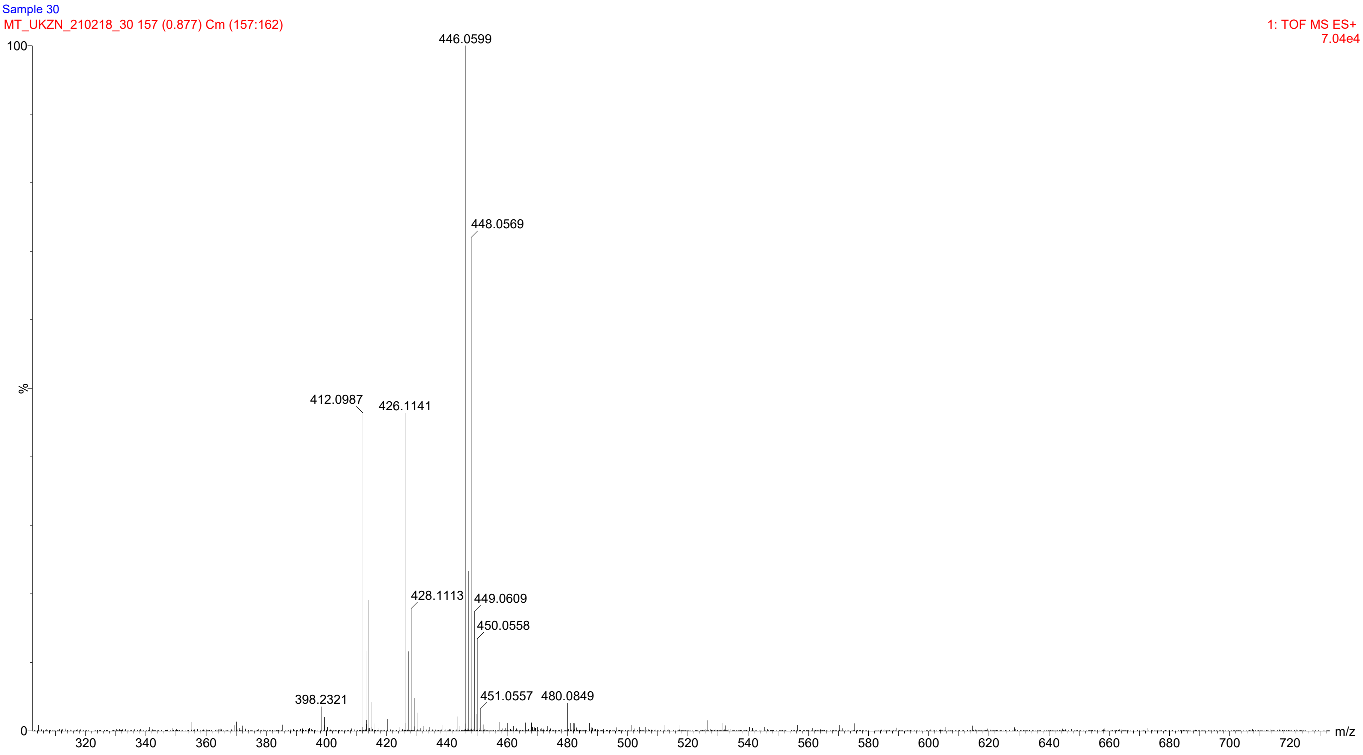

Figure S74: HRMS (ES+) for *1-(1-((7-chloroquinolin-4-yl)amino)propan-2-yl)-3-(6-chlorobenzo[d]thiazol-2-yl)urea* (**6y**).
